# Phylogeographic analysis of human influenza A and B viruses in Myanmar, 2010–2015

**DOI:** 10.1371/journal.pone.0210550

**Published:** 2019-01-10

**Authors:** Khin Thu Zar Htwe, Clyde Dapat, Yugo Shobugawa, Takashi Odagiri, Akinobu Hibino, Hiroki Kondo, Ren Yagami, Takehiko Saito, Nobuhiro Takemae, Tsutomu Tamura, Hisami Watanabe, Yadanar Kyaw, Nay Lin, Yi Yi Myint, Htay Htay Tin, Win Thein, Latt Latt Kyaw, Pan Ei Soe, Makoto Naito, Hassan Zaraket, Hiroshi Suzuki, Takashi Abe, Reiko Saito

**Affiliations:** 1 Division of International Health, Graduate School of Medical and Dental Sciences, Niigata University, Asahimachi-dori, Chuo-ku, Niigata, Niigata, Japan; 2 Department of Virology, Graduate School of Medicine, Tohoku University, 2–1 Seiryo-machi, Aoba-ku, Sendai, Miyagi, Japan; 3 Influenza and Prion Disease Research Center, National Agriculture and Food Research Organization, NARO, 3-1-1 Kannondai, Tsukuba, Ibaraki, Japan; 4 Niigata Prefectural Institute of Public Health and Environmental Sciences, 314–1 Sowa, Nishi-ku, Niigata, Niigata, Japan; 5 Infectious Diseases Research Center of Niigata University in Myanmar, Graduate School of Medical and Dental Sciences, Niigata University, Niigata, Niigata, Japan; 6 Respiratory Medicine Department, Thingangyun Sanpya Hospital, Yangon, Yangon Division, Myanmar; 7 Clinical Laboratory, Microbiology Section, Pyinmana Township Hospital, Pyinmana Township, Nay Pyi Taw, Myanmar; 8 Yangon General Hospital, Lanmadaw, Yangon, Yangon Division, Myanmar; 9 National Health Laboratory, Ministry of Health and Sports, Dagon, Yangon, Yangon Division, Myanmar; 10 Pathology department, Niigata Medical Center Hospital, Nishi-ku, Niigata, Niigata, Japan; 11 Department of Experimental Pathology, Immunology and Microbiology, Faculty of Medicine, American University of Beirut, Beirut, Lebanon; 12 Center for Infectious Diseases Research, Faculty of Medicine, American University of Beirut, Beirut, Lebanon; 13 School of Nursing and Psychology, Niigata Seiryo University, Suido-cho Chuo-ku, Niigata, Niigata, Japan; 14 Graduate School of Science and Technology, Niigata University, Nishi-ku, Niigata, Niigata, Japan; University of Alberta Department of Resource Economics and Environmental Sociology, CANADA

## Abstract

We investigated the circulation patterns of human influenza A and B viruses in Myanmar between 2010 and 2015 by analyzing full HA genes. Upper respiratory tract specimens were collected from patients with symptoms of influenza-like illness. A total of 2,860 respiratory samples were screened by influenza rapid diagnostic test, of which 1,577 (55.1%) and 810 (28.3%) were positive for influenza A and B, respectively. Of the 1,010 specimens that were positive for virus isolation, 370 (36.6%) were A(H1N1)pdm09, 327 (32.4%) were A(H3N2), 130 (12.9%) B(Victoria), and 183 (18.1%) were B(Yamagata) viruses. Our data showed that influenza epidemics mainly occurred during the rainy season in Myanmar. Our three study sites, Yangon, Pyinmana, and Pyin Oo Lwin had similar seasonality and circulating type and subtype of influenza in a given year. Moreover, viruses circulating in Myanmar during the study period were closely related genetically to those detected in Thailand, India, and China. Phylogeographic analysis showed that A(H1N1)pdm09 viruses in Myanmar originated from Europe and migrated to other countries via Japan. Similarly, A(H3N2) viruses in Myanmar originated from Europe, and disseminated to the various countries via Australia. In addition, Myanmar plays a key role in reseeding of influenza B viruses to Southeast Asia and East Asia as well as Europe and Africa. Thus, we concluded that influenza virus in Myanmar has a strong link to neighboring Asian countries, Europe and Oceania.

## Introduction

Influenza viruses are major causes of respiratory infections worldwide and represent a major threat to public health due to annual epidemics and their potential to cause pandemics [1, 2]. Monitoring of circulation patterns of influenza viruses is essential for the yearly planning of prevention and response activities in each country [3]. Influenza circulation occurs all year round in East and Southeast Asian countries but with increased activity during the rainy season [4]. Previous studies revealed that influenza activity in Thailand and Singapore usually starts in June and ends in September [5–8].

There are only a few reports on the molecular epidemiology of human influenza A(H1N1)pdm09, A(H3N2) and B in Southeast Asia [5]. An analysis of the global circulation patterns of influenza viruses revealed that human A(H3N2) and A(H1N1) epidemics strains originate from Southeast Asia and are seeded into the temperate regions [9]. Although A(H1N1) and B viruses play a limited role in new variants disseminating, A(H3N2) were reseeded from East and Southeast Asia [10]. Therefore, analysis of the genetic relationships among influenza viruses circulating in Myanmar and its surrounding countries is important due to its implications on the global influenza virus circulation.

Previously, we reported a temporal synchronicity of seasonal A(H1N1) and B strains circulating in Myanmar and Southeast Asia and China [5]. In 2008, the oseltamivir-resistant seasonal A(H1N1) virus was detected at a lower rate (6%) in Myanmar and emerged at least 2 months after its circulation in the neighboring countries. Similarly, the detection of A(H1N1)pdm09 virus did not occur in Myanmar until August 2009 [11]. We reported that influenza A(H3N2) viruses isolated in Myanmar from 2007 to 2008 exhibited reduced sensitivity to both zanamivir and amantadine. These rare and naturally occurring viruses harbored a novel Q136K mutation in neuraminidase and S31N mutation in M2 [12].

In this study, we investigated the epidemiology and genetic characteristics of influenza viruses circulating in Myanmar during January 2010 to December 2015 and compared them to those circulating in the neighboring countries. Since data on the spread of virus is limited in the region, this study analyzed the spatio-temporal phylogenetics of influenza viruses in Myanmar and the region.

## Material and methods

### Collection of clinical samples

Nasopharyngeal swabs were collected from outpatients who visited Thingangyun Sanpya General Hospital in Yangon and Pyinmana 200 Bedded General Hospital in Pyinmana, Myanmar from January 2010 to December 2015. In addition, specimens were obtained from outpatients in Pyin Oo Lwin General Hospital from January to December in 2014. The inclusion criteria included fever (>37.8°C) in addition to at least one of the following symptoms: cough, rhinorrhea, myalgia, arthralgia, and diarrhea. The swabs were screened for influenza A and B by using the Quick Navi-Flu+RSV rapid diagnostic test (RDT) Denka Seiken Co. Ltd., Tokyo, Japan. Details of the sampling method have been described previously [5, 13]. Informed consents were obtained from the patients or their guardians before swab collection. The samples were kept at -80°C until being shipped to the Division of International Health, Niigata University Graduate School of Medical and Dental Sciences, Japan for further analyses.

### Viral isolation

All swab samples were inoculated onto Madin Darby Canine Kidney (MDCK) cells for influenza virus isolation as previously described [5]. The swabs were passaged up two times to obtain enough virus titers to perform virus identification and prepare a stock of each sample.

### RNA extraction and cDNA synthesis

Viral RNA was extracted from 100 μL of supernatant of viral culture media using Extragen II kit (Tosoh Co., Ltd) following the manufacturer’s instructions. Viral RNA was then transcribed into cDNA using the Uni12 and Uni11 influenza A and B primers, respectively [5, 14].

### Typing and subtyping of influenza virus

For influenza virus typing and subtyping, real-time RT-PCR was performed by using the cycling probe method for A(H1N1)pdm09, A(H3N2), and influenza B [15, 16]. The lineage of influenza B viruses was determined using the primers listed in S1 Table.

### RT-PCR and nucleotide sequencing

The HA genes of A(H1N1)pdm09, A(H3N2) and influenza B viruses were amplified for sequencing analysis by using gene-specific primers by reverse-transcription polymerase chain reaction (RT-PCR) [5]. The PCR amplicons were purified using QIA quick PCR Purification Kit, (QIAGEN, Inc.). Sequencing reactions were carried out using Big Dye Terminator v3.1 cycle sequencing kit (Applied Bio systems, Carlsbad, USA) and the sequencing products were run on an ABI Prism 3130xl Genetic Analyzer.

### Sequence analysis

Phylogenetic tree analysis was carried out using the sequences of the HA genes from 43 A(H1N1)pdm09, 50 A(H3N2) and 46 influenza B isolates from Myanmar. Also included in the analyses were 3–5 HA sequences for each subtype per year from Asia, Africa, Europe, North America, South America, and Oceania. These sequences were selected by using the random number generator in Microsoft Excel. The sequence data used in this study comprised of 404 A(H1N1)pdm09, 406 A(H3N2), and 388 influenza B from 2010–2015, which included sequences from Myanmar and representative strains from around the world with known sample collection date (S2 and S3 Tables). Nucleotide sequences of the HA genes that were included in the analysis were downloaded from the NCBI Influenza Virus Resource (http://www.ncbi.nlm.nih.gov/genomes/FLU/) and the GISAID (http://gisaid.org/).

### Phylogeographic analysis

The temporal structure of the sequence data was tested using TempEst software (http://tree.bio.ed.ac.uk/software/tempest/) [17]. To infer the genetic evolution of influenza strains that circulated in Myanmar and to reconstruct the viral migration events by discrete phylogeography, the BEAST package version 1.8.4 was used and analyzed using the BEAGLE library(https://github.com/beast-dev/beast-mcmc/releases). The nucleotide substitution model was SRD06 + Gamma. Gaussian Markov random field (GMRF) Bayesian skyride [18] and Bayesian skyline [19] models were compared as coalescent prior under uncorrelated log-normal relaxed clock or strict clock. Phylogeographic reconstruction was performed using the symmetric discrete trait evolution model [20]. The Markov Chain Monte Carlo (MCMC) chains were run for 500 million iterations, with subsampling at every 50,000 iterations. A 10% burn-in was discarded and maximum clade credibility (MCC) tree was summarized using Tree annotator v1.8.3, with Bayesian posterior probability (BPP) values for each node [20, 21]. The convergence and effective sample size (ESS) were analyzed using TRACER version 1.6. (http://tree.bio.ed.ac.uk/software/tracer/) and ESS values of ≥200 were accepted. A maximum clade credibility tree was generated and visualized using FigTree version 1.4.2 (http://tree.bio.ed.ac.uk/software/Figtree). The strains were classified into clades following World Health Organization (WHO) definition (https://www.crick.ac.uk/partnerships/worldwide-influenza-centre/annual-and-interim-reports). The phylogeographic inferences of influenza virus using HA segments were analyzed and visualized with the spatial phylogenetic reconstruction of evolutionary dynamics using data-driven documents (SpreaD3) version 0.9.7.1 (https://rega.kuleuven.be/cev/ecv/software/SpreaD3) [22]. To visualize the geographic migration of the virus over time, a keyhole markup language (KML) file was generated using SpreaD3 v0.9.7.1, which was visualized via Google Earth V (www.google.com/earth/). We used world geo.json file which is public domain as https://github.com/johan/world.geo.json/blob/master/UNLICENSE.

The resulting log files were used to calculate BF values for significant diffusion rates between discrete locations. The degree of support rates was indicated as decisive for BF>1000, very strong support for 100<BF<1000, strong support for 10<BF<100, and support for 3<BF<10 [23]. Branches in the Bayesian MCC tree along which location transitions occur was visualized as lines using the SPREAD3 software including Bayes Factor (BF) test for the decisive support and posterior probability over 0.3 in global maps.

### Nucleotide sequence accession numbers

Nucleotide sequences determined in this study were submitted to the GISAID database and assigned the accession numbers EPI1002260 to EPI1002693 for the HA genes of the A(H1N1)pdm09 isolates, EPI1002701 to EPI1002743 for the A(H3N2) isolates, and EPI1002745 to EPI568165 for the influenza B viruses.

### Ethical Statement

This study was approved by the Niigata University Ethical Committee (2533) and the Ethical Review Committee in Department of Medical Research, Ministry of Health and Sports, Myanmar. Written consent was obtained from all study participants prior to sample collection.

## Results

A total of 2,860 nasopharyngeal samples were collected from the patients who fulfilled the inclusion criteria in Yangon, Pyinmana and Pyin Oo Lwin, Myanmar during 2010–2015. Of these, 1,577 (55.1%) were diagnosed as influenza A, 810 (28.3%) were influenza B, and the rest (n = 473; 16.5%) were negative by using the RDT. Virus isolation was performed on the influenza-positive specimens. Consequently, a total of 1,010 (42.3%) influenza viruses were isolated (Table 1).

**Table 1:** Yearly distribution of samples and influenza virus isolates in Myanmar, 2010–2015.

	2010		2011		2012		2013		2014		2015		Total	
	n = 689		n = 455		n = 521		n = 313		n = 533		n = 349		n = 2860	
	689		401		521		203		443		130		2387	
Virus isolate (+)	356	(51.7%)	173	(43.1%)	101	(19.4%)	63	(31.0%)	259	(58.5%)	58	(44.6%)	1010	(42.3%)
A (H1N1) pdm09	228	(64.0%)	0	(0.0%)	18	(17.8%)	1	(1.6%)	92	(35.5%)	31	(53.4%)	370	(15.5%)
A (H3N2)	18	(5.1%)	165	(95.4%)	0	(0.0%)	62	(98.4%)	55	(21.2%)	27	(46.6%)	327	(13.7%)
B (Victoria)	110	(30.9%)	8	(4.6%)	12	(11.9%)	0	(0.0%)	0	(0.0%)	0	(0.0%)	130	(5.4%)
B(Yamagata)	0	(0.0%)	0	(0.0%)	71	(70.3%)	0	(0.0%)	112	(43.2%)	0	(0.0%)	183	(7.7%)

*RDT- Rapid Diagnostic Test

**Fig 1:**
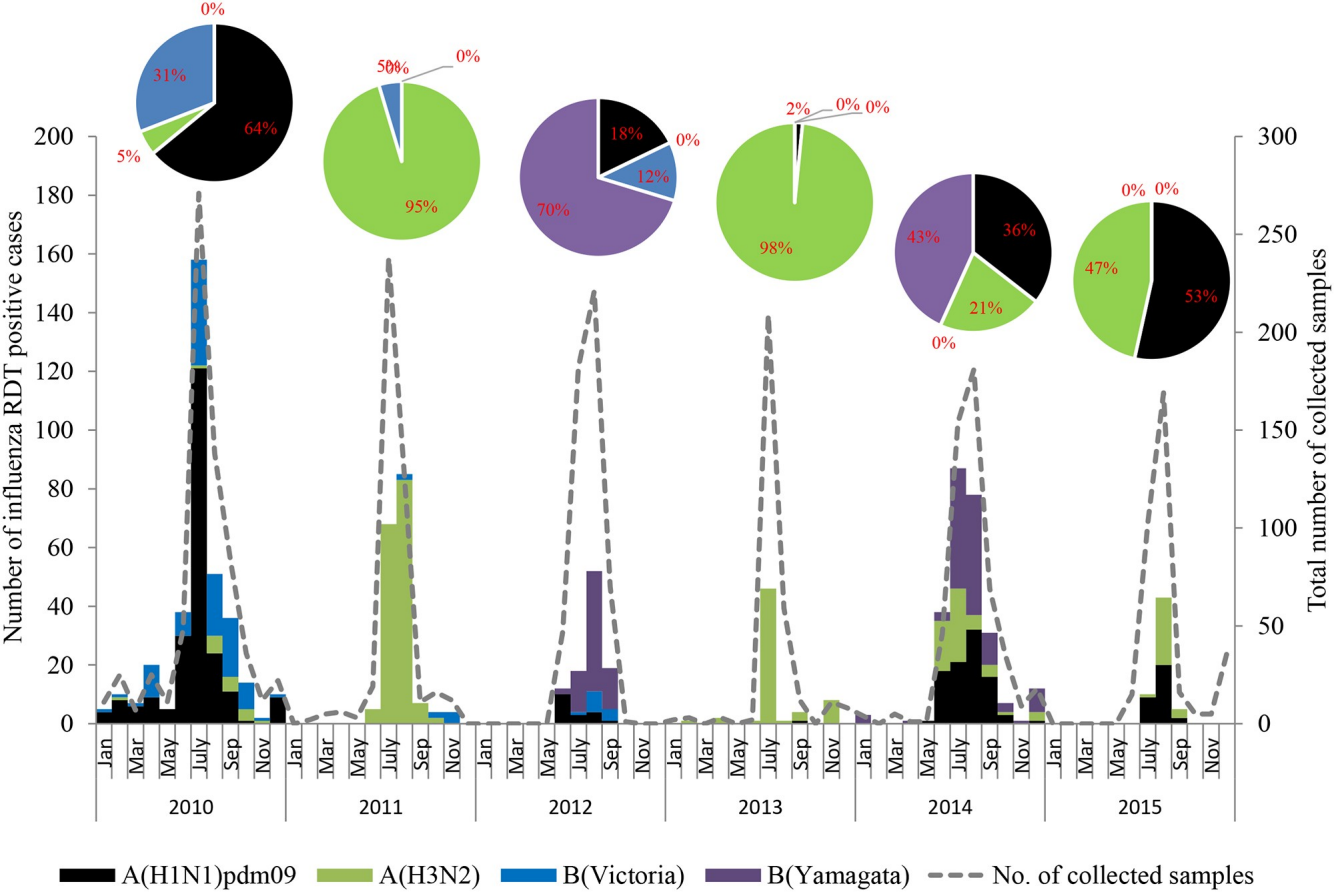
The monthly incidence of influenza A(H1N1)pdm09, A(H3N2), B(Victoria) and B(Yamagata) viruses between 2010 and 2015 in Myanmar. The number of influenza RDT positive cases is shown as a dashed line. Influenza A(H1N1)pdm09 is shown as black bar; A(H3N2), green bar; B(Victoria), blue bar; B(Yamagata), purple bar. The percentage of influenza A subtypes and B lineages for each year are shown in pie chart.

The monthly distribution of isolated influenza viruses showed that the influenza season in Myanmar occurred between May through September and peaked during July or August (Fig 1). The dominant type and subtype of circulating influenza virus varied each year. Influenza A(H1N1)pdm09 was predominant during 2010 (228/356, 64.0%) and 2015 (31/58, 53.4%). Influenza A(H3N2) viruses represented the majority of circulating influenza viruses during 2011 (165/173, 95.4%) and 2013 (62/63, 98.4%) while B(Yamagata) lineage was the most prevalent virus during 2012 (71/101, 70.3%) and 2014 (112/259, 43.2%).

**Fig 2:**
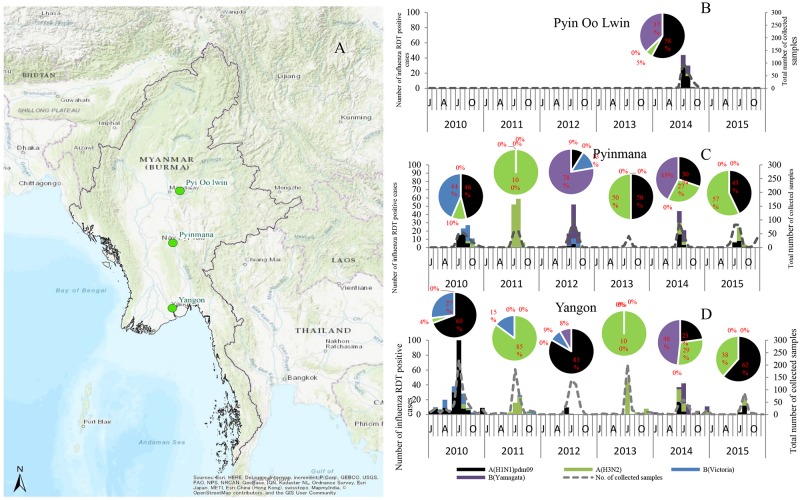
The location of sample collection sites and monthly distribution of influenza isolates according to study sites between 2010 and 2015 in Myanmar. (A) The map of Myanmar showing three study sites, Yangon, Pyinmana and Pyin Oo Lwin. Monthly distribution of influenza for three locations, (B) Pyin Oo Lwin, (C) Pyinmana and (D) Yangon. The number of influenza RDT positive cases is shown in dashed line. Influenza A(H1N1)pdm09 is shown as black bar; A(H3N2), green bar; B(Victoria), blue bar; B(Yamagata), purple bar. The percentage of influenza A subtype and B lineage in each year is shown in pie chart. This map is designed to be used as a base map by GIS professionals and as a reference map by anyone. The map includes administrative boundaries, cities, water features, physiographic features, parks, landmarks, highways, roads, railways, and airports overlaid on land cover and shaded relief imagery for added context. Sources: Esri, HERE, Garmin, Intermap, increment P Corp., GEBCO, USGS, FAO, NPS, NRCAN, GeoBase, IGN, Kadaster NL, Ordnance Survey, Esri Japan, METI, Esri China (Hong Kong), swisstopo, OpenStreetMap contributors, and the GIS User Community.

Monthly distribution of influenza virus according to study sites, Yangon, Pyinmana and Pyin Oo Lwin showed no differences in the circulating period and type/subtype during the study period (Fig 2).

### Time-aware phylogenetic reconstruction of the A(H1N1)pdm09, A(H3N2), and B isolates in Myanmar

The temporal structure of the HA of A(H1N1)pdm09, A(H3N2) and B virus was tested using TempEst software. Our data showed temporal signal with correlation coefficient values of 0.87 for A(H1N1)pdm09, 0.88 for A(H3N2) and 0.7 for influenza B virus (S1, S2, and S3 Figs). The high values of the coefficient correlation show a positive correlation between genetic divergence and sampling time. This indicates that the datasets are suitable for phylogenetic molecular clock analysis. We constructed time-scaled phylogeographic MCC (maximum clade credibility) trees of HA and inferred ancestral locations of each branch using collection dates and locations of the sequences. The most probable location of each branch is assigned in different colors and the time-scale is shown in the bottom of the tree.

**Fig 3:**
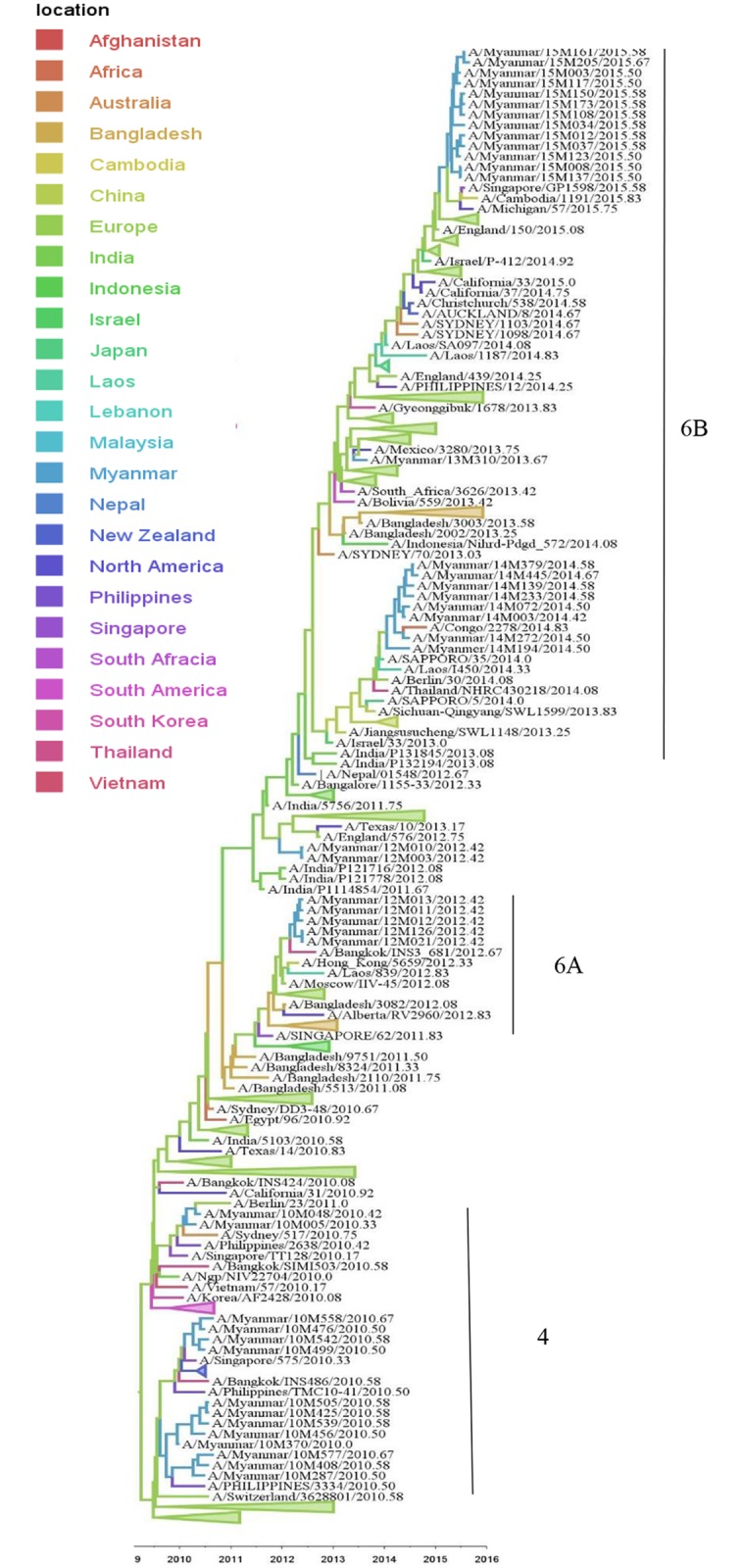
Bayesian evolutionary tree of influenza A(H1N1)pdm09 based on the nucleotide sequence of the HA gene. The maximum clade credibility tree was inferred from 404 HA gene sequences including 43 A(H1N1)pdm09 sequences from Myanmar. The branches are in time scale in years and are colored according to the location of the most probable ancestor of descendant nodes. This Bayesian tree was generated using a molecular clock discrete phylogeographic approach as implemented in the BEAST software. Labels on the right of the figure indicate the genetic clades according to WHO classification. Branches that do not include Myanmar sequences were collapsed as triangles.

During the 2010 season, all the Myanmar A(H1N1)pdm09 viruses belonged to clade 4, which also include isolates from Thailand, Singapore, Philippines, and Australia and New Zealand (Fig 3). During the 2011 season, no A(H1N1)pdm09 circulation was observed in Myanmar. In 2012, all the strains belonged to clade 6A and were closely related to the strains that circulated in Thailand, Hong Kong and Bangladesh and Europe. Myanmar A(H1N1)pdm09 isolates from 2013 to 2015 belonged to clade 6B which circulated together with the isolates from Singapore, Cambodia, North America, Australia, Africa and Europe (Fig 3). The phylogenetic tree of HA showed that the root of A(H1N1)pdm09 are most likely to be European strains. Through 2010–2015, Myanmar strains were located at the tip of the branches of the HA tree. No local persistence over one year was observed for Myanmar A(H1N1)pdm09.

**Fig 4:**
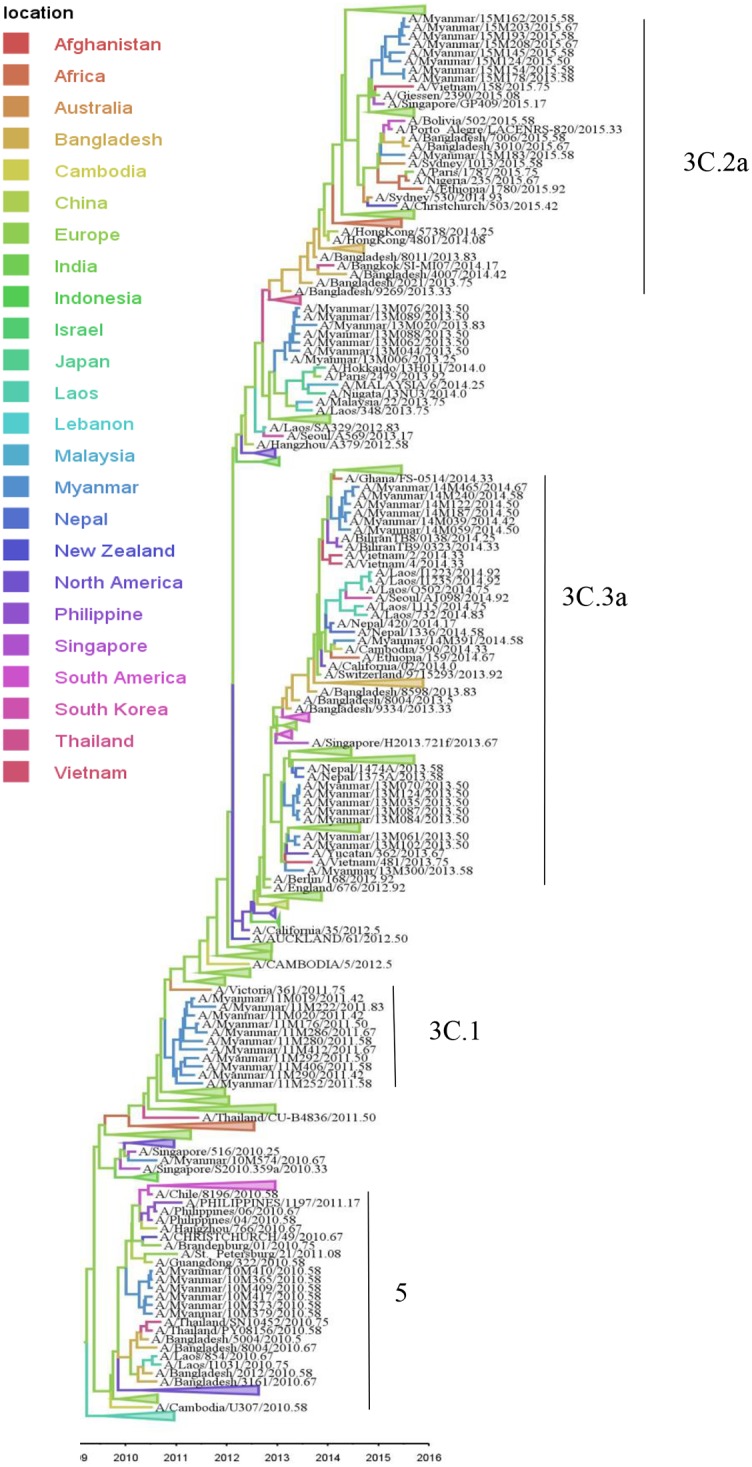
Bayesian evolutionary tree of influenza A(H3N2) based on the nucleotide sequence of the HA gene. The maximum clade credibility tree was inferred from 406 HA gene sequences including 50 A(H3N2) sequences from Myanmar. The branches are in time scale in years and are colored according to the location of the most probable ancestor of descendant nodes. This Bayesian tree was generated using a molecular clock discrete phylogeographic approach as implemented in the BEAST software. Labels on the right of the figure indicate the genetic clades according to WHO classification. Branches that do not include Myanmar strains were collapsed as triangles.

Phylogenetic tree analysis of the HA genes of A(H3N2) viruses showed that the isolates in 2010 in Myanmar belonged to clade 5 and they were closely related to viruses from Laos, Bangladesh and Thailand (Fig 4). Viruses circulating in Myanmar in 2011 belonged to clade 3C.1, which was closely related to A/Victoria/361/2011 and was included in the 2013 season vaccine (northern and southern hemisphere) [24]. The 2013 and 2014 Myanmar isolates belonged to clade 3C.3a and were genetically close to A/Texas/50/2012 and A/Switzerland/97/2013, the WHO recommended vaccine strains for the southern hemisphere in 2014 and 2015, respectively. The 2015 Myanmar isolates belonged to subclade 3C.2a that also included viruses from Vietnam, Bangladesh, Singapore, Europe, Africa, Australia and New Zealand (Fig 4). The MCC tree showed that the common ancestors of A(H3N2) were likely to be European strains but other Asian strains such as Laos, Bangladesh, Thailand, and African strains were also found in the trunk (Fig 4). Myanmar A(H3N2) strains were found in the middle of monophyletic branches showing A(H3N2) viruses could be disseminated from Myanmar to other areas but not the overall source of seeding. There was no local persistence over one year observed for Myanmar A(H3N2) strains.

**Fig 5:**
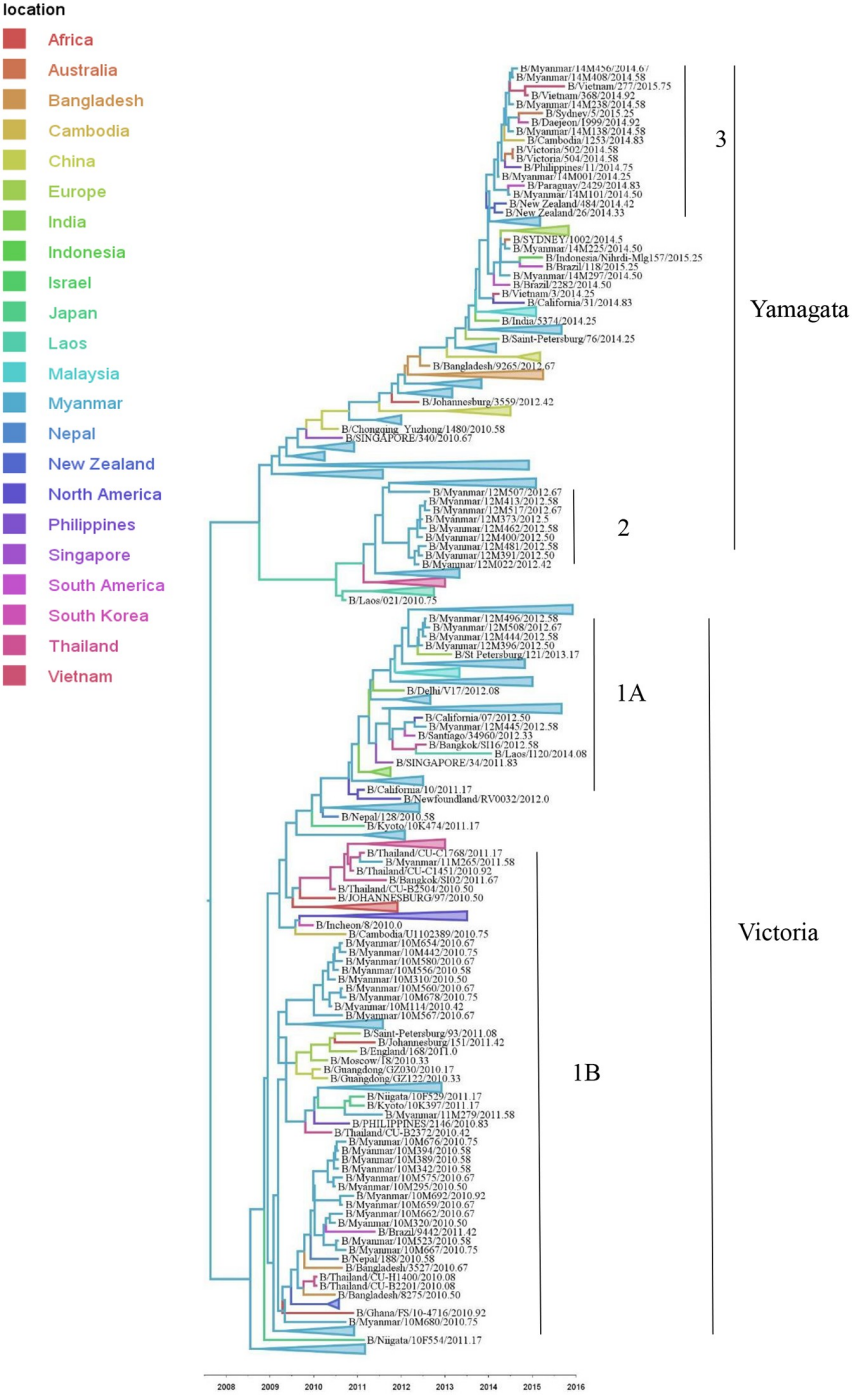
Bayesian evolutionary tree of influenza B based on the nucleotide sequence of the HA gene. The maximum clade credibility tree was inferred from 388 HA gene sequences including 46 influenza B sequences from Myanmar. The branches are in time scale in years and are colored according to the location of the most probable ancestor of descendant nodes. This Bayesian tree was generated using a molecular clock discrete phylogeographic approach as implemented in the BEAST software. Labels on the right of the figure indicate the genetic clades according to WHO classification. Branches that do not include Myanmar strains were collapsed as triangles.

The phylogenetic trees of influenza B HA sequence from Myanmar viruses showed that both B(Victoria) and B(Yamagata) lineages circulated during 2010 to 2015 (Fig 5). B(Victoria) lineage, clade 1B was detected only in 2010 and 2011 seasons in Myanmar, which circulated together with the isolates from Thailand, Malaysia, Nepal, Japan, China, South Korea, South America, Africa and New Zealand. In 2012, co-circulation of both the Victoria and Yamagata lineages was observed in Myanmar, although the latter lineage was the predominant circulating strain. Moreover, 2012 Myanmar isolates belonged to Victoria, clade 1A, which were in the same cluster with India, Thailand, Vietnam, Singapore, Europe, South America, North America, Australia and New Zealand whereas some 2012 Myanmar isolates belonged to Yamagata, clade 2 which circulated together with isolates from Thailand, Laos and Europe. In 2014, viruses belonging to the Yamagata lineage, clade 3 were the only circulating influenza B viruses in Myanmar, which were in the same cluster with isolates from the Philippines, Vietnam, Cambodia, Thailand, Bangladesh, Japan, South America, and Australia. The MCC tree showed that Myanmar strains are located near the trunk of both B(Victoria) and B(Yamagata) lineages (Fig 5). For B(Victoria), a part of trunk was India, and for B(Yamagata) a part is China and Bangladesh, but the main source could be Myanmar.

### Phylogeography of migratory patterns of Myanmar influenza viruses A(H1N1)pdm09, A(H3N2), and B

In this study, the dispersal histories of the HA segments of A(H1N1)pdm09, A(H3N2), and B among countries were inferred with a discrete phylogeographic method using BEAST software.

**Fig 6:**
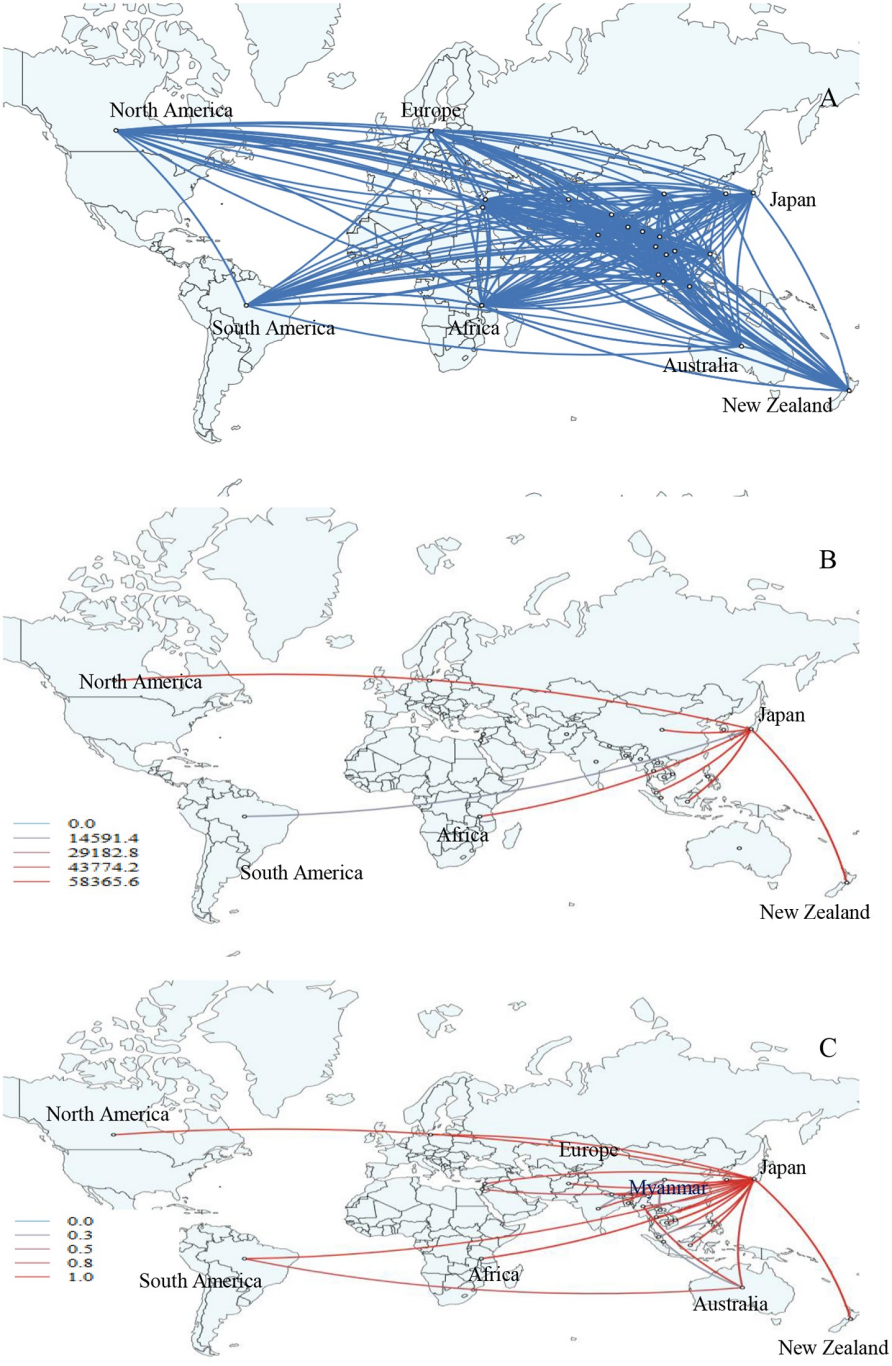
Global migration pathways of influenza A(H1N1)pdm09 viruses. (A) Spread of influenza A(H1N1)pdm09 viruses from one location to another between 2010 and 2015. Blue lines indicate the connection between different locations, which represent branches in the MCC tree. (B) The color gradients (from blue to red) of the lines indicate the relative strength of connection between locations according to the Bayes Factor (BF) test. Only rates supported by a BF greater than 1000 are indicated. (C) The color gradients (from blue to red) of the lines indicate the posterior probability of viral migration among locations. Lines were displayed when the posterior probability values were above 0.3.

According to our data, the earliest divergent events of influenza A(H1N1)pdm09 (HA) were observed from Europe to Myanmar, Southeast Asia and South Asia (Laos, and Bangladesh), China, North America, Africa, and South America during 2009–2010 (S4 Fig and S1 Movie). Our phylogeographic reconstruction supports a large number of migrations globally although the significant epidemiological linkage shows only a number of pair of regions with decisive support (BF>1000). Interestingly, the two significant migration pathways of Myanmar A(H1N1)pdm09 viruses were observed with very strong supported pathways indicating migration from Australia to Myanmar (BF = 210, posterior probability = 0.95) and a strong supported pathway from Japan to Myanmar (BF = 26, posterior probability = 0.70) (Fig 6 and S7 Table). Moreover, our results indicated that the main hub of migration during 2010–2015 was Japan. The dispersion pattern was observed from North America, Europe, Australia, India, and Lebanon to Japan and then spread from Japan to Myanmar and other Asia countries (e.g. China, Malaysia, Indonesia, and Nepal), New Zealand, Africa, and South America, which established strong migration links statistically (3<BF<1000) (Fig 6 and S7 Table). In addition, significant migration links were observed from India, Bangladesh, and Laos to China and from Australia to South America and Singapore (BF>3).

**Fig 7:**
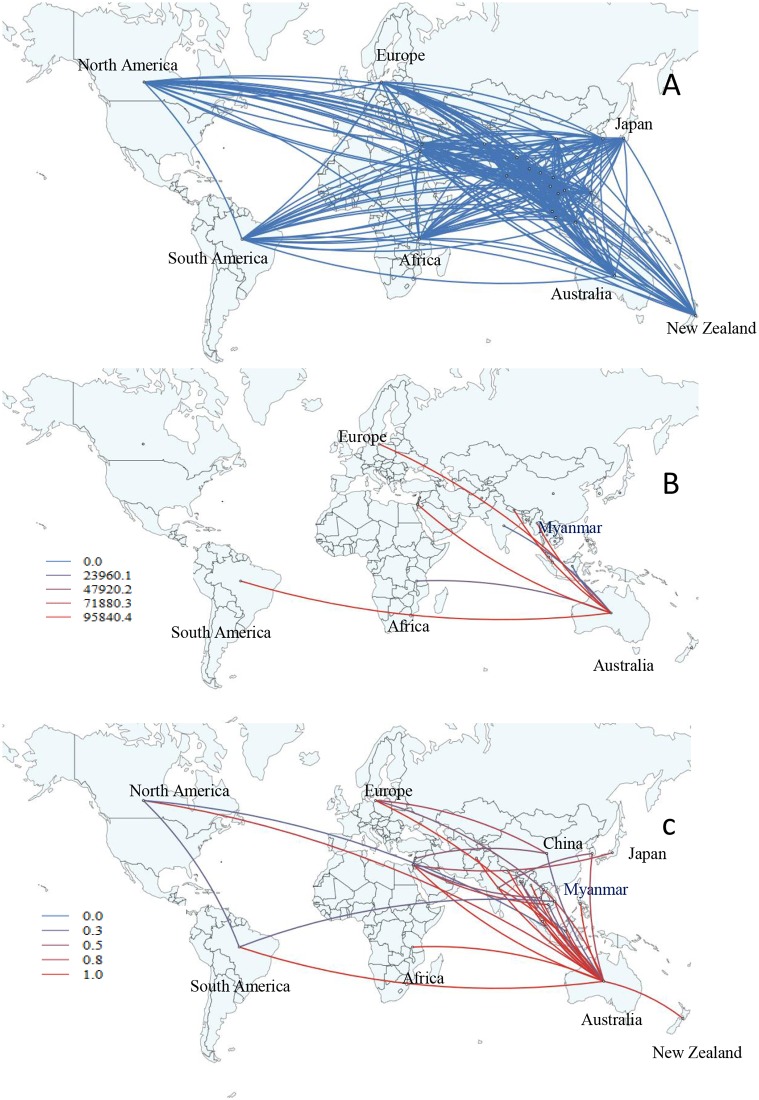
Global migration pathways of influenza A(H3N2) viruses. (A) Spread of influenza A(H3N2) viruses from one location to another between 2010 and 2015. Blue lines indicate the connection between different locations, which represent branches in the MCC tree. (B) The color gradients (from blue to red) of the lines indicate the relative strength of connection between locations according to the Bayes Factor (BF) test. Only rates supported by BF values greater than 1000 are indicated. (C) The color gradients (from blue to red) of the lines indicate the posterior probability of viral migration among locations. Lines were displayed when the posterior probability values were above 0.3.

The influenza A(H3N2) migration pathway showed a marked similarly with A(H1N1)pdm09 in our data. Viral migrations were observed from Europe to Laos, and to Myanmar and other Asian countries such as Bangladesh, Thailand, India, Philippines, and Singapore during 2009–2010 (S5 Fig and S2 Movie). In the same period, the virus migrated from Europe to Africa, North America, and South America. In Myanmar, the overall significant migration pathway with decisive support (BF>1000) was recognized with the strong epidemiological link from Australia to Myanmar (BF = 95840, posterior probability = 1) (Fig 7). For A(H3N2), the migration hub was observed in Australia. The strong migration pathway (3<BF<1000) from Europe, India, Laos, Africa, North America to Australia. Then, the viruses migrated from Australia to Myanmar, other Asian countries (e.g. Nepal, Indonesia, and the Philippines), South America, and New Zealand (BF>3) (Fig 7 and S7 Table). The other significant migration links were recognized from Europe to Asian countries, and interconnected among various Asian countries while weaker migration support from North America to South America, and South America to Vietnam were observed (BF>3) (Fig 7 and S7 Table).

**Fig 8:**
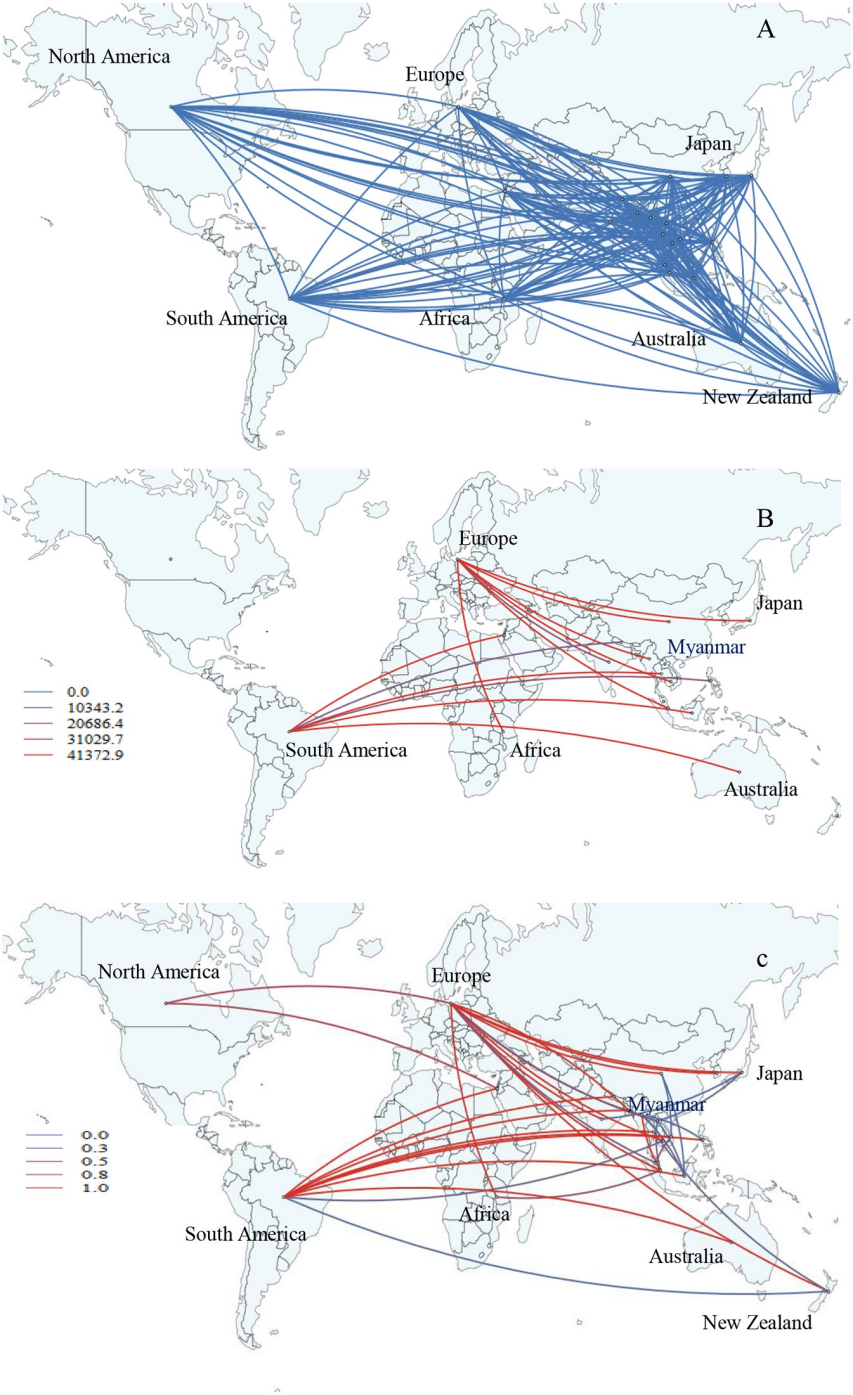
Global migration pathways of influenza B viruses. (A) Spread of influenza B viruses from one location to another between 2010 and 2015. Blue lines indicate the connection between different locations, which represent branches in the MCC tree. (B) The color gradients (from blue to red) of the lines indicate the relative strength of connection between locations according to the Bayes Factor (BF) test. Only rates supported by BF values greater than 1000 are indicated. (C) The color gradients (from blue to red) of the lines indicate the posterior probability of viral migration among locations. Lines were displayed when the posterior probability values were above 0.3.

A marked contrast was observed in the migration pathway influenza B virus when compared to A(H1N1)pdm09 and A(H3H2) strains. Myanmar has strong diffusion rate support for influenza B viral transmission (S6 Fig and S3 Movie). Our data revealed two significant pathways of influenza B virus migration with decisive support (BF>1000) indicating migration from Myanmar to Europe (BF = 41373, posterior probability = 1) and very strong supported pathway (10<BF<1000) with migration from Singapore to Myanmar (BF = 948, posterior probability = 0.98) (Fig 8 and S7 Table). In our study, statistically strong migration links to Europe from Asian countries (e.g. China, Japan, Singapore, and India), Africa, New Zealand and North America were observed (BF>3) (Fig 8 and S7 Table). Other statistically strong migration links were found from South America to Asia (e.g. Indonesia, Thailand, and Philippines) and Australia (BF>3) (Fig 8 and S7 Table). In addition, various inter-continental and multi-country epidemiological links were observed (Fig 8 and S7 Table). The strong statistical support (BF values and posterior probability) may indicate that Myanmar and Asia may play a key role in the seeding of influenza B virus and its spread population to Europe, Africa and South America. We did not find any influenza B strains that persisted more than one year in Myanmar.

## Discussion

We observed the epidemiology of influenza in Myanmar and examined spatial and temporal spread of influenza using HA sequences from our study and global strains during 2010–2015. Increasing evidence shows Asia is the main source of evolution of influenza viruses and the region is important for the management and the prevention strategies against influenza [10]. Our regional phylogenetic analysis supports a strong connection between Myanmar and Asian countries but the global analysis reveals that Myanmar sequences are closely related to sequences from Europe but less with North America and Oceania. According to the Tourism Statistics from the Ministry of Tourism in Myanmar, roughly 1,000,000 to 5,000,000 foreigners in a year visited Myanmar during 2010 and 2015 (http://tourism.gov.mm/en_US/publications/myanmar-tourism-statistics/). The most frequent origins of visitors to Myanmar are Asian countries (60–70%), such as Thailand, China, Japan, and Korea and Malaysia. Accordingly, Thailand, Malaysia, and Bangladesh were the top three countries of migration for Myanmar citizens in 2013 sourced from UNICEF (https://esa.un.org/MigGMGProfiles/indicators/files/Myanmar.pdf). Outside Asia, there were more visitors from Europe (roughly 20%) rather than travelers from North America (6–7%) and Oceania (3%). The diffusion pattern of Myanmar influenza in this study that showed strong connections to Europe as well as Asian countries may be related to the destination of the travelers visiting Myanmar.

Our data showed that an influenza epidemic peaks during the rainy season in Myanmar as reported by our past studies [5, 11]. This is similar to neighboring Southeast Asian countries such as Thailand, Laos, Cambodia, Bangladesh, and Southern China where influenza circulates during July to September in the rainy season, in contrast to Singapore and Indonesia, which are situated at the Equator, where influenza circulates throughout the year without prominent peaks [25, 26].

In this study, we observed influenza activity in three locations in Myanmar and found the seasonality and subtype of influenza A and lineage of influenza B are synchronized among locations in a year. In general, three locations have similar climates with rainy season in May–November, and dry season in December to April with monsoon tropical weather. Pyin Oo Lwin has cooler climate than the other two cities but this time we could just survey for one year. Thus, the difference of influenza seasonality in Pyin Oo Lwin could not be observed. Influenza is transmitted from human to human, so the synchronicity in Myanmar may suggest frequent domestic travel inside the nation. Indeed, Australia observed similar synchronicity of circulating influenza strains in the country despite wide variety of climates, possibly owing to frequent domestic air travels [27].

Our phylogeographic analysis showed that the Myanmar A(H1N1)pdm09 strain in the post-pandemic period originated from Europe and disseminated to various countries including Myanmar. Spatial diffusion pathway analysis showed that Japan played an important role as a hub in our analysis. The migration occurred from Europe, Asia, North America and Oceania to Japan, and Japan to Myanmar and various countries worldwide. Previous study has shown that after the emergence of A(H1N1)pdm09 virus in North America in 2009, the spatial transmission focus was heavily centered in Europe, Southeast Asia and South Asia during 2010–2014, which were similar to our findings [23]. Previous studies done in Brazil, 2009–2014 revealed that new influenza A(H1N1)pdm09 lineages are seeded in each year and the main sources of viral diversity are North America, Europe and East Asia [28]. Vietnam connected strongly with the United States but weakly with other Asian countries [29]. To date, there are no reports to support our finding that Japan is the center for migration of A(H1N1)pdm09 viruses. This might be due to the bias from selection of our data set that was focused on Myanmar and neighboring Asian strains. Japan is among the top three countries of incoming travelers’ origin to Myanmar. The different destinations of inbound and outbound travelers may have caused the different spatial patterns of influenza even among Asian countries.

Bayesian phylogenetic analysis of Myanmar A(H3N2) strains over time showed that Myanmar A(H3N2) viruses from 2010–2015 shared a common ancestor with European strains. Our diffusion pathway analysis indicated that Australia is a significant hub of dissemination for A(H3N2) just as Japan is to A(H1N1)pdm09. A global scale study suggested that the spatio-temporal patterns of A(H3N2) viruses during 2000–2012 revealed that China, India and Southeast Asia acted as the source population for epidemics worldwide [10]. Our findings did not match with their results. Previous study of phylogeographic analysis of A(H3N2) done in Taiwan reported that the 2007 and 2009 summer epidemic originated from Europe but strong connections were seen with North America and Korea [30]. Similarly, during 2001–2008, the subtype A(H3N2) data of Vietnam showed strong migratory connection with neighboring Asian countries and North America which is different from our data [29]. Phylogeographic analysis of A(H3N2) influenza viruses from southern China over a period of 15 years proposed that novel influenza strains emerge and evolve in multiple geographic localities, such as Northern China, North America, Eastern Asia, Europe, and Africa rather than originating from one epicenter such as southern China [26]. However, we cannot explain why Australia with lesser number of reported incoming travelers could be a dissemination center in our study. An alternative explanation would be the timing of travelers from Australia, which may coincide with influenza activity in the country of origin. Increasing evidence shows the significant role of Asia as a dissemination source of A(H3N2), where the annual A(H3N2) influenza epidemics in tropical Asian regions might be influenced by viral input from temperate region in Northern and Southern hemisphere [30].

Our data suggests that Myanmar is a major hub in the influenza B virus transmission network in Asia and has a strong connection of influenza B migration between neighboring Asian countries. The viral migration from Asian countries to Europe and South America to Asian countries were observed during 2010–2015. Previous phylogeographic analyses from 2000 to 2012 revealed that influenza B Victoria and Yamagata lineage viruses in East and Southeast Asia circulate exclusively within this region with limited spreading to other regions for several years [10, 31]. In contrast, phylogeographic analyses revealed that these lineages circulated frequently outside East-Southeast Asia for several years without any evidence of seeding from East-Southeast Asia [10]. We did not observe persistence of influenza B in Myanmar more than one year. To date, there are only few studies on phylogeographic analysis of influenza B [26], highlighting the need for continued monitoring of influenza in the region.

Our study has several limitations. First, clinical samples were collected only in three sites. At the time of this study period, there was no established nationwide surveillance system in Myanmar. In 2017, Myanmar launched a robust national influenza surveillance organized by the Ministry of Health and Sports, with the support from WHO. Seven cities are selected as sentinel surveillance points and 2000 samples are planned to be collected annually. Nonetheless, our data brings important baseline information on influenza epidemiology in Myanmar before the establishment of national surveillance. Second, some countries have submitted more sequences in the database which may lead to oversampling, while other countries have fewer sequences, leading to under-sampling.

## Conclusion

Our study showed the transmission of influenza viruses into and out of Myanmar, which is a part of the complex migration network in Asia, Oceania and Europe. Interestingly, Myanmar plays an important role in reseeding of influenza B epidemic in region. Continuous monitoring and characterization of influenza in Myanmar and countries around the world is needed to fully understand the circulation patterns and origins of influenza in this region. Data on influenza viruses from this region including our study will allow for more comprehensive phylogeographic studies that aim to better understand the global routes of influenza migration.

## Supporting information

S1 Table Primers and probes used for detection of influenza B isolates. (DOCX)

S2 Table Demographic and clinical data and accession numbers of sequences generated in this study. (DOCX)

S3 Table The region and collection time of sequences involved in phylogeographic analysis. (DOCX)

S4 Table Yearly distribution of samples and Influenza virus isolates in Yangon, Myanmar, 2010–2015. (DOCX)

S5 Table Yearly distribution of samples and Influenza virus isolates in Pyinmana, Myanmar, 2010–2015. (DOCX)

S6 Table Yearly distribution of samples and Influenza virus isolates in Pyin Oo Lwin, Myanmar, 2010–2015. (DOCX)

S7 Table Bayes factor and Posterior Probability of migration events of influenza viruses A(H1N1)pdm09, A(H3N2), and B, Myanmar and other countries, 2010–2015. (DOCX)

S1 Fig Regression of root-to-tip genetic distance against sampling date of A(H1N1)pdm09-HA gene. The coefficient correlation of 0.87 and R^2^ of 0.75 show a positive correction between genetic divergence and sampling time. The data set of A(H1N1)pdm09-HA gene is therefore fit for phylogenetic molecular clock analysis in BEAST.
(TIF)

S2 Fig Regression of root-to-tip genetic distance against sampling date of A(H3N2)-HA gene. The coefficient correlation of 0.88 and R^2^ of 0.77 show a positive correction between genetic divergence and sampling time. The data set of A(H3N2)-HA gene is therefore fit for phylogenetic molecular clock analysis in BEAST.
(TIF)

S3 Fig Regression of root-to-tip genetic distance against sampling date of influenza B-HA gene. The coefficient correlation of 0.70 and R^2^ of 0.49 show a positive correction between genetic divergence and sampling time. The data set of influenza B-HA gene is therefore fit for gene phylogenetic molecular clock analysis in BEAST.
(TIF)

S4 Fig Spatio-temporal dispersion pattern of HA of influenza A(H1N1)pdm09 in Asia, Oceania, Europe, North America South America, and Africa. The snapshots show the dispersal pattern of A(H1N1)pdm09 virus of HA from 2010 to 2015. Connections between different countries represent branches in the MCC tree along which the relevant location transition occurs. Location circle diameters are proportional to square root of the number of MCC branches maintaining a particular location state at each time point. The blue gradients show the relative age of transitions for HA. This map is produced by satellite pictures made available in Google Earth.
(TIF)

S5 Fig Spatio-temporal dispersion pattern of HA of influenza A (H3N2) in Asia, Oceania, Europe, North America South America, and Africa. The snapshots show the dispersal pattern of A(H3N2) virus of HA from 2010 to 2015. Connections between different countries represent branches in the MCC tree along which the relevant location transition occurs. Location circle diameters are proportional to square root of the number of MCC branches maintaining a particular location state at each time point. The blue color gradients show the relative age of transitions for HA. This map is produced by satellite pictures made available in Google Earth.
(TIF)

S6 Fig Spatio-temporal dispersion pattern of HA of influenza B in Asia, Oceania, Europe, North America South America, and Africa. The snapshots show the dispersal pattern of influenza B virus of HA from 2010 to 2015. Connections between different countries represent branches in the MCC tree along which the relevant location transition occurs. Location circle diameters are proportional to square root of the number of MCC branches maintaining a particular location state at each time point. The blue color gradients show the relative age of transitions for HA. This map is produced by satellite pictures made available in Google Earth.
(TIF)

S1 Movie Animation of the phylogeographic dispersion pattern of HA of influenza A(H1N1)pdm09 in Asia, Oceania, Europe, North America, South America, and Africa. The dispersal pattern of A(H1N1)pdm09 virus of HA from 2010 to 2015 were analyzed and visualized with the spatial phylogenetic reconstruction of evolutionary dynamics using data-driven documents (SpreaD3) v0.9.7.1. Connections between different countries represent branches in the MCC tree along which the relevant location transition occurs. This map is produced by satellite pictures made available in Google Earth.
(MP4)

S2 Movie Animation of the phylogeographic dispersion pattern of HA of influenza A(H3N2) in Asia, Oceania, Europe, North America, South America, and Africa. The dispersal pattern of A(H3N2) virus of HA from 2010 to 2015 were analyzed and visualized with the spatial phylogenetic reconstruction of evolutionary dynamics using data-driven documents (SpreaD3) v0.9.7.1. Connections between different countries represent branches in the MCC tree along which the relevant location transition occurs. This map is produced by satellite pictures made available in Google Earth.
(MP4)

S3 Movie Animation of the phylogeographic dispersion pattern of HA of influenza B in Asia, Oceania, Europe, North America, South America, and Africa. The dispersal pattern of B virus of HA from 2010 to 2015 were analyzed and visualized with the spatial phylogenetic reconstruction of evolutionary dynamics using data-driven documents (SpreaD3) v0.9.7.1. Connections between different countries represent branches in the MCC tree along which the relevant location transition occurs. This map is produced by satellite pictures made available in Google Earth.
(MP4)
