# Supplementary material for: Phylogeographic analysis of human influenza A and B viruses in Myanmar, 2010–2015
Source: PLoS One. 2019 Jan 10;14(1):e0210550. doi: 10.1371/journal.pone.0210550 (PMC6328249; doi:10.1371/journal.pone.0210550)
Supplement: S1 Table — (DOCX) [file pone.0210550.s001.docx]

S1 Table. Primers and probes used for detection of influenza B isolates

| subtype | Primers and probes | Sequence (5'-3') | Product size (bp) |
| --- | --- | --- | --- |
| FluB/HA | Forward primer(common) | 5’-CCTATAATGCACGACAGAAC-3’ |  |
|  | Victoria reverse primer | 5'-GTTAGGGCAAGACCCTGA-3' | 150 |
|  | Yamagata reverse primer | 5'-GTTAGGGCAAGACCCTGA-3' | 150 |
|  | Victoria FAM probe | 5'-(Eclipse)-CAA(A)ATTGGAACC-(FAM)-3' |  |
|  | Yamagata ROX probe | 5'-(Eclipse)-CAG(A)CTTGGAAC-(ROX)-3' |  |
| FluB/NA | Forward primer(common) | GGTCCGCATGCCAGATG |  |
|  | Victoria reverse primer | GCCATCAGTTATCATAAGA | 193 |
|  | Yamagata reverse primer | TTGCAGGCACTTTCTTGTG | 153 |
|  | Victoria FAM probe | (Eclipse)-TATGTGTCAGT(G)TA-(FAM) |  |
|  | Yamagata ROX probe | (Eclipse)-TGA(G)CAATGCATTA-(ROX) |  |
