# Supplementary material for: Phylogeographic analysis of human influenza A and B viruses in Myanmar, 2010–2015
Source: PLoS One. 2019 Jan 10;14(1):e0210550. doi: 10.1371/journal.pone.0210550 (PMC6328249; doi:10.1371/journal.pone.0210550)
Supplement: S2 Table — (DOCX) [file pone.0210550.s002.docx]

S2 Table. Demographic and clinical data and accession numbers of sequences generated in this study

| Name of strain | Age(Yr) | Gender | Isolation source | Site of sample collection | Collection month and year | Accession number |
| --- | --- | --- | --- | --- | --- | --- |
|  |  |  |  |  |  | GISAID |
|  |  |  |  |  |  | HA |
| Influenza A(H1N1pdm09) | |  |  |  |  |  |
| A/Myanmar/10M005/2010 | 9 | F | Nasopharyngeal | Yangon | May, 2010 | EPI1002260 |
| A/Myanmar/10M048/2010 | 1.5 | M | Nasopharyngeal | Yangon | June, 2010 | EPI1002262 |
| A/Myanmar/10M287/2010 | 2.25 | F | Nasopharyngeal | Yangon | July, 2010 | EPI1002264 |
| A/Myanmar/10M370/2010 | 1.5 | F | Nasopharyngeal | Yangon | January, 2010 | EPI1002266 |
| A/Myanmar/10M408/2010 | 6 | M | Nasopharyngeal | Yangon | August, 2010 | EPI1002268 |
| A/Myanmar/10M425/2010 | 0.5 | M | Nasopharyngeal | Yangon | August, 2010 | EPI1002270 |
| A/Myanmar/10M456/2010 | 2 | M | Nasopharyngeal | Pyinmana | July, 2010 | EPI1002272 |
| A/Myanmar/10M476/2010 | 30 | F | Nasopharyngeal | Pyinmana | July, 2010 | EPI1002274 |
| A/Myanmar/10M499/2010 | 13 | F | Nasopharyngeal | Pyinmana | July, 2010 | EPI1002276 |
| A/Myanmar/10M505/2010 | 34 | F | Nasopharyngeal | Pyinmana | August, 2010 | EPI1002278 |
| A/Myanmar/10M539/2010 | 8 | F | Nasopharyngeal | Pyinmana | August, 2010 | EPI1002280 |
| A/Myanmar/10M542/2010 | 5 | M | Nasopharyngeal | Pyinmana | August, 2010 | EPI1002282 |
| A/Myanmar/10M558/2010 | 30 | M | Nasopharyngeal | Pyinmana | September, 2010 | EPI1002284 |
| A/Myanmar/10M577/2010 | 20 | M | Nasopharyngeal | Yangon | September, 2010 | EPI1002286 |
| A/Myanmar/12M003/2012 | 4 | M | Nasopharyngeal | Yangon | June, 2012 | EPI1002610 |
| A/Myanmar/12M010/2012 | 4 | F | Nasopharyngeal | Yangon | June, 2012 | EPI1002646 |
| A/Myanmar/12M011/2012 | 0.5 | M | Nasopharyngeal | Yangon | June, 2012 | EPI1002653 |
| A/Myanmar/12M012/2012 | 6 | M | Nasopharyngeal | Yangon | June, 2012 | EPI1002655 |
| A/Myanmar/12M013/2012 | 5 | M | Nasopharyngeal | Yangon | June, 2012 | EPI1002657 |
| A/Myanmar/12M021/2012 | 5 | F | Nasopharyngeal | Yangon | June, 2012 | EPI1002659 |
| A/Myanmar/12M126/2012 | 7 | M | Nasopharyngeal | Yangon | June, 2012 | EPI1002667 |
| A/Myanmar/13M310/2013 | 6 | F | Nasopharyngeal | Pyinmana | September, 2013 | EPI562616 |
| A/Myanmar/14M003/2014 | 1.5 | F | Nasopharyngeal | Yangon | June, 2014 | EPI567953 |
| A/Myanmar/14M072/2014 | 3.6 | F | Nasopharyngeal | Yangon | July, 2014 | EPI567961 |
| A/Myanmar/14M139/2014 | 1 | F | Nasopharyngeal | Yangon | August, 2014 | EPI567982 |
| A/Myanmar/14M233/2014 | 21 | M | Nasopharyngeal | Pyinmana | August, 2014 | EPI568020 |
| A/Myanmar/14M272/2014 | 12 | M | Nasopharyngeal | Pyinmana | July, 2014 | EPI568028 |
| A/Myanmar/14M379/2014 | 20 | M | Nasopharyngeal | Pyin Oo Lwin | August, 2014 | EPI568036 |
| A/Myanmar/14M445/2014 | 38 | M | Nasopharyngeal | Pyin Oo Lwin | September, 2014 | EPI568044 |
| A/Myanmar/14M194/2014 | 38 | F | Nasopharyngeal | Pyinmana | July, 2014 | EPI568012 |
| A/Myanmar/15M012/2015 | 0.7 | F | nasal swab | Yangon | August, 2015 | EPI1002669 |
| A/Myanmar/15M008/2015 | 3 | F | nasal swab | Yangon | July, 2015 | EPI1002671 |
| A/Myanmar/15M034/2015 | 4.5 | F | nasal swab | Yangon | August, 2015 | EPI1002673 |
| A/Myanmar/15M003/2015 | 8 | M | nasal swab | Yangon | July, 2015 | EPI1002675 |
| A/Myanmar/15M037/2015 | 5 | M | nasal swab | Yangon | August, 2015 | EPI1002677 |
| A/Myanmar/15M108/2015 | 2.6 | M | nasal swab | Yangon | August, 2015 | EPI1002679 |
| A/Myanmar/15M117/2015 | 7 | M | nasal swab | Pyinmana | July, 2015 | EPI1002681 |
| A/Myanmar/15M123/2015 | 0.6 | M | nasal swab | Pyinmana | July, 2015 | EPI1002683 |
| A/Myanmar/15M137/2015 | 27 | M | nasal swab | Pyinmana | July, 2015 | EPI1002685 |
| A/Myanmar/15M150/2015 | 63 | M | nasal swab | Pyinmana | August, 2015 | EPI1002687 |
| A/Myanmar/15M161/2015 | 5 | F | nasal swab | Pyinmana | August, 2015 | EPI1002689 |
| A/Myanmar/15M173/2015 | 4 | M | nasal swab | Pyinmana | August, 2015 | EPI1002691 |
| A/Myanmar/15M205/2015 | 13 | F | nasal swab | Pyinmana | September, 2015 | EPI1002693 |
| Influenza A(H3N2) |  |  |  |  |  |  |
| A/Myanmar/10M125/2010 | 0.75 | M | nasopharyngeal | Yangon | July, 2010 | EPI1002701 |
| A/Myanmar/10M365/2010 | 2 | F | nasopharyngeal | Yangon | August, 2010 | EPI1002703 |
| A/Myanmar/10M373/2010 | 2 | M | nasopharyngeal | Yangon | August, 2010 | EPI1002705 |
| A/Myanmar/10M379/2010 | 2 | F | nasopharyngeal | Yangon | August, 2010 | EPI1002707 |
| A/Myanmar/10M409/2010 | 1.75 | F | nasopharyngeal | Yangon | August, 2010 | EPI1002709 |
| A/Myanmar/10M410/2010 | 3 | F | nasopharyngeal | Yangon | August, 2010 | EPI1002711 |
| A/Myanmar/10M417/2010 | 3 | F | nasopharyngeal | Yangon | August, 2010 | EPI1002713 |
| A/Myanmar/10M574/2010 | 2.5 | F | nasopharyngeal | Pyinmana | September, 2010 | EPI1002715 |
| A/Myanmar/11M019/2011 | 1.1 | F | nasopharyngeal | Yangon | June, 2011 | EPI1002717 |
| A/Myanmar/11M020/2011 | 4 | F | nasopharyngeal | Yangon | June, 2011 | EPI1002719 |
| A/Myanmar/11M176/2011 | 1.3 | F | nasopharyngeal | Yangon | July, 2011 | EPI1002721 |
| A/Myanmar/11M222/2011 | 1.5 | M | nasopharyngeal | Yangon | November, 2011 | EPI1002723 |
| A/Myanmar/11M252/2011 | 1 | F | nasopharyngeal | Yangon | August, 2011 | EPI1004069 |
| A/Myanmar/11M280/2011 | 5 | F | nasopharyngeal | Yangon | August, 2011 | EPI1004035 |
| A/Myanmar/11M286/2011 | 3 | F | nasopharyngeal | Yangon | September, 2011 | EPI1004037 |
| A/Myanmar/11M290/2011 | 25 | F | nasopharyngeal | Pyinmana | June, 2011 | EPI1004039 |
| A/Myanmar/11M292/2011 | 11 | F | nasopharyngeal | Pyinmana | July, 2011 | EPI1004041 |
| A/Myanmar/11M406/2011 | 2 | F | nasopharyngeal | Pyinmana | August, 2011 | EPI1004043 |
| A/Myanmar/11M412/2011 | 58 | F | nasopharyngeal | Pyinmana | September, 2011 | EPI1004045 |
| A/Myanmar/13M006/2013 | 2 | M | nasopharyngeal | Yangon | April, 2013 | EPI566194 |
| A/Myanmar/13M020/2013 | 1.5 | M | nasopharyngeal | Yangon | November, 2013 | EPI566210 |
| A/Myanmar/13M035/2013 | 6 | F | nasopharyngeal | Yangon | July, 2013 | EPI566218 |
| A/Myanmar/13M044/2013 | 3 .5 | M | nasopharyngeal | Yangon | July, 2013 | EPI566527 |
| A/Myanmar/13M061/2013 | 6 | F | nasopharyngeal | Yangon | July, 2013 | EPI566535 |
| A/Myanmar/13M062/2013 | 0.75 | M | nasopharyngeal | Yangon | July, 2013 | EPI566543 |
| A/Myanmar/13M070/2013 | 1 | F | nasopharyngeal | Yangon | July, 2013 | EPI566615 |
| A/Myanmar/13M076/2013 | 2 | M | nasopharyngeal | Yangon | July, 2013 | EPI566623 |
| A/Myanmar/13M084/2013 | 4.5 | F | nasopharyngeal | Yangon | July, 2013 | EPI566631 |
| A/Myanmar/13M087/2013 | 4.5 | M | nasopharyngeal | Yangon | July, 2013 | EPI566673 |
| A/Myanmar/13M088/2013 | 7 | F | nasopharyngeal | Yangon | July, 2013 | EPI566681 |
| A/Myanmar/13M089/2013 | 1.5 | M | nasopharyngeal | Yangon | July, 2013 | EPI566707 |
| A/Myanmar/13M102/2013 | 2.5 | M | nasopharyngeal | Yangon | July, 2013 | EPI566723 |
| A/Myanmar/13M124/2013 | 6 | F | nasopharyngeal | Yangon | July, 2013 | EPI566755 |
| A/Myanmar/13M300/2013 | 77 | M | nasopharyngeal | Pyinmana | August, 2013 | EPI566763 |
| A/Myanmar/14M039/2014 | 6 | M | nasopharyngeal | Yangon | June, 2014 | EPI568050 |
| A/Myanmar/14M059/2014 | 12 | F | nasopharyngeal | Yangon | July, 2014 | EPI568058 |
| A/Myanmar/14M122/2014 | 0.83 | M | nasopharyngeal | Yangon | July, 2014 | EPI568066 |
| A/Myanmar/14M187/2014 | 26 | F | nasopharyngeal | Pyinmana | July, 2014 | EPI568074 |
| A/Myanmar/14M240/2014 | 6 | F | nasopharyngeal | Pyinmana | August, 2014 | EPI568082 |
| A/Myanmar/14M391/2014 | 4 | M | nasopharyngeal | Pyinmana | August, 2014 | EPI568090 |
| A/Myanmar/14M465/2014 | 20 | M | nasopharyngeal | Pyin Oo Lwin | September, 2014 | EPI568098 |
| A/Myanmar/15M124/2015 | 5 | F | nasal swab | Pyinmana | July, 2015 | EPI1002727 |
| A/Myanmar/15M145/2015 | 1 .58 | M | nasal swab | Pyinmana | August, 2015 | EPI1002729 |
| A/Myanmar/15M154/2015 | 13 | M | nasal swab | Pyinmana | August, 2015 | EPI1002731 |
| A/Myanmar/15M162/2015 | 14 | F | nasal swab | Pyinmana | August, 2015 | EPI1002733 |
| A/Myanmar/15M178/2015 | 1.16 | F | nasal swab | Pyinmana | August, 2015 | EPI1004071 |
| A/Myanmar/15M183/2015 | 8 | M | nasal swab | Pyinmana | August, 2015 | EPI1002737 |
| A/Myanmar/15M193/2015 | 15 | F | nasal swab | Pyinmana | August, 2015 | EPI1002739 |
| A/Myanmar/15M203/2015 | 15 | M | nasal swab | Pyinmana | September, 2015 | EPI1002741 |
| A/Myanmar/15M208/2015 | 16 | M | nasal swab | Pyinmana | September, 2015 | EPI1002743 |
| Influenza B |  |  |  |  |  |  |
| B/Myanmar/10M114/2010 | 2.5 | M | nasopharyngeal | Yangon | June, 2010 | EPI1002745 |
| B/Myanmar/10M295/2010 | 9 | F | nasopharyngeal | Yangon | July, 2010 | EPI1002747 |
| B/Myanmar/10M310/2010 | 7 | M | nasopharyngeal | Yangon | July, 2010 | EPI1002749 |
| B/Myanmar/10M320/2010 | 1.75 | F | nasopharyngeal | Yangon | July, 2010 | EPI1002751 |
| B/Myanmar/10M342/2010 | 2.5 | F | nasopharyngeal | Yangon | August, 2010 | EPI1002753 |
| B/Myanmar/10M389/2010 | 7 | F | nasopharyngeal | Yangon | August, 2010 | EPI1002755 |
| B/Myanmar/10M394/2010 | 6 | F | nasopharyngeal | Yangon | August, 2010 | EPI1002757 |
| B/Myanmar/10M442/2010 | 5.5 | F | nasopharyngeal | Yangon | October, 2010 | EPI1002759 |
| B/Myanmar/10M523/2010 | 21 | F | nasopharyngeal | Pyinmana | August, 2010 | EPI1002761 |
| B/Myanmar/10M556/2010 | 25 | F | nasopharyngeal | Pyinmana | August, 2010 | EPI1002763 |
| B/Myanmar/10M560/2010 | 20 | F | nasopharyngeal | Pyinmana | September, 2010 | EPI1002765 |
| B/Myanmar/10M567/2010 | 11 | M | nasopharyngeal | Pyinmana | September, 2010 | EPI1002767 |
| B/Myanmar/10M575/2010 | 8 | F | nasopharyngeal | Pyinmana | September, 2010 | EPI1002769 |
| B/Myanmar/10M580/2010 | 17 | F | nasopharyngeal | Pyinmana | September, 2010 | EPI1002771 |
| B/Myanmar/10M654/2010 | 11 | F | nasopharyngeal | Pyinmana | September, 2010 | EPI1002773 |
| B/Myanmar/10M659/2010 | 38 | F | nasopharyngeal | Pyinmana | September, 2010 | EPI1002775 |
| B/Myanmar/10M662/2010 | 7 | F | nasopharyngeal | Pyinmana | September, 2010 | EPI1002777 |
| B/Myanmar/10M667/2010 | 8 | F | nasopharyngeal | Pyinmana | October, 2010 | EPI1002779 |
| B/Myanmar/10M676/2010 | 12 | F | nasopharyngeal | Pyinmana | October, 2010 | EPI1002781 |
| B/Myanmar/10M678/2010 | 32 | F | nasopharyngeal | Pyinmana | October, 2010 | EPI1002783 |
| B/Myanmar/10M680/2010 | 32 | F | nasopharyngeal | Pyinmana | October, 2010 | EPI1002785 |
| B/Myanmar/10M692/2010 | 23 | F | nasopharyngeal | Pyinmana | December, 2010 | EPI1002787 |
| B/Myanmar/11M265/2011 | 2.5 | M | nasopharyngeal | Yangon | August, 2011 | EPI1002789 |
| B/Myanmar/11M279/2011 | 0.58 | F | nasopharyngeal | Yangon | August, 2011 | EPI1002791 |
| B/Myanmar/12M396/2012 | 3.5 | M | nasopharyngeal | Pyinmana | July, 2012 | EPI1002793 |
| B/Myanmar/12M444/2012 | 30 | F | nasopharyngeal | Pyinmana | August, 2012 | EPI1002795 |
| B/Myanmar/12M445/2012 | 30 | F | nasopharyngeal | Pyinmana | August, 2012 | EPI1002797 |
| B/Myanmar/12M496/2012 | 3 | M | nasopharyngeal | Pyinmana | August, 2012 | EPI1002799 |
| B/Myanmar/12M508/2012 | 4 | M | nasopharyngeal | Pyinmana | September, 2012 | EPI1002801 |
| B/Myanmar/12M022/2012 | 9 | M | nasopharyngeal | Yangon | June, 2012 | EPI1002803 |
| B/Myanmar/12M373/2012 | 12 | M | nasopharyngeal | Pyinmana | July, 2012 | EPI1004051 |
| B/Myanmar/12M391/2012 | 47 | M | nasopharyngeal | Pyinmana | July, 2012 | EPI1004053 |
| B/Myanmar/12M400/2012 | 9 | F | nasopharyngeal | Pyinmana | July, 2012 | EPI1004056 |
| B/Myanmar/12M413/2012 | 12 | F | nasopharyngeal | Pyinmana | August, 2012 | EPI1004058 |
| B/Myanmar/12M462/2012 | 4.6 | M | nasopharyngeal | Pyinmana | August, 2012 | EPI1004060 |
| B/Myanmar/12M481/2012 | 4 | F | nasopharyngeal | Pyinmana | August, 2012 | EPI1004062 |
| B/Myanmar/12M507/2012 | 1.25 | M | nasopharyngeal | Pyinmana | September, 2012 | EPI1004064 |
| B/Myanmar/12M517/2012 | 12 | F | nasopharyngeal | Pyinmana | September, 2012 | EPI1004066 |
| B/Myanmar/14M001/2014 | 5 | M | nasopharyngeal | Yangon | April, 2014 | EPI568109 |
| B/Myanmar/14M101/2014 | 8 | F | nasopharyngeal | Yangon | July, 2014 | EPI568117 |
| B/Myanmar/14M138/2014 | 6 | F | nasopharyngeal | Yangon | August, 2014 | EPI568125 |
| B/Myanmar/14M225/2014 | 47 | F | nasopharyngeal | Pyinmana | July, 2014 | EPI568133 |
| B/Myanmar/14M238/2014 | 45 | F | nasopharyngeal | Pyinmana | August, 2014 | EPI568141 |
| B/Myanmar/14M297/2014 | 15 | F | nasopharyngeal | Pyinmana | July, 2014 | EPI568149 |
| B/Myanmar/14M408/2014 | 32 | F | nasopharyngeal | Pyin Oo Lwin | August, 2014 | EPI568157 |
| B/Myanmar/14M456/2014 | 11 | M | nasopharyngeal | Pyin Oo Lwin | September, 2014 | EPI568165 |
