# Supplementary material for: Phylogeographic analysis of human influenza A and B viruses in Myanmar, 2010–2015
Source: PLoS One. 2019 Jan 10;14(1):e0210550. doi: 10.1371/journal.pone.0210550 (PMC6328249; doi:10.1371/journal.pone.0210550)
Supplement: S3 Table — (DOCX) [file pone.0210550.s003.docx]

S3 Table. The region and collection time of sequences involved in phylogeographic analysis

| Strains | Country | Collection month and year | Accession number |
| --- | --- | --- | --- |
| A(H1N1)pdm09 |  |  |  |
| A/Nepal/165/2010 | Nepal | September, 2010 | EPI346867 |
| A/Nepal/01548/2012 | Nepal | September, 2012 | EPI485910 |
| A/Nepal/0469/2013 | Nepal | August, 2013 | EPI544192 |
| A/Nepal/0357/2014 | Nepal | March, 2014 | EPI544200 |
| A/Nepal/3594/2015 | Nepal | December, 2015 | EPI849522 |
| A/Afghanistan/0082/2015 | Afghanistan | December, 2015 | EPI732950 |
| A/Afghanistan/654/2015 | Afghanistan | December, 2015 | EPI827949 |
| A/Bangladesh/1007/2012 | Bangladesh | April, 2012 | EPI418260 |
| A/Bangladesh/8002/2010 | Bangladesh | July, 2010 | EPI295432 |
| A/Bangladesh/2005/2010 | Bangladesh | June, 2010 | EPI295435 |
| A/Bangladesh/1685/2010 | Bangladesh | September, 2010 | EPI295501 |
| A/Bangladesh/8003/2010 | Bangladesh | September, 2010 | EPI300987 |
| A/Bangladesh/5513/2011 | Bangladesh | February, 2011 | EPI309812 |
| A/Bangladesh/8324/2011 | Bangladesh | May, 2011 | EPI33120 |
| A/Bangladesh/9751/2011 | Bangladesh | July, 2011 | EPI335789 |
| A/Bangladesh/2110/2011 | Bangladesh | October, 2011 | EPI348169 |
| A/Bangladesh/3082/2012 | Bangladesh | February, 2012 | EPI376401 |
| A/Bangladesh/2014/2012 | Bangladesh | March, 2012 | EPI386044 |
| A/Bangladesh/2249/2012 | Bangladesh | July, 2012 | EPI397698 |
| A/Bangladesh/6382/2013 | Bangladesh | January, 2013 | EPI442691 |
| A/Bangladesh/8207/2013 | Bangladesh | May, 2013 | EPI465067 |
| A/Bangladesh/2002/2013 | Bangladesh | April, 2013 | EPI465073 |
| A/Bangladesh/3003/2013 | Bangladesh | August, 2013 | EPI485766 |
| A/Bangladesh/0009/2014 | Bangladesh | March, 2014 | EPI531881 |
| A/Bangladesh/8027/2014 | Bangladesh | March, 2014 | EPI531917 |
| A/Bangladesh/4001/2015 | Bangladesh | February, 2015 | EPI587742 |
| A/Bangladesh/2009/2015 | Bangladesh | March, 2015 | EPI675017 |
| A/Bangladesh/5381/2015 | Bangladesh | April, 2015 | EPI704124 |
| A/Bangladesh/05/2015 | Bangladesh | May, 2015 | EPI629081 |
| A/Bangladesh/861/2015 | Bangladesh | May, 2015 | EPI643431 |
| A/Cambodia/10/2010 | Cambodia | July, 2010 | EPI279150 |
| A/CAMBODIA/7/2010 | Cambodia | July, 2010 | EPI279141 |
| A/CAMBODIA/8/2010 | Cambodia | July, 2010 | EPI279144 |
| A/Cambodia/V0601312/2011 | Cambodia | May, 2011 | EPI447019 |
| A/Cambodia/V0902314/2011 | Cambodia | August, 2011 | EPI447022 |
| A/Cambodia/V1019320/2011 | Cambodia | October, 2011 | EPI447024 |
| A/Cambodia/1300/2015 | Cambodia | December, 2015 | EPI759336 |
| A/Cambodia/1340/2015 | Cambodia | December, 2015 | EPI759352 |
| A/Cambodia/1191/2015 | Cambodia | November, 2015 | EPI762479 |
| A/Cambodia/W1023346/2012 | Cambodia | August, 2012 | EPI873023 |
| A/Cambodia/W1023356/2012 | Cambodia | September, 2012 | EPI873025 |
| A/Cambodia/W1130333/2012 | Cambodia | November, 2012 | EPI873211 |
| A/Cambodia/X0918311/2013 | Cambodia | September, 2013 | EPI873233 |
| A/Cambodia/X1104331/2013 | Cambodia | October, 2013 | EPI873303 |
| A/Cambodia/X1126400/2013 | Cambodia | November, 2013 | EPI873537 |
| A/Cambodia/Y0407303/2014 | Cambodia | April, 2014 | EPI873539 |
| A/Cambodia/Y0630301/2014 | Cambodia | September, 2014 | EPI873542 |
| A/Cambodia/Y0721351/2014 | Cambodia | April, 2014 | EPI873547 |
| A/Hong Kong/2172/2010 | China | July, 2010 | EPI280298 |
| A/Hong_Kong/5659/2012 | China | May, 2012 | EPI390473 |
| A/Beijing/HZ01/2011 | China | January, 2011 | EPI317117 |
| A/FuZhou/SWL1124/2013 | China | February, 2013 | AIW63617 |
| A/FuZhou/SWL172/2010 | China | January, 2010 | AIW63580 |
| A/FuZhou/SWL1962/2012 | China | December, 2012 | AIW63607 |
| A/Guangdong/002/2011 | China | January, 2011 | EPI354465 |
| A/Guangdong/1513/2012 | China | December, 2012 | AGG20153 |
| A/Guangdong/2989/2010 | China | March, 2010 | EPI354483 |
| A/XiaMen/SWL1272/2014 | China | April, 2014 | AIW63671 |
| A/XiaMen/SWL135/2013 | China | January, 2013 | AIW63615 |
| A/Zhejiang/HuZ1/2012 | China | December, 2012 | EPI426868 |
| A/Jiangsusucheng/SWL1148/2013 | China | February, 2013 | AIZ07421 |
| A/LongYan/SWL280/2014 | China | February, 2014 | AIW63664 |
| A/Shanghai/2167T/2010 | China | January, 2010 | AEQ19882 |
| A/Shanghai/6109T/2014 | China | January, 2014 | AIT93147 |
| A/Sichuan-Qingyang/SWL1599/2013 | China | November, 2013 | EPI498564 |
| A/QuanZhou/SWL1135/2013 | China | May, 2013 | AIW63629 |
| A/Taiwan/254/2012 | China | March, 2012 | EPI378241 |
| A/Guangdong-Haizhu/SWL165/2015 | China | January, 2015 | EPI586302 |
| A/India/4051/2015 | India | September, 2015 | EPI772948 |
| A/India/5103/2010 | India | August, 2010 | EPI295459 |
| A/India/5756/2011 | India | October, 2011 | EPI353396 |
| A/India/5964/2014 | India | May, 2014 | EPI536832 |
| A/India/610/2015 | India | March, 2015 | EPI756223 |
| A/India/7710/2012 | India | July, 2012 | EPI394857 |
| A/India/GWL01/2011 | India | August, 2011 | JQ319658 |
| A/India/Nsk12388/2012 | India | January, 2012 | EPI468897 |
| A/India/P1112874/2011 | India | August, 2011 | EPI468881 |
| A/India/P1114854/2011 | India | September, 2011 | EPI468889 |
| A/India/P121716/2012 | India | February, 2012 | EPI468905 |
| A/India/P121778/2012 | India | February, 2012 | EPI468852 |
| A/India/P12946/2012 | India | February, 2012 | EPI468868 |
| A/India/P131027/2013 | India | January, 2013 | EPI498109 |
| A/India/P131845/2013 | India | February, 2013 | EPI468824 |
| A/India/P132194/2013 | India | February, 2013 | EPI468832 |
| A/India/Pun1415432/2014 | India | October, 2014 | EPI743669 |
| A/Bangalore/1155-33/2012 | India | May, 2012 | KR861686 |
| A/India/Pun151192/2015 | India | January, 2015 | EPI743672 |
| A/Ngp/NIV22704/2010 | India | January, 2010 | CY075919 |
| A/Mumbai/4923/2010 | India | July, 2010 | KM219060 |
| A/Vadu/NIV1043725/2010 | India | April, 2010 | CY075889 |
| A/India/Pun151268/2015 | India | January, 2015 | EPI743674 |
| A/India/Pun151399/2015 | India | February, 2015 | EPI743676 |
| A/Indonesia/NIHRDMTR003/2011 | Indonesia | September, 2011 | EPI345956 |
| A/Indonesia/NIHRDI-DPS805/2012 | Indonesia | April, 2012 | EPI414284 |
| A/Indonesia/NIHRDI-PLK0035/2012 | Indonesia | December, 2012 | EPI416316 |
| A/Indonesia/Nihrd-Pdgd_572/2014 | Indonesia | February, 2014 | EPI547613 |
| A/Indonesia/Nihrdi-Lpg_131/2014 | Indonesia | February, 2014 | EPI547617 |
| A/Indonesia/Nihrd-Pal082/2015 | Indonesia | February, 2015 | EPI651815 |
| A/Indonesia/Nihrdi-Diy152/2015 | Indonesia | April, 2015 | EPI668475 |
| A/Indonesia/1385_S92_L001/2013 | Indonesia | February, 2013 | EPI1023895 |
| A/Indonesia/221_S38_L001/2011 | Indonesia | March, 2011 | EPI1025259 |
| A/Indonesia/1366_S56_L001/2013 | Indonesia | February, 2013 | EPI1027905 |
| A/Israel/Q-657/2015 | Israel | December, 2015 | EPI697732 |
| A/Israel/L-1227/2011 | Israel | January, 2011 | EPI319477 |
| A/Israel/11/2012 | Israel | December, 2012 | EPI425983 |
| A/Israel/28/2013 | Israel | January, 2013 | EPI425987 |
| A/Israel/33/2013 | Israel | January, 2013 | EPI505303 |
| A/Israel/P-352/2014 | Israel | December, 2014 | EPI561730 |
| A/Israel/P-412/2014 | Israel | December, 2014 | EPI561732 |
| A/Israel/A-6615/2015 | Israel | December, 2015 | EPI697725 |
| A/Israel/Q-363/2015 | Israel | December, 2015 | EPI717675 |
| A/Lebanon/14L61/2014 | Lebanon | February, 2014 | EPI580459 |
| A/Lebanon/14L62/2014 | Lebanon | February, 2014 | EPI580467 |
| A/Lebanon/14L66/2014 | Lebanon | February, 2014 | EPI580476 |
| A/Laos/1096/2010 | Laos | November, 2010 | EPI309937 |
| A/Laos/71/2010 | Laos | November, 2010 | EPI309940 |
| A/Laos/I650/2012 | Laos | August, 2012 | EPI418038 |
| A/Laos/839/2012 | Laos | November, 2012 | EPI439236 |
| A/Laos/I600/2013 | Laos | July, 2013 | EPI497966 |
| A/Laos/I766/2013 | Laos | August, 2013 | EPI497970 |
| A/Laos/I829/2013 | Laos | September, 2013 | EPI497974 |
| A/Laos/887/2013 | Laos | September, 2013 | EPI516740 |
| A/Laos/828/2013 | Laos | September, 2013 | EPI516326 |
| A/Laos/I450/2014 | Laos | May, 2014 | EPI548522 |
| A/Laos/SA097/2014 | Laos | February, 2014 | EPI548528 |
| A/Laos/SA183/2014 | Laos | March, 2014 | EPI548528 |
| A/Laos/I717/2014 | Laos | July, 2014 | EPI557599 |
| A/Laos/1187/2014 | Laos | November, 2014 | EPI626108 |
| A/Laos/68/2015 | Laos | February, 2015 | EPI626012 |
| A/Laos/78/2015 | Laos | January, 2015 | EPI626116 |
| A/Laos/I281/2015 | Laos | March, 2015 | EPI638838 |
| A/Laos/O230/2015 | Laos | April, 2015 | EPI638846 |
| A/Laos/193/2015 | Laos | May, 2015 | EPI638894 |
| A/Laos/1058/2010 | Laos | October, 2010 | EPI967845 |
| A/Laos/065/2011 | Laos | February, 2011 | EPI967846 |
| A/Malaysia/09707/2015 | Malaysia | August, 2015 | EPI704136 |
| A/MALAYSIA/174/2010 | Malaysia | February, 2010 | EPI294192 |
| A/MALAYSIA/478/2011 | Malaysia | April, 2011 | EPI349175 |
| A/MALAYSIA/783/2012 | Malaysia | July, 2012 | EPI450249 |
| A/MALAYSIA/9/2013 | Malaysia | November, 2013 | EPI529425 |
| A/MALAYSIA/4/2014 | Malaysia | January, 2014 | EPI541198 |
| A/Malaysia/2863/2015 | Malaysia | September, 2015 | EPI765125 |
| A/MALAYSIA/32/2014 | Malaysia | June, 2014 | EPI551389 |
| A/Malaysia/11/2014 | Malaysia | March, 2014 | EPI562720 |
| A/Malaysia/1335/2015 | Malaysia | June, 2015 | EPI636110 |
| A/PHILIPPINES/3334/2010 | Philippines | July, 2010 | EPI331312 |
| A/Philippines/TMC10-41/2010 | Philippines | July, 2010 | EPI336771 |
| A/PHILIPPINES/850/2011 | Philippines | July, 2011 | EPI349258 |
| A/PHILIPPINES/1366/2011 | Philippines | July, 2011 | EPI349309 |
| A/PHILIPPINES/3/2013 | Philippines | March, 2013 | EPI491548 |
| A/PHILIPPINES/12/2014 | Philippines | April, 2014 | EPI551410 |
| A/Philippines/11/2013 | Philippines | August, 2013 | EPI567293 |
| A/Philippines/2638/2010 | Philippines | June, 2010 | EPI294118 |
| A/Philippines/7/2015 | Philippines | March, 2015 | EPI636146 |
| A/Philippines/32/2015 | Philippines | October, 2015 | EPI868922 |
| A/Korea/AF2428/2010 | South Korea | February, 2010 | EPI278218 |
| A/Korea/664/2010 | South Korea | December, 2010 | EPI309934 |
| A/Gyeongnam/3526/2012 | South Korea | December, 2012 | EPI426460 |
| A/Gyeonggibuk/1678/2013 | South Korea | November, 2013 | EPI515729 |
| A/Jeonbuk/2005/2014 | South Korea | December, 2014 | EPI564968 |
| A/Seoul/1596/2015 | South Korea | October, 2015 | EPI716779 |
| A/Singapore/GP1598/2015 | Singapore | August, 2015 | EPI724876 |
| A/Singapore/TT128/2010 | Singapore | March, 2010 | AFP35175 |
| A/SINGAPORE/591/2010 | Singapore | September, 2010 | EPI331342 |
| A/SINGAPORE/7/2011 | Singapore | January, 2011 | EPI331411 |
| A/SINGAPORE/62/2011 | Singapore | November, 2011 | EPI370242 |
| A/SINGAPORE/12/2012 | Singapore | June, 2012 | EPI394292 |
| A/Singapore/TT213/2015 | Singapore | March, 2015 | EPI726975 |
| A/Singapore/48/2012 | Singapore | October, 2012 | EPI748162 |
| A/Singapore/EN290/2014 | Singapore | June, 2014 | EPI748166 |
| A/Singapore/KK457/2013 | Singapore | June, 2013 | EPI748178 |
| A/Singapore/575/2010 | Singapore | May, 2010 | EPI763056 |
| A/Phuket/316/2014 | Thailand | July, 2014 | EPI541524 |
| A/Thailand/594/2010 | Thailand | September, 2010 | EPI295495 |
| A/Thailand/CU_FS2/2014 | Thailand | April, 2014 | ALQ12527 |
| A/Thailand/NHRC430218/2014 | Thailand | February, 2014 | AID48029 |
| A/Thailand/SirirajICRC NMA 15/2011 | Thailand | June, 2011 | AHB59409 |
| A/Thailand/SN11851/2012 | Thailand | November, 2012 | AJJ99675 |
| A/Bangkok/INS3_681/2012 | Thailand | September, 2012 | AHM98778 |
| A/Bangkok/INS424/2010 | Thailand | February, 2010 | ADM13291 |
| A/Bangkok/INS486/2010 | Thailand | August, 2010 | AEN55463 |
| A/Bangkok/SIMI503/2010 | Thailand | August, 2010 | AIE52361 |
| A/SONG KHLA/34/2011 | Thailand | August, 2011 | EPI331546 |
| A/Nonthaburi/78/2011 | Thailand | June, 2011 | EPI347098 |
| A/Song Khla/270/2011 | Thailand | September, 2011 | EPI346513 |
| A/Phuket/01/2013 | Thailand | January, 2013 | EPI552426 |
| A/Bangkok/374/2013 | Thailand | November, 2013 | EPI552430 |
| A/Phuket/138/2013 | Thailand | March, 2013 | EPI584725 |
| A/Nonthaburi/53/2015 | Thailand | June, 2015 | EPI643330 |
| A/Chanthaburi/38/2015 | Thailand | June, 2015 | EPI643494 |
| A/Bangkok/399/2015 | Thailand | October, 2015 | EPI720530 |
| A/Vietnam/21/2010 | Vietnam | January, 2010 | EPI310139 |
| A/Vietnam/57/2010 | Vietnam | March, 2010 | EPI310142 |
| A/Vietnam/108/2011 | Vietnam | April, 2011 | EPI366346 |
| A/Vietnam/3029/2013 | Vietnam | March, 2013 | EPI497640 |
| A/Vietnam/105/2012 | Vietnam | April, 2012 | EPI465705 |
| A/Vietnam/3028/2013 | Vietnam | March, 2013 | EPI497639 |
| A/Vietnam/0102/2015 | Vietnam | February, 2015 | EPI706481 |
| A/Vietnam/GS150197/2015 | Vietnam | April, 2015 | EPI768517 |
| A/Hanoi/Eli14419/2014 | Vietnam | June, 2014 | EPI956447 |
| A/Vietnam/0162/2015 | Vietnam | April, 2015 | EPI706497 |
| A/Niigata/10F557/2011 | Japan | March, 2011 | AEQ49890 |
| A/Niigata/12F053/2013 | Japan | January, 2013 | EPI1256204 |
| A/Niigata/13F003/2014 | Japan | January, 2014 | BAQ58425 |
| A/Niigata/13F270/2014.08 | Japan | February, 2014 | BAQ58415 |
| A/Niigata/13F311/2014.08 | Japan | February, 2014 | BAQ58405 |
| A/SAPPORO/35/2014 | Japan | January, 2014 | EPI507766 |
| A/SAPPORO/5/2014 | Japan | January, 2014 | EPI502981 |
| A/Nagasaki/13N018/2014 | Japan | January, 2014 | BAQ58605 |
| A/Nagasaki/13N057/2014 | Japan | January, 2014 | EPI567843 |
| A/Gunma/10G004/2011 | Japan | January, 2011 | JN790349 |
| A/Fukushima/74/2015 | Japan | September, 2015 | EPI748908 |
| A/Kyoto/13K003/2014 | Japan | January, 2014 | LC032849 |
| A/Okinawa/14T001/2014 | Japan | September, 2014 | EPI577742 |
| A/Gunma/13G001/2014 | Japan | January, 2014 | LC032905 |
| A/Hokkaido/12H20/2013 | Japan | January, 2013 | EPI1256203 |
| A/Niigata/14F019/2015 | Japan | January, 2015 | EPI580515 |
| A/Paraguay/185/2012 | South America | June, 2012 | EPI387974 |
| A/Paraguay/17/2013 | South America | July, 2013 | EPI484969 |
| A/Paraguay/64/2014 | South America | July, 2014 | EPI543034 |
| A/Peru/03/2015 | South America | April, 2015 | EPI629273 |
| A/Argentina/1171/2011 | South America | September, 2011 | EPI357968 |
| A/Argentina/42/2012 | South America | July, 2012 | EPI394727 |
| A/Argentina/22/2013 | South America | February, 2013 | EPI465469 |
| A/Argentina/115/2015 | South America | July, 2015 | EPI651831 |
| A/Bolivia/1516/2010 | South America | September, 2010 | EPI295423 |
| A/Bolivia/258/2011 | South America | August, 2011 | EPI366273 |
| A/Bolivia/3040/2012 | South America | August, 2012 | EPI404834 |
| A/Bolivia/559/2013 | South America | June, 2013 | EPI466897 |
| A/Bolivia/1115/2014 | South America | July, 2014 | EPI541549 |
| A/Parana/305/2014 | South America | June, 2014 | EPI588341 |
| A/Parana/822/2013 | South America | June, 2013 | EPI588367 |
| A/Parana/845/2012 | South America | July, 2012 | EPI588371 |
| A/Parana/951/2010 | South America | June, 2010 | EPI588382 |
| A/Rio Grande do Sul/361/2011 | South America | June, 2011 | EPI588430 |
| A/Parana/753/2015 | South America | September, 2015 | EPI1032027 |
| A/Santiago/14851/2010 | South America | June, 2010 | EPI280308 |
| A/Chile/293/2011 | South America | May, 2011 | EPI325567 |
| A/Iquique/44427/2012 | South America | June, 2012 | EPI394863 |
| A/Santiago/46965/2013 | South America | July, 2012 | EPI467229 |
| A/Santiago/66221/2014 | South America | October, 2014 | EPI562073 |
| A/Concepcion/39751/2015 | South America | June, 2015 | EPI668408 |
| A/Colombia/6868/2010 | South America | June, 2010 | EPI280292 |
| A/Colombia/1400/2011 | South America | December, 2011 | EPI362958 |
| A/Colombia/1359/2015 | South America | January, 2015 | EPI620315 |
| A/California/31/2010 | North America | December, 2010 | EPI302132 |
| A/Michigan/45/2015 | North America | September, 2015 | EPI859651 |
| A/Michigan/57/2015 | North America | October, 2015 | EPI706141 |
| A/California/33/2015 | North America | January, 2015 | EPI611009 |
| A/California/32/2011 | North America | November, 2011 | EPI348180 |
| A/California/12/2012 | North America | January, 2012 | EPI397889 |
| A/California/60/2013 | North America | December, 2013 | EPI509481 |
| A/California/37/2014 | North America | October, 2014 | EPI562082 |
| A/Alberta/RV1242/2011 | North America | April, 2011 | EPI366261 |
| A/Alberta/RV2960/2012 | North America | November, 2012 | EPI407374 |
| A/Canada-ON/RV2017/2010 | North America | January, 2010 | EPI304074 |
| A/Ontario/50/2013 | North America | December, 2013 | EPI509553 |
| A/Ontario/RV2184/2014 | North America | April, 2014 | EPI536862 |
| A/Alberta/RV2376/2015 | North America | September, 2015 | EPI680297 |
| A/Mexico/2880/2010 | North America | April, 2010 | EPI278878 |
| A/Mexico/239/2011 | North America | December, 2011 | EPI366293 |
| A/Mexico/689/2012 | North America | January, 2012 | EPI366299 |
| A/Mexico/3280/2013 | North America | October, 2013 | EPI503827 |
| A/Mexico/2493/2014 | North America | October, 2014 | EPI567664 |
| A/Mexico/2/2015 | North America | December, 2015 | EPI762610 |
| A/Texas/14/2010 | North America | November, 2010 | EPI301719 |
| A/Texas/07/2011 | North America | February, 2011 | EPI310130 |
| A/Texas/36/2012 | North America | March, 2012 | EPI377531 |
| A/Texas/10/2013 | North America | March, 2013 | EPI440927 |
| A/Texas/09/2014 | North America | January, 2014 | EPI521914 |
| A/Texas/78/2015 | North America | December, 2015 | EPI716826 |
| A/England/118/2010 | Europe | November, 2010 | EPI301401 |
| A/England/377/2015 | Europe | December, 2015 | EPI711925 |
| A/ENG/114940161/2011 | Europe | December, 2011 | EPI358504 |
| A/England/272/2010 | Europe | December, 2010 | EPI369827 |
| A/England/576/2012 | Europe | October, 2012 | EPI416397 |
| A/England/673/2013 | Europe | December, 2013 | EPI677733 |
| A/England/439/2014 | Europe | April, 2014 | EPI711775 |
| A/England/150/2015 | Europe | February, 2015 | EPI711812 |
| A/Paris/2516/2013 | Europe | December, 2013 | EPI501176 |
| A/Paris/2207/2010 | Europe | December, 2010 | EPI536113 |
| A/Paris/1230/2011 | Europe | January, 2010 | EPI536161 |
| A/Paris/1119/2012 | Europe | March, 2012 | EPI536169 |
| A/Paris/2302/2014 | Europe | December, 2014 | EPI556988 |
| A/Paris/1686/2015 | Europe | February, 2015 | EPI672480 |
| A/Berlin/23/2011 | Europe | January, 2011 | EPI576265 |
| A/Berlin/14/2010 | Europe | December, 2010 | EPI576266 |
| A/Hessen/4/2013 | Europe | January, 2013 | EPI576336 |
| A/Berlin/167/2012 | Europe | December, 2012 | EPI576338 |
| A/Berlin/30/2014 | Europe | February, 2014 | EPI576364 |
| A/Berlin/166/2015 | Europe | October, 2015 | EPI678532 |
| A/Berlin/177/2015 | Europe | December, 2012 | EPI700122 |
| A/St.Petersburg/14/2010 | Europe | January, 2010 | EPI310245 |
| A/Moscow/IIV-45/2012 | Europe | February, 2012 | EPI390723 |
| A/Vladivostok/93/2013 | Europe | December, 2013 | EPI505319 |
| A/St.Petersburg/100/2011 | Europe | March, 2011 | EPI316435 |
| A/St. Petersburg/27/2011 | Europe | February, 2011 | EPI319527 |
| A/ST PETERSBURG/61/2015 | Europe | February, 2015 | EPI672602 |
| A/Switzerland/3628801/2010 | Europe | August, 2010 | EPI295629 |
| A/Switzerland/485/2015 | Europe | January, 2015 | EPI557996 |
| A/St Petersburg/40/2014 | Europe | April, 2014 | EPI536853 |
| A/Slovenia/2903/2015 | Europe | October, 2015 | EPI687827 |
| A/Norway/1675/2013 | Europe | March, 2013 | EPI466588 |
| A/St Petersburg/1/2015 | Europe | December, 2015 | EPI742173 |
| A/Ghana/FS/10-4466/2010 | Africa | September, 2010 | EPI301415 |
| A/Ghana/ARI1181/2011 | Africa | October, 2011 | EPI352295 |
| A/Ghana/DILI-0007/2013 | Africa | January, 2013 | EPI466551 |
| A/Ghana/FS-13-1090/2012 | Africa | November, 2012 | EPI498419 |
| A/Ghana/DILI-0620/2014 | Africa | October, 2014 | EPI541037 |
| A/Ghana/FIP_206/2015 | Africa | April, 2015 | EPI624688 |
| A/Algeria/G388/2010 | Africa | December, 2010 | EPI319430 |
| A/Annaba/G687/2012 | Africa | October, 2012 | EPI460646 |
| A/Congo/2275/2014 | Africa | November, 2014 | EPI575857 |
| A/Congo/2278/2014 | Africa | November, 2014 | EPI575892 |
| A/Congo/1939/2015 | Africa | October, 2015 | EPI733219 |
| A/Congo/1942/2015 | Africa | October, 2015 | EPI742364 |
| A/Egypt/96/2010 | Africa | December, 2010 | EPI301906 |
| A/Egypt/68/2013 | Africa | November, 2013 | EPI301906 |
| A/Egypt/4372/2014 | Africa | October, 2014 | EPI587766 |
| A/Egypt/6/2015 | Africa | September, 2015 | EPI748892 |
| A/Ethiopia/217/2010 | Africa | December, 2010 | EPI309880 |
| A/Ethiopia/186/2012 | Africa | April, 2012 | EPI386036 |
| A/Ethiopia/1149/2014 | Africa | October, 2014 | EPI564944 |
| A/Ethiopia/1784/2015 | Africa | December, 2015 | EPI732749 |
| A/Johannesburg/150/2011 | Africa | July, 2011 | EPI346655 |
| A/South Africa/17170/2010 | Africa | September, 2010 | EPI361673 |
| A/South Africa/2226/2013 | Africa | April, 2013 | EPI466618 |
| A/South Africa/3626/2013 | Africa | June, 2013 | EPI577031 |
| A/South Africa/606/2014 | Africa | February, 2014 | EPI539536 |
| A/South Africa/5154/2015 | Africa | August, 2015 | EPI684418 |
| A/Uganda/617/2011 | Africa | November, 2011 | EPI356256 |
| A/SYDNEY/100/2011 | Australia | July, 2011 | EPI379470 |
| A/Sydney/1003/2015 | Australia | June, 2015 | EPI636201 |
| A/SYDNEY/1098/2014 | Australia | September, 2014 | EPI551431 |
| A/SYDNEY/1103/2014 | Australia | September, 2014 | EPI551434 |
| A/SYDNEY/12/2011 | Australia | May, 2011 | EPI333026 |
| A/Sydney/185/2015 | Australia | August, 2015 | EPI704166 |
| A/Sydney/215/2015 | Australia | November, 2015 | EPI704087 |
| A/SYDNEY/31/2010 | Australia | October, 2010 | EPI302364 |
| A/Sydney/517/2010 | Australia | October, 2010 | EPI294397 |
| A/SYDNEY/70/2013 | Australia | February, 2013 | EPI526774 |
| A/SYDNEY/74/2011 | Australia | July, 2011 | EPI349307 |
| A/SYDNEY/82/2013 | Australia | November, 2013 | EPI529400 |
| A/Sydney/9/2015 | Australia | February, 2015 | EPI629886 |
| A/Sydney/DD3-48/2010 | Australia | September, 2010 | EPI321211 |
| A/AUCKLAND/118/2010 | New Zealand | July, 2010 | EPI294414 |
| A/AUCKLAND/528/2013 | New Zealand | September, 2013 | EPI526745 |
| A/AUCKLAND/64/2012 | New Zealand | August, 2012 | EPI417243 |
| A/AUCKLAND/8/2014 | New Zealand | September, 2014 | EPI551353 |
| A/CHRISTCHURCH/105/2011 | New Zealand | October, 2011 | EPI370266 |
| A/CHRISTCHURCH/11/2012 | New Zealand | June, 2012 | EPI394304 |
| A/Christchurch/16/2010 | New Zealand | July, 2010 | EPI280344 |
| A/CHRISTCHURCH/3/2012 | New Zealand | May, 2012 | EPI394215 |
| A/CHRISTCHURCH/523/2013 | New Zealand | August, 2013 | EPI491318 |
| A/CHRISTCHURCH/535/2013 | New Zealand | August, 2013 | EPI526707 |
| A/Christchurch/538/2014 | New Zealand | August, 2014 | EPI748142 |
| A/CHRISTCHURCH/6/2010 | New Zealand | July, 2010 | EPI279157 |
| A/CHRISTCHURCH/60/2011 | New Zealand | September, 2011 | EPI357616 |
| A/BRISBANE/153/2012 | New Zealand | July, 2012 | EPI417249 |
| A/BRISBANE/96/2012 | New Zealand | June, 2012 | EPI450231 |
| A/New Zealand/0498/2014 | New Zealand | May, 2014 | EPI537939 |
| A(H3N2) |  |  |  |
| A/Nepal/1336/2014 | Nepal | August, 2014 | EPI715765 |
| A/Nepal/1375A/2013 | Nepal | August, 2013 | EPI578938 |
| A/Nepal/1474A/2013 | Nepal | August, 2013 | EPI578946 |
| A/Nepal/401/2015 | Nepal | March, 2015 | EPI715771 |
| A/Nepal/420/2014 | Nepal | March, 2015 | EPI715755 |
| A/Afghanistan/0855/2015 | Afghanistan | December, 2015 | EPI746868 |
| A/Afghanistan/463/2015 | Afghanistan | December, 2015 | EPI824993 |
| A/Kabul/1514A01305429N/2013 | Afghanistan | January, 2013 | EPI843349 |
| A/Kabul/1514A01305430N/2013 | Afghanistan | January, 2013 | EPI843350 |
| A/Bangladesh/03/2012 | Bangladesh | August, 2012 | EPI985059 |
| A/Bangladesh/04/2012 | Bangladesh | August, 2012 | EPI985060 |
| A/Bangladesh/10009/2014 | Bangladesh | June, 2014 | EPI548900 |
| A/Bangladesh/1151/2015 | Bangladesh | October, 2015 | EPI775464 |
| A/Bangladesh/1539/2011 | Bangladesh | July, 2011 | EPI335866 |
| A/Bangladesh/2012/2010 | Bangladesh | August, 2010 | EPI609292 |
| A/Bangladesh/2021/2013 | Bangladesh | October, 2013 | EPI508656 |
| A/Bangladesh/203006/2015 | Bangladesh | December, 2015 | EPI701817 |
| A/Bangladesh/2596/2012 | Bangladesh | August, 2012 | EPI397570 |
| A/Bangladesh/3004/2014 | Bangladesh | May, 2014 | EPI540009 |
| A/Bangladesh/3010/2015 | Bangladesh | September, 2015 | EPI768560 |
| A/Bangladesh/3161/2010 | Bangladesh | September, 2010 | EPI609409 |
| A/Bangladesh/3564/2011 | Bangladesh | July, 2011 | EPI340915 |
| A/Bangladesh/3569/2014 | Bangladesh | May, 2014 | EPI540069 |
| A/Bangladesh/4007/2014 | Bangladesh | June, 2014 | EPI541577 |
| A/Bangladesh/5004/2010 | Bangladesh | July, 2010 | EPI608420 |
| A/Bangladesh/5006/2011 | Bangladesh | August, 2011 | EPI347469 |
| A/Bangladesh/5071/2011 | Bangladesh | May, 2011 | EPI342351 |
| A/Bangladesh/7006/2015 | Bangladesh | August, 2015 | EPI670894 |
| A/Bangladesh/8004/2010 | Bangladesh | September, 2010 | EPI608457 |
| A/Bangladesh/8004/2013 | Bangladesh | July, 2013 | EPI486582 |
| A/Bangladesh/8011/2013 | Bangladesh | November, 2013 | EPI508663 |
| A/Bangladesh/8598/2013 | Bangladesh | November, 2013 | EPI513295 |
| A/Bangladesh/8698/2014 | Bangladesh | May, 2014 | EPI513295 |
| A/Bangladesh/910004/2015 | Bangladesh | September, 2015 | EPI752089 |
| A/Bangladesh/9269/2013 | Bangladesh | May, 2013 | EPI492717 |
| A/Bangladesh/9334/2013 | Bangladesh | May, 2013 | EPI484573 |
| A/Cambodia/0840/2015 | Cambodia | August, 2015 | EPI711282 |
| A/Cambodia/0924/2015 | Cambodia | August, 2015 | EPI702040 |
| A/Cambodia/0929/2015 | Cambodia | September, 2015 | EPI711019 |
| A/Cambodia/1/2010 | Cambodia | June, 2010 | EPI608455 |
| A/CAMBODIA/10/2012 | Cambodia | July, 2012 | EPI394253 |
| A/Cambodia/1244/2014 | Cambodia | November, 2014 | EPI579366 |
| A/CAMBODIA/31/2012 | Cambodia | June, 2012 | EPI394322 |
| A/CAMBODIA/5/2012 | Cambodia | July, 2012 | EPI394250 |
| A/CAMBODIA/55/2011 | Cambodia | December, 2011 | EPI370321 |
| A/Cambodia/590/2014 | Cambodia | May, 2014 | EPI540001 |
| A/Cambodia/AD04410/2014 | Cambodia | October, 2014 | EPI629750 |
| A/Cambodia/U0825342/2010 | Cambodia | August, 2010 | EPI464420 |
| A/Cambodia/U307/2010 | Cambodia | August, 2010 | EPI341406 |
| A/Cambodia/V0902310/2011 | Cambodia | August, 2011 | EPI447005 |
| A/Cambodia/V1116321/2011 | Cambodia | November, 2011 | EPI447017 |
| A/CAMBODIA/X0717310/2013 | Cambodia | July, 2013 | EPI491283 |
| A/Beijing/53593/2014 | China | March, 2014 | EPI598854 |
| A/Beijing/59100/2014 | China | October, 2014 | EPI598847 |
| A/Guangdong/1104/2012 | China | March, 2012 | EPI539343 |
| A/Guangdong/187/2011 | China | September, 2011 | EPI539349 |
| A/Guangdong/192/2011 | China | September, 2011 | EPI539351 |
| A/Guangdong/222/2011 | China | November, 2011 | EPI539353 |
| A/Guangdong/322/2010 | China | August, 2010 | EPI326365 |
| A/Guangdong/947/2012 | China | March, 2012 | EPI539355 |
| A/Hangzhou/766/2010 | China | September, 2010 | EPI622594 |
| A/Hangzhou/790/2010 | China | September, 2010 | EPI622596 |
| A/Hangzhou/A126/2013 | China | February, 2013 | EPI622618 |
| A/Hangzhou/A379/2012 | China | August, 2012 | EPI622613 |
| A/Hangzhou/B334/2012 | China | July, 2012 | EPI622610 |
| A/Hangzhou/B48/2013 | China | January, 2013 | EPI622616 |
| A/Hong Kong/4801/2014 | China | February, 2014 | EPI653201 |
| A/Hong Kong/5738/2014 | China | April, 2014 | EPI541442 |
| A/Nanjing/1654/2010 | China | September, 2010 | EPI297531 |
| A/Suzhou/1275/2013 | China | April, 2013 | EPI498075 |
| A/Chhattisgarh/9873/2012 | India | October, 2012 | EPI527662 |
| A/Delhi/1191/2013 | India | February, 2013 | EPI527672 |
| A/Delhi/1549/2013 | India | February, 2013 | EPI527678 |
| A/Delhi/1720/2013 | India | February, 2013 | EPI527682 |
| A/Delhi/2274/2013 | India | February, 2013 | EPI527686 |
| A/Delhi/567/2013 | India | February, 2013 | EPI527667 |
| A/Delhi/764/2013 | India | February, 2013 | EPI527670 |
| A/Haryana/1256/2013 | India | February, 2013 | EPI527674 |
| A/Haryana/2396/2013 | India | February, 2013 | EPI527688 |
| A/Haryana/707/2013 | India | February, 2013 | EPI527669 |
| A/Mumbai/5256/2010 | India | August, 2010 | EPI538941 |
| A/Mumbai/5257/2010 | India | August, 2010 | EPI538940 |
| A/Mumbai/5420/2010 | India | August, 2010 | EPI538942 |
| A/Mumbai/5460/2010 | India | September, 2010 | EPI538939 |
| A/Uttarakhand/8853/2011 | India | June, 2011 | EPI527659 |
| A/Indonesia/Nihrd-Mmj337/2015 | Indonesia | July, 2015 | EPI668097 |
| A/Indonesia/Nihrds-0504/2014 | Indonesia | January, 2014 | EPI543663 |
| A/Jakarta/FLUEJKSV0031/2012 | Indonesia | February, 2012 | EPI842642 |
| A/Jakarta/FLUEJKSV0033/2012 | Indonesia | February, 2012 | EPI842643 |
| A/Israel/10/2012 | Israel | October, 2012 | EPI445886 |
| A/Israel/20/2013 | Israel | January, 2013 | EPI445888 |
| A/Israel/45/2011 | Israel | December, 2011 | EPI354185 |
| A/Israel/5/11/2011 | Israel | January, 2011 | EPI319258 |
| A/Israel/P-349/2014 | Israel | December, 2014 | EPI563113 |
| A/Israel/P-451/2015 | Israel | January, 2015 | EPI620485 |
| A/Israel/P-513/2015 | Israel | January, 2015 | EPI620487 |
| A/Israel/Z125/2013 | Israel | October, 2013 | EPI515191 |
| A/Israel/Z-1263/2014 | Israel | February, 2014 | EPI539818 |
| A/Lebanon/11L002/2011 | Lebanon | November, 2011 | EPI530826 |
| A/Lebanon/11L007/2011 | Lebanon | August, 2011 | EPI530830 |
| A/Lebanon/11L013/2011 | Lebanon | July, 2011 | EPI530827 |
| A/Lebanon/11L023/2012 | Lebanon | January, 2012 | EPI530818 |
| A/Lebanon/11L040/2012 | Lebanon | February, 2012 | EPI530820 |
| A/Lebanon/11L046/2012 | Lebanon | February, 2012 | EPI530829 |
| A/Lebanon/14L45/2014 | Lebanon | January, 2014 | EPI580489 |
| A/Lebanon/14L56/2014 | Lebanon | January, 2014 | EPI580497 |
| A/Lebanon/14L78/2014 | Lebanon | February, 2014 | EPI580505 |
| A/Laos/1110/2015 | Laos | October, 2015 | EPI730975 |
| A/Laos/1115/2014 | Laos | October, 2014 | EPI584626 |
| A/Laos/348/2013 | Laos | October, 2013 | EPI510890 |
| A/Laos/732/2014 | Laos | November, 2014 | EPI622272 |
| A/Laos/82/2010 | Laos | January, 2010 | EPI606387 |
| A/Laos/854/2010 | Laos | September, 2010 | EPI607443 |
| A/Laos/890/2012 | Laos | November, 2012 | EPI436276 |
| A/Laos/896/2010 | Laos | September, 2010 | EPI608566 |
| A/Laos/977/2015 | Laos | September, 2015 | EPI731013 |
| A/Laos/I1002/2015 | Laos | September, 2015 | EPI706577 |
| A/Laos/I1031/2010 | Laos | October, 2010 | EPI606620 |
| A/Laos/I1066/2015 | Laos | October, 2015 | EPI706609 |
| A/Laos/I1126/2013 | Laos | November, 2013 | EPI541923 |
| A/Laos/I1223/2014 | Laos | December, 2014 | EPI610563 |
| A/Laos/I1235/2014 | Laos | December, 2014 | EPI610565 |
| A/Laos/I461/2013 | Laos | June, 2013 | EPI497999 |
| A/Laos/I462/2012 | Laos | June, 2012 | EPI413258 |
| A/Laos/I602/2012 | Laos | August, 2012 | EPI413261 |
| A/Laos/I669/2012 | Laos | September, 2012 | EPI413264 |
| A/Laos/I700/2010 | Laos | August, 2010 | EPI294024 |
| A/Laos/I720/2011 | Laos | October, 2011 | EPI372008 |
| A/Laos/I799/2011 | Laos | October, 2011 | EPI375610 |
| A/Laos/I810/2015 | Laos | August, 2015 | EPI719012 |
| A/Laos/I832/2011 | Laos | November, 2011 | EPI375626 |
| A/Laos/I894/2011 | Laos | November, 2011 | EPI372028 |
| A/Laos/I962/2013 | Laos | October, 2013 | EPI543747 |
| A/Laos/O502/2014 | Laos | October, 2014 | EPI578982 |
| A/Laos/SA119/2011 | Laos | October, 2011 | EPI375614 |
| A/Laos/SA329/2012 | Laos | November, 2012 | EPI445763 |
| A/Laos/SA398/2013 | Laos | October, 2013 | EPI541929 |
| A/MALAYSIA/10/2013 | Malaysia | November, 2013 | EPI529546 |
| A/MALAYSIA/20/2014 | Malaysia | February, 2014 | EPI541122 |
| A/Malaysia/22/2013 | Malaysia | October, 2013 | EPI567269 |
| A/Malaysia/2867/2015 | Malaysia | December, 2015 | EPI765051 |
| A/MALAYSIA/341/2012 | Malaysia | February, 2012 | EPI394084 |
| A/MALAYSIA/6/2014 | Malaysia | April, 2014 | EPI541131 |
| A/MALAYSIA/69102/2012 | Malaysia | December, 2012 | EPI450246 |
| A/Malaysia/69722/2015 | Malaysia | November, 2015 | EPI868909 |
| A/BiliranTB5/0473/2015 | Philippine | July, 2015 | EPI838378 |
| A/BiliranTB5/0529/2015 | Philippine | July, 2015 | EPI838382 |
| A/BiliranTB8/0138/2014 | Philippine | April, 2014 | EPI838363 |
| A/BiliranTB9/0323/2014 | Philippine | May, 2014 | EPI838367 |
| A/Philippines/04/2010 | Philippine | August, 2010 | EPI606721 |
| A/Philippines/06/2010 | Philippine | September, 2010 | EPI607190 |
| A/PHILIPPINES/1197/2011 | Philippine | March, 2011 | EPI346260 |
| A/PHILIPPINES/3/2012 | Philippine | January, 2012 | EPI394256 |
| A/PHILIPPINES/3948/2011 | Philippine | October, 2011 | EPI370329 |
| A/Incheon/2384/2010 | South Korea | December, 2010 | EPI608387 |
| A/Korea/47/2012 | South Korea | January, 2012 | EPI841897 |
| A/Korea/KUMC-GR590/2011 | South Korea | December, 2011 | EPI1061768 |
| A/Seoul/A1098/2014 | South Korea | December, 2014 | EPI1159700 |
| A/Seoul/A569/2013 | South Korea | March, 2013 | EPI1159722 |
| A/Seoul/APD326/2015 | South Korea | March, 2013 | EPI1159730 |
| A/Singapore/22/2012 | Singapore | June, 2012 | EPI426131 |
| A/Singapore/516/2010 | Singapore | April, 2012 | EPI608433 |
| A/Singapore/GP409/2015 | Singapore | March, 2012 | EPI730533 |
| A/Singapore/H2011.518/2011 | Singapore | July, 2011 | EPI458062 |
| A/Singapore/H2013.425/2013 | Singapore | June, 2013 | AIG44809 |
| A/Singapore/H2013.721f/2013 | Singapore | September, 2013 | EPI704642 |
| A/Singapore/KK943/2014 | Singapore | October, 2014 | EPI746458 |
| A/Singapore/S2010.359a/2010 | Singapore | May, 2010 | AIG44729 |
| A/Bangkok/SI-MI01/2015 | Thailand | February, 2015 | AJY79371 |
| A/Bangkok/SI-MI04/2015 | Thailand | February, 2015 | AJY79374 |
| A/Bangkok/SI-MI06/2014 | Thailand | February, 2015 | AJY79376 |
| A/Bangkok/SI-MI07/2014 | Thailand | March, 2012 | AJY79377 |
| A/Bangkok/SI-MI09/2014 | Thailand | July, 2014 | AJY79379 |
| A/Bangkok/SI-MI11/2014 | Thailand | October, 2014 | AJY79381 |
| A/Thailand/CU-A134/2013 | Thailand | June, 2013 | AJE62892 |
| A/Thailand/CU-A164/2013 | Thailand | July, 2013 | AJE62895 |
| A/Thailand/CU-B4836/2011 | Thailand | July, 2013 | AJE62841 |
| A/Thailand/CU-B5105/2011 | Thailand | August, 2011 | AJE62844 |
| A/Thailand/CU-B5928/2011 | Thailand | November, 2011 | AJE62859 |
| A/Thailand/CU-B7189/2012 | Thailand | December, 2015 | AJE62872 |
| A/Thailand/CU-B7596/2013 | Thailand | February, 2013 | AJE62881 |
| A/Thailand/CU-H3656/2014 | Thailand | September, 2014 | AJE62940 |
| A/Thailand/PY08156/2010 | Thailand | August, 2010 | AJJ99005 |
| A/Thailand/SN10452/2010 | Thailand | October, 2010 | AJJ98945 |
| A/Vietnam/13V H3-10/2012 | Vietnam | July, 2012 | EPI567943 |
| A/Vietnam/13V H3-3/2012 | Vietnam | July, 2012 | EPI567889 |
| A/Vietnam/154/2015 | Vietnam | August, 2015 | EPI730265 |
| A/Vietnam/158/2015 | Vietnam | October, 2015 | EPI722776 |
| A/Vietnam/167/2010 | Vietnam | February, 2010 | EPI609674 |
| A/Vietnam/2/2014 | Vietnam | May, 2014 | EPI629996 |
| A/Vietnam/4/2014 | Vietnam | May, 2014 | EPI630002 |
| A/Vietnam/473/2013 | Vietnam | October, 2013 | EPI499296 |
| A/Vietnam/481/2013 | Vietnam | October, 2013 | EPI499268 |
| A/Vietnam/838/2010 | Vietnam | July, 2010 | EPI608383 |
| A/Gunma/14G002/2015 | Japan | January, 2015 | EPI577660 |
| A/Gunma/14G003/2015 | Japan | January, 2015 | EPI577660 |
| A/Hokkaido/13H009/2014 | Japan | January, 2014 | EPI581402 |
| A/Hokkaido/13H011/2014 | Japan | January, 2014 | EPI581317 |
| A/Kyoto/13K012/2014 | Japan | January, 2014 | EPI581496 |
| A/Kyoto/13K025/2014 | Japan | February, 2014 | EPI581488 |
| A/NAGASAKI/12/2015 | Japan | March, 2015 | EPI630481 |
| A/Nagasaki/14N013/2014 | Japan | December, 2014 | EPI580625 |
| A/Niigata/10F004/2011 | Japan | January, 2011 | EPI340487 |
| A/Niigata/10F013/2011 | Japan | January, 2011 | EPI340486 |
| A/Niigata/11F020/2012 | Japan | January, 2012 | EPI1256200 |
| A/Niigata/11F028/2012 | Japan | January, 2012 | EPI1256201 |
| A/Niigata/13F024/2014 | Japan | August, 2014 | EPI581239 |
| A/Niigata/13NU3/2014 | Japan | January, 2014 | LC033057 |
| A/NIIGATA-C/44/2015 | Japan | May, 2015 | EPI630405 |
| A/Okinawa/14T006/2015 | Japan | January, 2015 | EPI580601 |
| A/Okinawa/14T007/2015 | Japan | January, 2015 | EPI580609 |
| A/Niigata/13F173/2014 | Japan | January, 2014 | EPI581225 |
| A/Argentina/01/2010 | South America | December, 2010 | EPI608353 |
| A/Argentina/11093/2014 | South America | July, 2014 | EPI547719 |
| A/Argentina/179/2011 | South America | June, 2011 | EPI334622 |
| A/Argentina/234/2015 | South America | July, 2015 | EPI643864 |
| A/Argentina/433/2013 | South America | June, 2013 | EPI465513 |
| A/Bolivia/1053/2010 | South America | August, 2010 | EPI609136 |
| A/Bolivia/340/2011 | South America | June, 2011 | EPI342360 |
| A/Bolivia/502/2015 | South America | August, 2015 | EPI684259 |
| A/Bolivia/902/2013 | South America | May, 2013 | EPI468134 |
| A/Bolivia/906/2014 | South America | July, 2014 | EPI547758 |
| A/Chile/64/2011 | South America | May, 2011 | EPI342327 |
| A/Chile/8196/2010 | South America | August, 2010 | EPI609648 |
| A/Copiapo/47381/2014 | South America | July, 2014 | EPI555135 |
| A/Mendoza/176711/2012 | South America | September, 2012 | EPI407064 |
| A/Parana/1604/2010 | South America | October, 2010 | EPI646452 |
| A/Parana/404/2012 | South America | June, 2012 | EPI646558 |
| A/Peru/PER262/2012 | South America | April, 2012 | EPI496663 |
| A/Peru/PER271/2011 | South America | September, 2011 | EPI496724 |
| A/Porto Alegre/LACENRS-1406/2014 | South America | July, 2014 | EPI996479 |
| A/Porto Alegre/LACENRS-275/2013 | South America | March, 2013 | EPI997368 |
| A/Porto Alegre/LACENRS-820/2015 | South America | May, 2015 | EPI997518 |
| A/Porto Alegre/LACENRS-822/2011 | South America | July, 2011 | EPI997595 |
| A/Santiago/11616/2015 | South America | February, 2015 | EPI678748 |
| A/Santiago/35234/2012 | South America | May, 2012 | EPI387805 |
| A/Santiago/35652/2013 | South America | May, 2013 | EPI467253 |
| A/Uruguay/06/2012 | South America | July, 2012 | EPI397571 |
| A/Uruguay/2214/2010 | South America | November, 2010 | EPI609478 |
| A/Uruguay/322/2013 | South America | June, 2013 | EPI467428 |
| A/Uruguay/359/2014 | South America | July, 2014 | EPI543702 |
| A/Uruguay/44/2015 | South America | July, 2014 | EPI662385 |
| A/California/02/2014 | North America | January, 2014 | EPI517095 |
| A/California/05/2015 | North America | January, 2015 | EPI571846 |
| A/California/10/2010 | North America | May, 2010 | EPI427045 |
| A/California/15/2013 | North America | February, 2013 | EPI439454 |
| A/California/16/2010 | North America | July, 2010 | EPI342169 |
| A/California/30/2011 | North America | November, 2011 | EPI347475 |
| A/California/35/2012 | North America | July, 2012 | EPI387778 |
| A/Mexico/1546/2015 | North America | May, 2015 | EPI649890 |
| A/Mexico/3040/2014 | North America | December, 2014 | EPI565849 |
| A/Mexico/4268/2010 | North America | August, 2010 | EPI301119 |
| A/Mexico/InDRE2554/2011 | North America | April, 2011 | EPI395897 |
| A/Texas/115/2012 | North America | December, 2012 | EPI954422 |
| A/Texas/16/2015 | North America | February, 2015 | EPI934667 |
| A/Texas/18/2011 | North America | February, 2011 | EPI438339 |
| A/Texas/21/2013 | North America | January, 2013 | EPI954406 |
| A/Texas/23/2010 | North America | December, 2010 | EPI309373 |
| A/Texas/50/2012 | North America | April, 2012 | EPI556816 |
| A/Texas/99/2014 | North America | November, 2014 | EPI565452 |
| A/Yucatan/238/2012 | North America | December, 2012 | EPI942440 |
| A/Yucatan/362/2013 | North America | September, 2013 | EPI943328 |
| A/Berlin/168/2012 | Europe | December, 2012 | EPI576354 |
| A/Berlin/215/2013 | Europe | December, 2013 | EPI499594 |
| A/Berlin/73/2014 | Europe | May, 2014 | EPI576376 |
| A/Berlin/T621/2011 | Europe | December, 2011 | EPI576316 |
| A/Brandenburg/01/2010 | Europe | October, 2010 | EPI309443 |
| A/ENG/50200176/2014 | Europe | December, 2014 | EPI626794 |
| A/ENG/50260152/2015 | Europe | January, 2015 | EPI626837 |
| A/England/215/2011 | Europe | February, 2011 | EPI393290 |
| A/England/270/2010 | Europe | December, 2010 | EPI609564 |
| A/England/651/2013 | Europe | December, 2013 | EPI503286 |
| A/England/676/2012 | Europe | December, 2012 | EPI416496 |
| A/Giessen/2390/2015 | Europe | February, 2015 | EPI625893 |
| A/Lyon/1135/2010 | Europe | December, 2010 | EPI319262 |
| A/Moscow/253/2014 | Europe | December, 2014 | EPI574636 |
| A/Moscow/RII13/2012 | Europe | March, 2012 | EPI401241 |
| A/Netherlands/525/2014 | Europe | December, 2014 | EPI574644 |
| A/Norway/2605/2015 | Europe | October, 2015 | EPI678132 |
| A/Norway/780/2010 | Europe | July, 2010 | EPI278633 |
| A/Paris/027/2011 | Europe | January, 2011 | EPI357461 |
| A/Paris/1787/2015 | Europe | October, 2015 | EPI678105 |
| A/Paris/2306/2014 | Europe | December, 2014 | EPI574650 |
| A/Paris/2479/2013 | Europe | December, 2013 | EPI501192 |
| A/Paris/647/2012 | Europe | January, 2012 | EPI535733 |
| A/Slovenia/1411/2015 | Europe | March, 2015 | EPI630751 |
| A/Slovenia/2528/2013 | Europe | December, 2013 | EPI503345 |
| A/Slovenia/537/2011 | Europe | January, 2011 | EPI335697 |
| A/Slovenia/622/2014 | Europe | February, 2014 | EPI536400 |
| A/Slovenia/637/2012 | Europe | March, 2012 | EPI377402 |
| A/St. Petersburg/21/2011 | Europe | February, 2011 | EPI326327 |
| A/St. Petersburg/428/2013 | Europe | September, 2013 | EPI498397 |
| A/St Petersburg/31/2015 | Europe | January, 2015 | EPI633783 |
| A/Switzerland/9715293/2013 | Europe | December, 2013 | EPI674736 |
| A/Vladivostok/11/2010 | Europe | November, 2010 | EPI367621 |
| A/Cairo/140/2012 | Africa | December, 2012 | EPI417005 |
| A/Cairo/153/2013 | Africa | January, 2013 | EPI445840 |
| A/Cairo/66/2011 | Africa | December, 2011 | EPI368880 |
| A/Casablanca/228/2010 | Africa | December, 2010 | EPI340903 |
| A/Central African Republic/878/2015 | Africa | August, 2015 | EPI746021 |
| A/Egypt/4870/2014 | Africa | November, 2014 | EPI612365 |
| A/Ethiopia/141/2012 | Africa | May, 2012 | EPI387757 |
| A/Ethiopia/159/2014 | Africa | September, 2014 | EPI565755 |
| A/Ethiopia/1780/2015 | Africa | December, 2015 | EPI730927 |
| A/Ethiopia/916/2013 | Africa | December, 2013 | EPI508678 |
| A/Ghana/DILI-15-0233/2015 | Africa | March, 2015 | EPI624555 |
| A/Ghana/FS/10-4462/2010 | Africa | September, 2010 | EPI301364 |
| A/Ghana/FS-0514/2014 | Africa | May, 2014 | EPI541440 |
| A/Ghana/FS-11-1981/2011 | Africa | November, 2011 | EPI352738 |
| A/Ghana/FS-12-758/2012 | Africa | June, 2012 | EPI392260 |
| A/Ghana/FS-13-951/2013 | Africa | September, 2013 | EPI515181 |
| A/Johannesburg/113/2011 | Africa | May, 2011 | EPI335730 |
| A/Johannesburg/3303/2012 | Africa | June, 2012 | EPI405936 |
| A/Johannesburg/34/2010 | Africa | July, 2010 | EPI287090 |
| A/Johannesburg/87/2010 | Africa | July, 2010 | EPI287096 |
| A/MOROCCO/47/2011 | Africa | October, 2011 | EPI393380 |
| A/Nigeria/235/2015 | Africa | September, 2015 | EPI693663 |
| A/Nigeria/3007/2010 | Africa | August, 2010 | EPI295242 |
| A/Nigeria/4126/2011 | Africa | March, 2011 | EPI331245 |
| A/Nigeria/5402/2012 | Africa | February, 2012 | EPI436285 |
| A/Nigeria/71/2014 | Africa | June, 2014 | EPI626549 |
| A/Nigeria/9999/2013 | Africa | October, 2013 | EPI531719 |
| A/South Africa/3941/2013 | Africa | June, 2013 | EPI466973 |
| A/South Africa/4090/2014 | Africa | May, 2014 | EPI539842 |
| A/South Africa/R0740/2015 | Africa | March, 2015 | EPI630771 |
| A/Sydney/1005/2014 | Australia | July, 2014 | EPI629869 |
| A/Sydney/1013/2015 | Australia | August, 2015 | EPI676188 |
| A/Sydney/1065/2014 | Australia | September, 2014 | EPI629871 |
| A/SYDNEY/13/2010 | Australia | August, 2010 | EPI294182 |
| A/Sydney/195/2012 | Australia | August, 2010 | EPI417294 |
| A/Sydney/224/2015 | Australia | September, 2015 | EPI750878 |
| A/SYDNEY/245/2012 | Australia | September, 2012 | EPI417297 |
| A/Sydney/248/2015 | Australia | September, 2015 | EPI750868 |
| A/SYDNEY/27/2011 | Australia | June, 2011 | EPI346371 |
| A/Sydney/530/2014 | Australia | December, 2014 | EPI636236 |
| A/SYDNEY/69/2013 | Australia | November, 2013 | EPI526807 |
| A/SYDNEY/83/2013 | Australia | November, 2013 | EPI529557 |
| A/SYDNEY/97/2011 | Australia | November, 2011 | EPI370114 |
| A/Sydney/DD2-02/2010 | Australia | September, 2010 | EPI318440 |
| A/Victoria/361/2011 | Australia | October, 2011 | EPI349103 |
| A/Auckland/10/2015 | New Zealand | July, 2015 | EPI676140 |
| A/AUCKLAND/2/2011 | New Zealand | July, 2011 | EPI346266 |
| A/AUCKLAND/544/2013 | New Zealand | September, 2013 | EPI526760 |
| A/AUCKLAND/6/2014 | New Zealand | September, 2014 | EPI551835 |
| A/AUCKLAND/61/2012 | New Zealand | July, 2012 | EPI417240 |
| A/Christchurch/1/2014 | New Zealand | February, 2014 | EPI540693 |
| A/CHRISTCHURCH/25/2010 | New Zealand | February, 2010 | EPI294309 |
| A/CHRISTCHURCH/25/2012 | New Zealand | July, 2012 | EPI394340 |
| A/Christchurch/28/2011 | New Zealand | August, 2011 | EPI357574 |
| A/CHRISTCHURCH/49/2010 | New Zealand | September, 2010 | EPI294312 |
| A/CHRISTCHURCH/50/2010 | New Zealand | September, 2010 | EPI294315 |
| A/Christchurch/503/2015 | New Zealand | June, 2015 | EPI652760 |
| A/CHRISTCHURCH/507/2013 | New Zealand | May, 2013 | EPI466070 |
| A/Christchurch/512/2012 | New Zealand | July, 2012 | EPI438689 |
| A/CHRISTCHURCH/526/2013 | New Zealand | August, 2013 | EPI491330 |
| A/CHRISTCHURCH/54/2011 | New Zealand | August, 2011 | EPI346350 |
| B(Victoria)-HA |  |  |  |
| B/Nepal/128/2010 | Nepal | August, 2010 | EPI294819 |
| B/Nepal/188/2010 | Nepal | August, 2010 | EPI294822 |
| B/Bangladesh/8275/2010 | Bangladesh | July, 2010 | EPI291609 |
| B/Bangladesh/3527/2010 | Bangladesh | September, 2010 | EPI294702 |
| B/Bangladesh/4622/2011 | Bangladesh | July, 2011 | EPI340813 |
| B/Bangladesh/8008/2011 | Bangladesh | August, 2011 | EPI366201 |
| B/Bangladesh/3249/2012 | Bangladesh | October, 2012 | EPI408620 |
| B/Bangladesh/8007/2012 | Bangladesh | August, 2012 | EPI408678 |
| B/Bangladesh/8009/2013 | Bangladesh | August, 2013 | EPI492598 |
| B/Bangladesh/3009/2013 | Bangladesh | November, 2013 | EPI510107 |
| B/Bangladesh/7751/2014 | Bangladesh | March, 2014 | EPI534046 |
| B/Bangladesh/11005/2015 | Bangladesh | October, 2015 | EPI693110 |
| B/CAMBODIA/122/2011 | Cambodia | November, 2011 | EPI370057 |
| B/CAMBODIA/2/2012 | Cambodia | April, 2012 | EPI417348 |
| B/Cambodia/U1102389/2010 | Cambodia | October, 2010 | EPI464422 |
| B/Cambodia/1287/2015 | Cambodia | December, 2015 | EPI753805 |
| B/Guangdong/GZ030/2010 | China | March, 2010 | KC986540 |
| B/Guangdong/GZ122/2010 | China | May, 2010 | KC986608 |
| B/Guangdong-Luohu/1351/2011 | China | September, 2011 | EPI366218 |
| B/Guangdong-Duanzhou/1160/2013 | China | May, 2013 | EPI477357 |
| B/Shanghai-Xuhui/1488/2012 | China | July, 2012 | EPI534527 |
| B/Shanghai-Xuhui/1497/2012 | China | July, 2012 | EPI534528 |
| B/Shanghai-Huangpu/1347/2013 | China | May, 2013 | EPI534530 |
| B/Guangdong-Zhongshan/1465/2014 | China | May, 2014 | EPI538107 |
| B/Beijing-Xicheng/12806/2015 | China | November, 2015 | EPI697300 |
| B/Kol/202/2010 | India | March, 2010 | JF693250 |
| B/Kol/222/2010 | India | March, 2010 | JF965354 |
| B/Delhi/V17/2012 | India | February, 2012 | KJ683784 |
| B/Uttrakhand/V27/2012 | India | September, 2012 | KJ683794 |
| B/Chattisgarh/V25/2012 | India | September, 2012 | KJ683792 |
| B/Delhi/V24/2012 | India | September, 2012 | KJ683791 |
| B/India/6613/2010 | India | September, 2010 | EPI302086 |
| B/India/4969/2011 | India | May, 2011 | EPI331124 |
| B/India/6245/2011 | India | October, 2011 | EPI346974 |
| B/India/4352/2014 | India | March, 2014 | EPI540550 |
| B/Indonesia/Nihrdi-Mks1003/2013 | Indonesia | August, 2013 | EPI498305 |
| B/Indonesia/Nihrdi-Dps 1144/2014 | Indonesia | March, 2014 | EPI544234 |
| B/Israel/17/2011 | Israel | December, 2011 | EPI904234 |
| B/Israel/A-6494/2015 | Israel | December, 2015 | EPI717300 |
| B/Laos/I763/2010 | Laos | September, 2010 | EPI291775 |
| B/Laos/833/2010 | Laos | September, 2010 | EPI302170 |
| B/Laos/I117/2012 | Laos | February, 2012 | EPI378250 |
| B/Laos/I1002/2012 | Laos | December, 2012 | EPI445760 |
| B/Laos/I075/2013 | Laos | January, 2013 | EPI460857 |
| B/Laos/I120/2014 | Laos | February, 2014 | EPI544172 |
| B/Laos/713/2015 | Laos | December, 2015 | EPI864592 |
| B/Laos/1100/2010 | Laos | November, 2010 | EPI1106563 |
| B/Laos/002/2011 | Laos | January, 2011 | EPI1106565 |
| B/Laos/050/2011 | Laos | February, 2011 | EPI1106567 |
| B/Malaysia/U166/2012 | Malaysia | March, 2012 | KR073339 |
| B/Malaysia/U355/2012 | Malaysia | April, 2012 | KR073354 |
| B/Malaysia/U85/2012 | Malaysia | March, 2012 | KR073331 |
| B/MALAYSIA/564/2010 | Malaysia | October, 2010 | EPI302451 |
| B/MALAYSIA/194/2010 | Malaysia | July, 2010 | EPI346036 |
| B/MALAYSIA/269/2012 | Malaysia | February, 2012 | EPI379548 |
| B/MALAYSIA/283/2012 | Malaysia | January, 2012 | EPI394365 |
| B/MALAYSIA/26/2013 | Malaysia | September, 2013 | EPI529380 |
| B/MALAYSIA/2/2014 | Malaysia | January, 2014 | EPI541304 |
| B/PHILIPPINES/2146/2010 | Philippines | November, 2010 | EPI304401 |
| B/PHILIPPINES/3971/2011 | Philippines | October, 2011 | EPI370095 |
| B/PHILIPPINES/15/2012 | Philippines | November, 2012 | EPI450353 |
| B/PHILIPPINES/3/2014 | Philippines | February, 2014 | EPI551292 |
| B/Philippines/14/2013 | Philippines | January, 2013 | EPI567287 |
| B/Incheon/8/2010 | South Korea | January, 2010 | EPI287197 |
| B/Gwangju/649/2011 | South Korea | November, 2011 | EPI363091 |
| B/Daejeon/3330/2012 | South Korea | April, 2012 | EPI393640 |
| B/Gwangju/1701/2013 | South Korea | December, 2013 | EPI510069 |
| B/Korea/2934/2014 | South Korea | January, 2014 | EPI536528 |
| B/Incheon/1611/2015 | South Korea | December, 2015 | EPI714453 |
| B/SINGAPORE/505/2010 | Singapore | February, 2010 | EPI272000 |
| B/SINGAPORE/34/2011 | Singapore | November, 2011 | EPI370036 |
| B/SINGAPORE/20/2012 | Singapore | March, 2012 | EPI394402 |
| B/SINGAPORE/KK492/2013 | Singapore | June, 2013 | EPI491350 |
| B/Singapore/EN015/2015 | Singapore | January, 2015 | EPI717591 |
| B/Thailand/CU-B2372/2010 | Thailand | June, 2010 | AFR45881 |
| B/Thailand/CU-H1400/2010 | Thailand | February, 2010 | AFR45672 |
| B/Thailand/CU-B2201/2010 | Thailand | February, 2010 | AFR45914 |
| B/Thailand/CU-B6148/2012 | Thailand | April, 2012 | AIG93827 |
| B/Thailand/CU-B2504/2010 | Thailand | July, 2010 | AFR45848 |
| B/Bangkok/SI02/2011 | Thailand | September, 2011 | AGY42433 |
| B/Thailand/CU-C1451/2010 | Thailand | December, 2010 | AFR45716 |
| B/Thailand/CU-C1768/2011 | Thailand | March, 2011 | AFR45705 |
| B/Bangkok/SI13/2012 | Thailand | August, 2012 | AGY42439 |
| B/Bangkok/SI16/2012 | Thailand | August, 2012 | AGY42442 |
| B/Vietnam/248/2010 | Vietnam | October, 2010 | EPI325303 |
| B/Vietnam/92/2011 | Vietnam | March, 2011 | EPI340849 |
| B/Vietnam/61/2012 | Vietnam | March, 2012 | EPI397155 |
| B/Vietnam/3071/2013 | Vietnam | May, 2013 | EPI465912 |
| B/Vietnam/1/2014 | Vietnam | January, 2014 | EPI630070 |
| B/Vietnam/5258/2015 | Vietnam | June, 2015 | EPI825539 |
| B/Niigata/10F529/2011 | Japan | March, 2011 | AEQ49736 |
| B/Kyoto/10K397/2011 | Japan | March, 2011 | AEQ49717 |
| B/Niigata/10F554/2011 | Japan | March, 2011 | AEQ49737 |
| B/Nagasaki/10N089/2011 | Japan | February, 2011 | AEQ49723 |
| B/Kyoto/10K474/2011 | Japan | March, 2011 | AEQ49721 |
| B/Nagasaki/13N052/2014 | Japan | February, 2014 | BAQ58756 |
| B/Kyoto/13K014/2014 | Japan | January, 2014 | BAQ58163 |
| B/Argentina/330/2010 | South America | August, 2010 | EPI291588 |
| B/Brazil/9442/2011 | South America | June, 2011 | EPI331112 |
| B/Brazil/4861/2013 | South America | August, 2013 | EPI473780 |
| B/Brazil/5137/2014 | South America | March, 2014 | EPI536584 |
| B/Brazil/83320/2015 | South America | October, 2015 | EPI721056 |
| B/Santiago/14887/2010 | South America | June, 2010 | EPI278979 |
| B/Santiago/14666/2011 | South America | July, 2011 | EPI335744 |
| B/Santiago/34960/2012 | South America | May, 2012 | EPI378192 |
| B/Santiago/49517/2013 | South America | July, 2013 | EPI468110 |
| B/Santiago/69223/2015 | South America | October, 2015 | EPI679078 |
| B/Paraguay/672/2014 | South America | November, 2011 | EPI565078 |
| B/Argentina/44/2012 | South America | July, 2012 | EPI393630 |
| B/California/12/2010 | North America | December, 2010 | EPI309168 |
| B/California/10/2011 | North America | March, 2011 | EPI325075 |
| B/California/07/2012 | North America | July, 2012 | EPI394698 |
| B/California/05/2013 | North America | April, 2013 | EPI459722 |
| B/California/08/2014 | North America | April, 2014 | EPI532934 |
| B/California/04/2015 | North America | February, 2015 | EPI582467 |
| B/Newfoundland/RV0032/2012 | North America | January, 2012 | EPI354001 |
| B/Alberta/RV2486/2014 | North America | April, 2014 | EPI550792 |
| B/Ontario/RV2438/2015 | North America | September, 2015 | EPI694954 |
| B/Mexico/4691/2010 | North America | September, 2010 | EPI294807 |
| B/Mexico/3087/2011 | North America | July, 2011 | EPI353990 |
| B/Mexico/3158/2013 | North America | October, 2013 | EPI509450 |
| B/Moscow/18/2010 | Europe | May, 2010 | EPI271921 |
| B/Saint-Petersburg/93/2011 | Europe | February, 2011 | EPI346817 |
| B/St. Petersburg/44/2012 | Europe | May, 2012 | EPI393409 |
| B/St Petersburg/121/2013 | Europe | March, 2013 | EPI450570 |
| B/England/105/2010 | Europe | November, 2010 | EPI341985 |
| B/England/168/2011 | Europe | January, 2011 | EPI326151 |
| B/England/626/2012 | Europe | December, 2012 | EPI416451 |
| B/England/226/2013 | Europe | February, 2013 | EPI445937 |
| B/England/373/2014 | Europe | March, 2014 | EPI533068 |
| B/England/473/2015 | Europe | December, 2015 | EPI811562 |
| B/Norway/970/2014 | Europe | March, 2014 | EPI540667 |
| B/Norway/1570/2015 | Europe | March, 2015 | EPI624830 |
| B/JOHANNESBURG/97/2010 | Africa | July, 2010 | EPI294090 |
| B/Johannesburg/151/2011 | Africa | June, 2011 | EPI346791 |
| B/Johannesburg/3005/2012 | Africa | May, 2012 | EPI406254 |
| B/South Africa/662/2014 | Africa | February, 2014 | EPI539454 |
| B/South Africa/5994/2015 | Africa | January, 2015 | EPI695021 |
| B/Egypt/65/2010 | Africa | December, 2010 | EPI902777 |
| B/Ghana/FS/10-4716/2010 | Africa | December, 2010 | EPI301298 |
| B/Ghana/735/2011 | Africa | May, 2011 | EPI902563 |
| B/Ghana/FS-718/2013 | Africa | July, 2013 | EPI467126 |
| B/Ghana/DILI-0434/2014 | Africa | April, 2014 | EPI540641 |
| B/SYDNEY/210/2010 | Australia | October, 2010 | EPI302466 |
| B/SYDNEY/509/2010 | Australia | October, 2010 | EPI331350 |
| B/SYDNEY/508/2010 | Australia | October, 2010 | EPI294402 |
| B/SYDNEY/21/2011 | Australia | July, 2011 | EPI346156 |
| B/SYDNEY/515/2011 | Australia | September, 2011 | EPI357557 |
| B/SYDNEY/2/2012 | Australia | February, 2012 | EPI379373 |
| B/SYDNEY/204/2012 | Australia | June, 2012 | EPI394393 |
| B/CHRISTCHURCH/1/2010 | New Zealand | June, 2010 | EPI279188 |
| B/CHRISTCHURCH/2/2010 | New Zealand | August, 2010 | EPI294352 |
| B/CHRISTCHURCH/22/2011 | New Zealand | August, 2011 | EPI346168 |
| B/CHRISTCHURCH/108/2011 | New Zealand | September, 2011 | EPI370177 |
| B/CHRISTCHURCH/62/2011 | New Zealand | July, 2011 | EPI526762 |
| B/CHRISTCHURCH/1/2012 | New Zealand | June, 2012 | EPI394384 |
| B/CHRISTCHURCH/506/2012 | New Zealand | July, 2012 | EPI394437 |
| B/New Zealand/442/2014 | New Zealand | May, 2014 | EPI540594 |
| B/New Zealand/21/2014 | New Zealand | May, 2014 | EPI538110 |
| B/Christchurch/530/2015 | New Zealand | July, 2015 | EPI957065 |
| B/Christchurch/534/2015 | New Zealand | August, 2015 | EPI675607 |
| B/New Zealand/544/2015 | New Zealand | July, 2015 | EPI649094 |
| B(Yamagata)-HA |  |  |  |
| B/Nepal/276/2011 | Nepal | August, 2011 | EPI346936 |
| B/Nepal/0544/2012 | Nepal | September, 2012 | EPI475345 |
| B/Nepal/0588/2012 | Nepal | September, 2012 | EPI475348 |
| B/Nepal/0535/2012 | Nepal | September, 2012 | EPI484582 |
| B/Nepal/49/2014 | Nepal | January, 2014 | EPI534652 |
| B/Nepal/50/2014 | Nepal | January, 2014 | EPI534654 |
| B/Nepal/545A/2014 | Nepal | April, 2014 | EPI569767 |
| B/Nepal/128/2015 | Nepal | February, 2015 | EPI722829 |
| B/Bangladesh/3204/2010 | Bangladesh | April, 2010 | EPI271617 |
| B/Bangladesh/5001/2010 | Bangladesh | September, 2010 | EPI294705 |
| B/Bangladesh/6708/2012 | Bangladesh | June, 2012 | EPI387963 |
| B/Bangladesh/9265/2012 | Bangladesh | September, 2012 | EPI406973 |
| B/Bangladesh/2988/2013 | Bangladesh | October, 2013 | EPI511844 |
| B/Bangladesh/9210/2013 | Bangladesh | October, 2013 | EPI511847 |
| B/Bangladesh/8011/2014 | Bangladesh | November, 2014 | EPI552468 |
| B/Bangladesh/5007/2014 | Bangladesh | September, 2014 | EPI553985 |
| B/Bangladesh/3014/2015 | Bangladesh | November, 2015 | EPI710684 |
| B/CAMBODIA/35/2010 | Cambodia | December, 2010 | EPI331368 |
| B/CAMBODIA/X1126361/2013 | Cambodia | November, 2013 | EPI529383 |
| B/Cambodia/538/2014 | Cambodia | May, 2014 | EPI540560 |
| B/Cambodia/1253/2014 | Cambodia | November, 2014 | EPI620355 |
| B/Cambodia/0894/2015 | Cambodia | August, 2015 | EPI620355 |
| B/Guangdong/GZ013/2010 | China | February, 2010 | KC986527 |
| B/Guangdong/GZ009/2010 | China | January, 2010 | KC986524 |
| B/Chongqing_Yuzhong/1480/2010 | China | August, 2010 | EPI331265 |
| B/Beijinghuairou/14042/2014 | China | January, 2014 | KT383648 |
| B/Beijinghuairou/14096/2014 | China | July, 2014 | KT383650 |
| B/Beijing/13-21/2013 | China | December, 2013 | KJ439759 |
| B/Beijing/13-28/2013 | China | December, 2013 | KJ439760 |
| B/Beijingchaoyang/1742/2015 | China | March, 2015 | KT383593 |
| B/Beijingchaoyang/1746/2015 | China | March, 2015 | KT383594 |
| B/Beijingchaoyang/1751/2015 | China | March, 2015 | KT383597 |
| B/Beijingdaxing/15040/2015 | China | March, 2015 | KT383576 |
| B/Beijingdongcheng/15307/2015 | China | March, 2015 | KT383633 |
| B/India/7688/2012 | India | July, 2012 | EPI394724 |
| B/India/2127/2012 | India | December, 2012 | EPI416060 |
| B/India/2152/2012 | India | December, 2012 | EPI416063 |
| B/India/5374/2014 | India | April, 2014 | EPI540547 |
| B/India/0034/2015 | India | February, 2015 | EPI737718 |
| B/India/C117501/2010 | India | October, 2010 | KF705502 |
| B/India/C117497/2010 | India | September, 2010 | KF705495 |
| B/Haryana/Y42/2013 | India | February, 2013 | KJ683809 |
| B/Haryana/Y44/2013 | India | February, 2013 | KJ683811 |
| B/Indonesia/Nihrdi-Dps1052/2013 | Indonesia | September, 2013 | EPI498321 |
| B/Indonesia/Nihrdi-Plk 198/2014 | Indonesia | February, 2014 | EPI544232 |
| B/Indonesia/Nihrdi-Pdg 138/2014 | Indonesia | December, 2014 | EPI544394 |
| B/Indonesia/Nihrdi-Mlg157/2015 | Indonesia | April, 2015 | EPI651898 |
| B/Israel/20/2011 | Israel | December, 2011 | EPI352844 |
| B/Israel/Z-1510/2014 | Israel | March, 2014 | EPI539432 |
| B/Israel/O-6350/2014 | Israel | November, 2014 | EPI574786 |
| B/Israel/1/2013 | Israel | January, 2013 | EPI431350 |
| B/Laos/021/2010 | Laos | October, 2010 | EPI309197 |
| B/Laos/I917/2011 | Laos | November, 2011 | EPI368635 |
| B/Laos/774/2012 | Laos | October, 2012 | EPI416071 |
| B/Laos/O128/2012 | Laos | December, 2012 | EPI445767 |
| B/Laos/015/2013 | Laos | May, 2013 | EPI482840 |
| B/Laos/I1144/2013 | Laos | November, 2013 | EPI548540 |
| B/Laos/O566/2014 | Laos | November, 2014 | EPI569783 |
| B/Laos/O573/2014 | Laos | November, 2014 | EPI621519 |
| B/Laos/99/2015 | Laos | February, 2015 | EPI664170 |
| B/MALAYSIA/207/2010 | Malaysia | February, 2010 | EPI294187 |
| B/MALAYSIA/977/2011 | Malaysia | May, 2011 | EPI357579 |
| B/MALAYSIA/412/2012 | Malaysia | May, 2012 | EPI498447 |
| B/MALAYSIA/878/2012 | Malaysia | July, 2012 | EPI450338 |
| B/MALAYSIA/16/2013 | Malaysia | March, 2013 | EPI477626 |
| B/MALAYSIA/27/2013 | Malaysia | October, 2013 | EPI529354 |
| B/MALAYSIA/8/2014 | Malaysia | March, 2014 | EPI541282 |
| B/MALAYSIA/14/2014 | Malaysia | May, 2014 | EPI551817 |
| B/Malaysia/14MA061/2014 | Malaysia | December, 2014 | EPI629307 |
| B/Malaysia/14MA062/2014 | Malaysia | December, 2014 | EPI629309 |
| B/Malaysia/14MA086/2015 | Malaysia | February, 2015 | EPI629314 |
| B/Malaysia/U3636/2014 | Malaysia | March, 2014 | KR073478 |
| B/Malaysia/U3630/2014 | Malaysia | March, 2014 | KR073477 |
| B/PHILIPPINES/3976/2011 | Philippines | October, 2011 | EPI370092 |
| B/PHILIPPINES/1/2012 | Philippines | January, 2012 | EPI417386 |
| B/PHILIPPINES/9/2013 | Philippines | February, 2013 | EPI491339 |
| B/Philippines/16/2014 | Philippines | September, 2014 | EPI562299 |
| B/Philippines/11/2014 | Philippines | October, 2014 | EPI630037 |
| B/Philippines/17/2014 | Philippines | November, 2014 | EPI630045 |
| B/Gyeonggi/3008/2012 | South Korea | April, 2012 | EPI393637 |
| B/Busan/1676/2013 | South Korea | November, 2013 | EPI511841 |
| B/Gangwon/2004/2014 | South Korea | December, 2014 | EPI562688 |
| B/Daejeon/1999/2014 | South Korea | December, 2014 | EPI565177 |
| B/SINGAPORE/340/2010 | Singapore | September, 2010 | EPI331315 |
| B/SINGAPORE/20/2011 | Singapore | October, 2011 | EPI370117 |
| B/SINGAPORE/10/2012 | Singapore | June, 2012 | EPI394410 |
| B/SINGAPORE/11/2012 | Singapore | June, 2012 | EPI394413 |
| B/Singapore/GP127/2015 | Singapore | January, 2015 | EPI717589 |
| B/Phuket/77/2012 | Thailand | April, 2012 | EPI378210 |
| B/PHUKET/423/2012 | Thailand | December, 2012 | EPI450356 |
| B/PHUKET/266/2014 | Thailand | May, 2014 | EPI541351 |
| B/Bangkok/42/2015 | Thailand | June, 2015 | EPI644135 |
| B/Bangkok/SI06/2011 | Thailand | October, 2011 | KF699253 |
| B/Bangkok/SI65/2013 | Thailand | October, 2013 | KX058953 |
| B/Bangkok/SI100/2014 | Thailand | March, 2014 | KX058985 |
| B/Bangkok/SI64/2013 | Thailand | January, 2013 | KX058952 |
| B/PHUKET/3073/2013 | Thailand | November, 2013 | EPI544264 |
| B/Vietnam/VP12-98/2012 | Vietnam | October, 2012 | EPI463629 |
| B/Vietnam/426/2013 | Vietnam | October, 2013 | EPI499214 |
| B/Vietnam/368/2014 | Vietnam | December, 2014 | EPI565089 |
| B/Vietnam/3/2014 | Vietnam | April, 2014 | EPI630076 |
| B/Vietnam/277/2015 | Vietnam | October, 2015 | EPI737820 |
| B/SAPPORO/3/2010 | Japan | March, 2010 | EPI272496 |
| B/TOKYO/98724/2011 | Japan | November, 2011 | EPI356963 |
| B/SAPPORO/40/2012 | Japan | March, 2012 | EPI382033 |
| B/NIIGATA-C/41/2012 | Japan | May, 2012 | EPI393430 |
| B/KYOTO/10/2012 | Japan | March, 2012 | EPI394950 |
| B/Sapporo/21/2013 | Japan | May, 2013 | EPI468074 |
| B/Niigata/13F498/2014 | Japan | March, 2014 | LC033225 |
| B/Gunma/13G020/2014 | Japan | March, 2014 | LC032929 |
| B/Niigata/13F336/2014 | Japan | March, 2014 | LC033209 |
| B/Hokkaido/13H045/2014 | Japan | February, 2014 | LC033017 |
| B/Nagasaki/13N098/2014 | Japan | March, 2014 | LC033385 |
| B/Nagasaki/14N134/2015 | Japan | June, 2015 | EPI1215462 |
| B/Nagasaki/14N135/2015 | Japan | July, 2015 | EPI1215464 |
| B/Brazil/1000/2010 | South America | July, 2010 | EPI291620 |
| B/Natal/119888/2012 | South America | July, 2012 | EPI394721 |
| B/Brazil/325/2013 | South America | May, 2013 | EPI465935 |
| B/Brazil/2282/2014 | South America | July, 2014 | EPI543660 |
| B/Brazil/118/2015 | South America | April, 2014 | EPI642591 |
| B/Santiago/39712/2012 | South America | June, 2012 | EPI393652 |
| B/Santiago/47508/2012 | South America | June, 2012 | EPI395184 |
| B/Paraguay/1453/2010 | South America | August, 2010 | EPI302053 |
| B/Paraguay/022/2013 | South America | June, 2013 | EPI482837 |
| B/Paraguay/2429/2014 | South America | November, 2014 | EPI562679 |
| B/Paraguay/4726/2015 | South America | June, 2015 | EPI642974 |
| B/California/06/2010 | North America | November, 2010 | EPI301235 |
| B/California/12/2011 | North America | November, 2011 | EPI348447 |
| B/California/08/2012 | North America | August, 2012 | EPI397766 |
| B/California/04/2013 | North America | January, 2013 | EPI457583 |
| B/California/31/2014 | North America | November, 2014 | EPI568869 |
| B/California/08/2015 | North America | March, 2015 | EPI586724 |
| B/Mexico/6970/2012 | North America | May, 2012 | EPI391311 |
| B/Mexico/2487/2014 | North America | November, 2014 | EPI568968 |
| B/Mexico/1447/2015 | North America | April, 2015 | EPI642742 |
| B/Massachusetts/02/2012 | North America | March, 2012 | EPI904337 |
| B/Wisconsin/01/2010 | North America | February, 2010 | EPI363743 |
| B/England/110/2010 | Europe | November, 2010 | EPI341988 |
| B/England/254/2011 | Europe | October, 2011 | EPI346821 |
| B/England/706/2012 | Europe | December, 2012 | EPI416453 |
| B/England/709/2012 | Europe | December, 2012 | EPI416457 |
| B/England/286/2013 | Europe | February, 2013 | EPI445939 |
| B/England/372/2014 | Europe | March, 2014 | EPI533066 |
| B/England/515/2014 | Europe | October, 2014 | EPI811543 |
| B/England/54220123/2015 | Europe | October, 2015 | EPI679311 |
| B/England/170/2010 | Europe | December, 2010 | EPI902709 |
| B/St. Petersburg/24/2012 | Europe | April, 2012 | EPI379932 |
| B/Saint-Petersburg/23/2013 | Europe | February, 2013 | EPI445965 |
| B/Saint-Petersburg/76/2014 | Europe | April, 2014 | EPI536463 |
| B/Ghana/FS-2112/2010 | Africa | April, 2010 | EPI271909 |
| B/Ghana/FS-11-1994/2011 | Africa | November, 2011 | EPI352451 |
| B/Ghana/DILI-15-0295/2015 | Africa | April, 2015 | EPI624774 |
| B/Ghana/FS12-808/2012 | Africa | June, 2012 | EPI904291 |
| B/Egypt/3802/2014 | Africa | December, 2015 | EPI693056 |
| B/Egypt/4125/2014 | Africa | June, 2014 | EPI717321 |
| B/Johannesburg/40/2010 | Africa | July, 2010 | EPI287074 |
| B/South Africa/4437/2013 | Africa | June, 2013 | EPI504820 |
| B/South Africa/4380/2014 | Africa | June, 2014 | EPI539452 |
| B/South Africa/5935/2015 | Africa | September, 2015 | EPI695205 |
| B/Johannesburg/3559/2012 | Africa | June, 2012 | EPI904340 |
| B/Victoria/502/2014 | Australia | August, 2014 | CY202017 |
| B/Victoria/504/2014 | Australia | August, 2014 | CY202001 |
| B/SYDNEY/5/2012 | Australia | February, 2012 | EPI379439 |
| B/SYDNEY/19/2013 | Australia | June, 2013 | EPI491157 |
| B/SYDNEY/1015/2013 | Australia | October, 2013 | EPI526722 |
| B/SYDNEY/40/2013 | Australia | November, 2013 | EPI526822 |
| B/SYDNEY/1002/2014 | Australia | July, 2014 | EPI551324 |
| B/Sydney/39/2014 | Australia | December, 2015 | EPI561915 |
| B/Sydney/5/2015 | Australia | April, 2015 | EPI636553 |
| B/Sydney/1031/2015 | Australia | August, 2015 | EPI675657 |
| B/SOUTH AUCKLAND/5/2011 | New Zealand | June, 2011 | EPI346060 |
| B/CHRISTCHURCH/505/2012 | New Zealand | July, 2012 | EPI394428 |
| B/SOUTH AUCKLAND/30/2012 | New Zealand | July, 2012 | EPI417392 |
| B/SOUTH AUCKLAND/8/2012 | New Zealand | June, 2012 | EPI417395 |
| B/New Zealand/484/2014 | New Zealand | June, 2014 | EPI540553 |
| B/New Zealand/26/2014 | New Zealand | May, 2014 | EPI540556 |
| B/Christchurch/2/2015 | New Zealand | February, 2015 | EPI636396 |
| B/CHRISTCHURCH/502/2013 | New Zealand | May, 2013 | EPI466145 |
| B/CHRISTCHURCH/3/2013 | New Zealand | June, 2013 | EPI491383 |
| B/CHRISTCHURCH/500/2014 | New Zealand | March, 2014 | EPI541235 |
