# Supplementary material for: Phylogeographic analysis of human influenza A and B viruses in Myanmar, 2010–2015
Source: PLoS One. 2019 Jan 10;14(1):e0210550. doi: 10.1371/journal.pone.0210550 (PMC6328249; doi:10.1371/journal.pone.0210550)
Supplement: S4 Table — (DOCX) [file pone.0210550.s004.docx]

S4 Table. Yearly distribution of samples and Influenza virus isolates in Yangon, Myanmar, 2010-2015

|  |  |  | |  | | | 2010 | | |  | | | 2011 | | |  | | | 2012 | | |  | | | 2013 | | |  | | | 2014 | | |  | | | 2015 | | |  | | | Total | | |  |
| --- | --- | --- | --- | --- | --- | --- | --- | --- | --- | --- | --- | --- | --- | --- | --- | --- | --- | --- | --- | --- | --- | --- | --- | --- | --- | --- | --- | --- | --- | --- | --- | --- | --- | --- | --- | --- | --- | --- | --- | --- | --- | --- | --- | --- | --- | --- |
|  |  |  | |  | | | n=514 | | |  | | | n=333 | | |  | | | n=361 | | |  | | | n=261 | | |  | | | n=183 | | |  | | | n=121 | | |  | | | n=1773 | | |  |
| Influenza RDT*- positive samples | | | | | 509 | | |  | | | 278 | | |  | | | 335 | | |  | | | 150 | | |  | | | 126 | | |  | | | 37 | | |  | | | 1435 | | |  | | |
| Virus isolate (+) | |  | |  | | | 267 | | (52.5%) | | | | 55 | | | (19.8%) | | | 61 | | | (18.2%) | | | 61 | | | (40.7%) | | | 118 | | | (93.7%) | | | 23 | | | (62.2%) | | | 585 | | | (40.8%) |
|  | A(H1N1pdm)09 | |  | | | 192 | | (71.9%) | | | | 0 | | | (0.0%) | | | 10 | | | (16.4%) | | | 0 | | | (0.0%) | | | 26 | | | (22.0%) | | | 16 | | | (69.6%) | | | 244 | | | (41.7%) | |
|  | A (H3N2) | |  | | | 10 | | (3.7%) | | | | 46 | | | (16.5%) | | | 0 | | | (0.0%) | | | 61 | | | (100.0%) | | | 33 | | | (28.0%) | | | 10 | | | (43.5%) | | | 160 | | | (27.4%) | |
|  | B (Victoria) | |  | | | 75 | | (28.1%) | | | | 8 | | | (2.9%) | | | 1 | | | (1.6%) | | | 0 | | | (0.0%) | | | 0 | | | (0.0%) | | | 0 | | | (0.0%) | | | 84 | | | (14.4%) | |
|  | B(Yamagata) | |  | | | 0 | | (0.0%) | | | | 0 | | | (0.0%) | | | 1 | | | (1.6%) | | | 0 | | | (0.0%) | | | 55 | | | (46.6%) | | | 0 | | | (0.0%) | | | 56 | | | (9.6%) | |

*RDT- Rapid Diagnostic Test
