# Supplementary material for: Phylogeographic analysis of human influenza A and B viruses in Myanmar, 2010–2015
Source: PLoS One. 2019 Jan 10;14(1):e0210550. doi: 10.1371/journal.pone.0210550 (PMC6328249; doi:10.1371/journal.pone.0210550)
Supplement: S5 Table — (DOCX) [file pone.0210550.s005.docx]

S5 Table. Yearly distribution of samples and Influenza virus isolates in Pyinmana, Myanmar,2010-2015

|  |  |  |  | 2010 | |  | | 2011 | |  | | 2012 | |  | | 2013 | |  | | 2014 | |  | | 2015 | |  | | Total | |  | |  |
| --- | --- | --- | --- | --- | --- | --- | --- | --- | --- | --- | --- | --- | --- | --- | --- | --- | --- | --- | --- | --- | --- | --- | --- | --- | --- | --- | --- | --- | --- | --- | --- | --- |
|  |  |  |  | n=180 | |  | | n=123 | |  | | n=186 | |  | | n=53 | |  | | n=139 | |  | | n=228 | |  | | n=909 | |  | |  |
| Influenza RDT*- positive samples | | | | 180 | |  | | 123 | |  | | 185 | |  | | 53 | |  | | 139 | |  | | 94 | |  | | 774 | |  | |  |
| Virus isolate (+) | |  |  | 78 | | (43.3%) | | 119 | | (96.7%) | | 94 | | (50.8%) | | 2 | | (3.8%) | | 92 | | (66.2%) | | 35 | | (37.2%) | | 420 | | (54.3%) | |  |
|  | A (H1N1 pdm)09 | | |  | 36 | | (46.2%) | | 0 | | (0.0%) | | 8 | | (8.5%) | | 1 | | (1.9%) | | 20 | | (21.7%) | | 15 | | (42.9%) | | 80 | | (19.0%) | |
|  | A (H3N2) | | | 8 | | (10.3%) | | 119 | | (100.0%) | | 0 | | (0.0%) | | 1 | | (1.9%) | | 18 | | (19.6%) | | 20 | | (57.1%) | | 166 | | (39.5%) | |  |
|  | B (Victoria) | |  | 34 | | (43.6%) | | 0 | | (0.0%) | | 12 | | (12.8%) | | 0 | | (0.0%) | | 0 | | (0.0%) | | 0 | | (0.0%) | | 46 | | (11.0%) | |  |
|  | B(Yamagata) | | | 0 | | (0.0%) | | 0 | | (0.0%) | | 70 | | (74.5%) | | 0 | | (0.0%) | | 28 | | (30.4%) | | 0 | | (0.0%) | | 98 | | (23.3%) | |  |

*RDT- Rapid Diagnostic Test
