# Supplementary material for: Phylogeographic analysis of human influenza A and B viruses in Myanmar, 2010–2015
Source: PLoS One. 2019 Jan 10;14(1):e0210550. doi: 10.1371/journal.pone.0210550 (PMC6328249; doi:10.1371/journal.pone.0210550)
Supplement: S6 Table — (DOCX) [file pone.0210550.s006.docx]

S6 Table. Yearly distribution of samples and Influenza virus isolates in Pyin Oo Lwin, Myanmar, 2010-2015

|  |  |  | |  | 2010 |  | 2011 |  | 2012 |  | 2013 |  | 2014 |  | 2015 |  | Total |  |
| --- | --- | --- | --- | --- | --- | --- | --- | --- | --- | --- | --- | --- | --- | --- | --- | --- | --- | --- |
|  |  |  | |  | n=0 |  | n=0 |  | n=0 |  | n=0 |  | n=211 |  | n=0 |  | n=211 |  |
| Influenza RDT*- positive samples | | | | | - |  | - |  | - |  | - |  | 178 |  | - |  | 178 |  |
| Virus isolate (+) | | |  |  |  |  |  |  |  |  |  |  | 125 | (70.2%) |  |  | 125 | (70.2%) |
|  | A (H1N1pdm)09 | | |  | - | - | - | - | - | - | - | - | 45 | (36.0%) | - | - | 45 | (36.0%) |
|  | A (H3N2) | | | | - | - | - | - | - | - | - | - | 4 | (3.2%) | - | - | 4 | (3.2%) |
|  | B (Victoria) | | |  | - | - | - | - | - | - | - | - | 0 | (0.0%) | - | - | 0 | (0.0%) |
|  | B　(Yamagata) | | | | - | - | - | - | - | - | - | - | 29 | (23.2%) | - | - | 29 | (23.2%) |

*RDT- Rapid Diagnostic Test
