# Supplementary material for: Phylogeographic analysis of human influenza A and B viruses in Myanmar, 2010–2015
Source: PLoS One. 2019 Jan 10;14(1):e0210550. doi: 10.1371/journal.pone.0210550 (PMC6328249; doi:10.1371/journal.pone.0210550)
Supplement: S7 Table — (DOCX) [file pone.0210550.s007.docx]

S7 Table. Bayes factor and Posterior Probability of migration events of influenza viruses A(H1N1)pdm09, A(H3N2), and B, Myanmar and other countries, 2010-2015

| \| LOCATIONS \| \| BAYES FACTOR \| POSTERIOR PROBABILITY \| \| \| --- \| --- \| --- \| --- \| --- \| \| FROM \| TO \|  \| \|  \| \| **A(H1N1)pdm09** \| \|  \| \|  \| \| Bangladesh \| China \| 39.26 \| \| 0.78 \| \| India \| Japan \| 2421.22 \| \| 1 \| \| India \| China \| 3.17 \| \| 0.22 \| \| Europe \| Japan \| 3422.78 \| \| 1 \| \| Australia \| Japan \| 59.61 \| \| 0.84 \| \| Australia \| South America \| 43.36 \| \| 0.8 \| \| Australia \| Myanmar \| 209.98 \| \| 0.95 \| \| Australia \| Singapore \| 3.1 \| \| 0.22 \| \| Lebanon \| Japan \| 363.06 \| \| 0.97 \| \| North America \| Japan \| 58365.64 \| \| 1 \| \| Japan \| South America \| 14583.05 \| \| 1 \| \| Japan \| Myanmar \| 25.87 \| \| 0.7 \| \| Japan \| Vietnam \| 2421.22 \| \| 1 \| \| Japan \| New Zealand \| 58365.64 \| \| 1 \| \| Japan \| Africa \| 978.29 \| \| 0.99 \| \| Japan \| Cambodia \| 388.69 \| \| 0.97 \| \| Japan \| Malaysia \| 58365.64 \| \| 1 \| \| Japan \| Israel \| 40.56 \| \| 0.78 \| \| Japan \| Indonesia \| 58365.64 \| \| 1 \| \| Japan \| China \| 58365.64 \| \| 1 \| \| Japan \| Philippines \| 172.43 \| \| 0.94 \| \| Japan \| Thailand \| 1156.39 \| \| 0.99 \| \| Japan \| Nepal \| 4853.58 \| \| 1 \| \| Japan \| South Korea \| 250.63 \| \| 0.96 \| \| Japan \| Africa \| 58365.64 \| \| 1 \| \| Japan \| Afghanistan \| 1111.48 \| \| 0.99 \| \| Laos \| China \| 7.16 \| \| 0.39 \| \|  \|  \|  \| \|  \| \| **A(H3N2)** \| \|  \| \|  \| \| Japan \| Israel \| 24.86 \| \| 0.7 \| \| Europe \| Laos \| 8.44 \| \| 0.44 \| \| Europe \| Australia \| 95840.44 \| \| 1 \| \| Europe \| China \| 25.49 \| \| 0.71 \| \| Laos \| Australia \| 65.48 \| \| 0.86 \| \| Laos \| Thailand \| 9.15 \| \| 0.46 \| \| India \| Australia \| 15964.53 \| \| 1 \| \| India \| South Korea \| 9.74 \| \| 0.48 \| \| North America \| Australia \| 67.66 \| \| 0.86 \| \| North America \| South America \| 3.53 \| \| 0.25 \| \| Africa \| Australia \| 31939.71 \| \| 1 \| \| Australia \| Cambodia \| 4.49 \| \| 0.3 \| \| Australia \| Philippine \| 3823.39 \| \| 1 \| \| Australia \| Indonesia \| 11970.74 \| \| 1 \| \| Australia \| South America \| 95840.44 \| \| 1 \| \| Australia \| China \| 4.59 \| \| 0.3 \| \| Australia \| Bangladesh \| 57.72 \| \| 0.84 \| \| Australia \| Myanmar \| 95840.44 \| \| 1 \| \| Australia \| New Zealand \| 140.06 \| \| 0.93 \| \| Australia \| Thailand \| 46.75 \| \| 0.81 \| \| Australia \| Israel \| 95840.44 \| \| 1 \| \| Australia \| Malaysia \| 49.07 \| \| 0.82 \| \| Australia \| Afghanistan \| 461.52 \| \| 0.98 \| \| Australia \| Nepal \| 95840.44 \| \| 1 \| \| Australia \| Vietnam \| 25.3 \| \| 0.7 \| \| Australia \| South Korea \| 31.17 \| \| 0.75 \| \| Australia \| Singapore \| 79.86 \| \| 0.88 \| \| Cambodia \| Israel \| 38.33 \| \| 0.78 \| \| Lebanon \| China \| 9.3 \| \| 0.47 \| \| Indonesia \| Bangladesh \| 5.31 \| \| 0.33 \| \| South America \| Vietnam \| 4.11 \| \| 0.28 \| \| Thailand \| Israel \| 9.1 \| \| 0.46 \| \|  \|  \|  \| \|  \| \| **Influenza B** \| \|  \| \|  \| \| Singapore \| Myanmar \| 948.13 \| \| 0.99 \| \| Singapore \| Africa \| 12.64 \| \| 0.57 \| \| Singapore \| Europe \| 41372.87 \| \| 1 \| \| Myanmar \| Europe \| 41372.87 \| \| 1 \| \| Japan \| Laos \| 3.09 \| \| 0.24 \| \| Japan \| Europe \| 41372.87 \| \| 1 \| \| South Korea \| Europe \| 716.36 \| \| 0.99 \| \| China \| Europe \| 41372.87 \| \| 1 \| \| New Zealand \| Europe \| 2059.48 \| \| 1 \| \| New Zealand \| Thailand \| 3.2 \| \| 0.25 \| \| India \| Europe \| 20681.61 \| \| 1 \| \| Malaysia \| Europe \| 4.06 \| \| 0.3 \| \| Malaysia \| Vietnam \| 12.99 \| \| 0.57 \| \| Laos \| Europe \| 4.74 \| \| 0.33 \| \| Africa \| Europe \| 41372.87 \| \| 1 \| \| North America \| Europe \| 21.85 \| \| 0.69 \| \| North America \| Israel \| 25.63 \| \| 0.73 \| \| Cambodia \| South America \| 3.19 \| \| 0.25 \| \| Cambodia \| Europe \| 41372.87 \| \| 1 \| \| South America \| Bangladesh \| 756.69 \| \| 0.99 \| \| South America \| Indonesia \| 41372.87 \| \| 1 \| \| South America \| Australia \| 41372.87 \| \| 1 \| \| South America \| Philippines \| 20681.61 \| \| 1 \| \| South America \| Vietnam \| 1714.62 \| \| 0.99 \| \| South America \| Thailand \| 41372.87 \| \| 1 \| \| South America \| Nepal \| 13784.52 \| \| 1 \| \| South America \| Israel \| 41372.87 \| \| 1 \| \| Europe \| Bangladesh \| 416.98 \| \| 0.98 \| |  |
| --- | --- | --- | --- | --- | --- | --- | --- | --- | --- | --- | --- | --- | --- | --- | --- | --- | --- | --- | --- | --- | --- | --- | --- | --- | --- | --- | --- | --- | --- | --- | --- | --- | --- | --- | --- | --- | --- | --- | --- | --- | --- | --- | --- | --- | --- | --- | --- | --- | --- | --- | --- | --- | --- | --- | --- | --- | --- | --- | --- | --- | --- | --- | --- | --- | --- | --- | --- | --- | --- | --- | --- | --- | --- | --- | --- | --- | --- | --- | --- | --- | --- | --- | --- | --- | --- | --- | --- | --- | --- | --- | --- | --- | --- | --- | --- | --- | --- | --- | --- | --- | --- | --- | --- | --- | --- | --- | --- | --- | --- | --- | --- | --- | --- | --- | --- | --- | --- | --- | --- | --- | --- | --- | --- | --- | --- | --- | --- | --- | --- | --- | --- | --- | --- | --- | --- | --- | --- | --- | --- | --- | --- | --- | --- | --- | --- | --- | --- | --- | --- | --- | --- | --- | --- | --- | --- | --- | --- | --- | --- | --- | --- | --- | --- | --- | --- | --- | --- | --- | --- | --- | --- | --- | --- | --- | --- | --- | --- | --- | --- | --- | --- | --- | --- | --- | --- | --- | --- | --- | --- | --- | --- | --- | --- | --- | --- | --- | --- | --- | --- | --- | --- | --- | --- | --- | --- | --- | --- | --- | --- | --- | --- | --- | --- | --- | --- | --- | --- | --- | --- | --- | --- | --- | --- | --- | --- | --- | --- | --- | --- | --- | --- | --- | --- | --- | --- | --- | --- | --- | --- | --- | --- | --- | --- | --- | --- | --- | --- | --- | --- | --- | --- | --- | --- | --- | --- | --- | --- | --- | --- | --- | --- | --- | --- | --- | --- | --- | --- | --- | --- | --- | --- | --- | --- | --- | --- | --- | --- | --- | --- | --- | --- | --- | --- | --- | --- | --- | --- | --- | --- | --- | --- | --- | --- | --- | --- | --- | --- | --- | --- | --- | --- | --- | --- | --- | --- | --- | --- | --- | --- | --- | --- | --- | --- | --- | --- | --- | --- | --- | --- | --- | --- | --- | --- | --- | --- | --- | --- | --- | --- | --- | --- | --- | --- | --- | --- | --- | --- | --- | --- | --- | --- | --- | --- | --- | --- | --- | --- | --- | --- | --- | --- | --- | --- | --- | --- | --- | --- | --- | --- | --- | --- | --- | --- | --- | --- | --- | --- | --- | --- | --- | --- | --- | --- | --- | --- | --- | --- | --- | --- | --- | --- | --- | --- | --- | --- | --- | --- | --- | --- | --- | --- | --- | --- | --- | --- | --- | --- | --- | --- | --- | --- | --- | --- | --- | --- | --- | --- | --- | --- | --- | --- | --- | --- | --- | --- | --- | --- | --- | --- | --- | --- | --- | --- | --- | --- | --- | --- | --- | --- | --- | --- | --- | --- | --- | --- | --- | --- | --- | --- | --- | --- | --- | --- | --- | --- | --- | --- | --- | --- | --- | --- | --- | --- | --- | --- | --- | --- | --- | --- | --- | --- | --- | --- | --- | --- | --- | --- | --- | --- | --- | --- |
