# Supplementary figures and images for: Phylogeographic analysis of human influenza A and B viruses in Myanmar, 2010–2015
Source: PLoS One. 2019 Jan 10;14(1):e0210550. doi: 10.1371/journal.pone.0210550 (PMC6328249; doi:10.1371/journal.pone.0210550)

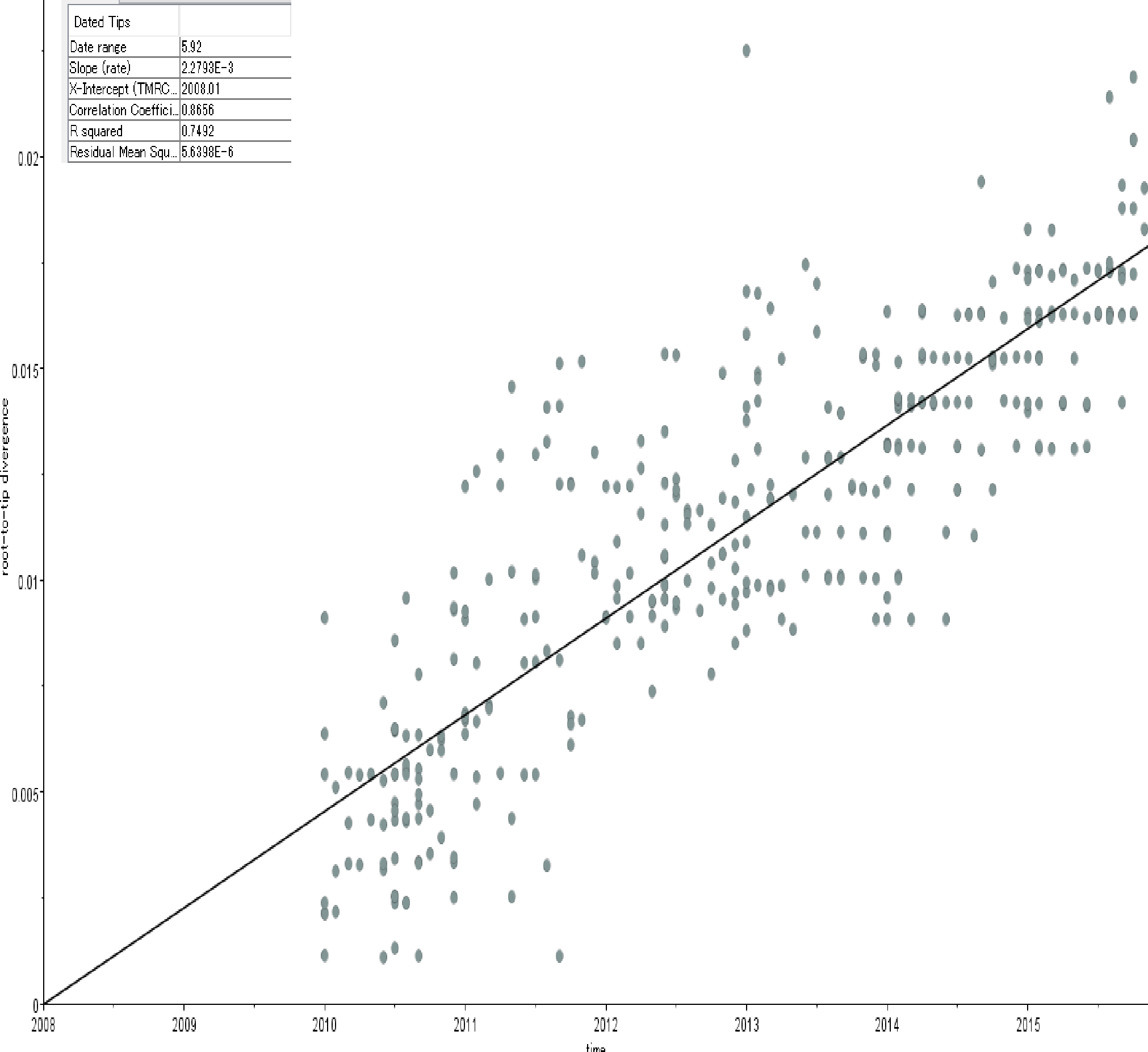

Supplement: S1 Fig — The coefficient correlation of 0.87 and R2 of 0.75 show a positive correction between genetic divergence and sampling time. The data set of A(H1N1)pdm09-HA gene is therefore fit for phylogenetic molecular clock analysis in BEAST. (TIF) [file pone.0210550.s008.tif]

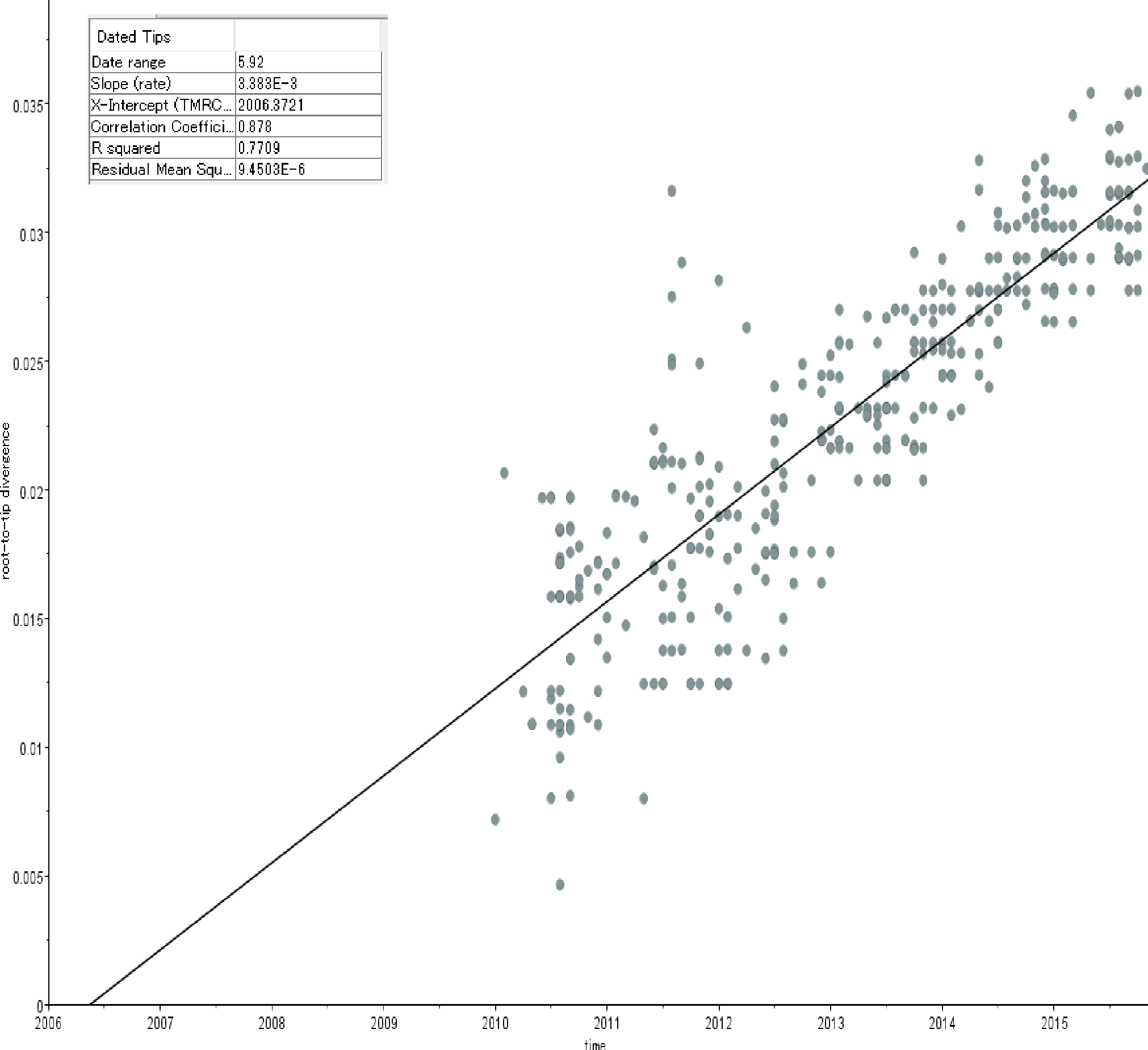

Supplement: S2 Fig — The coefficient correlation of 0.88 and R2 of 0.77 show a positive correction between genetic divergence and sampling time. The data set of A(H3N2)-HA gene is therefore fit for phylogenetic molecular clock analysis in BEAST. (TIF) [file pone.0210550.s009.tif]

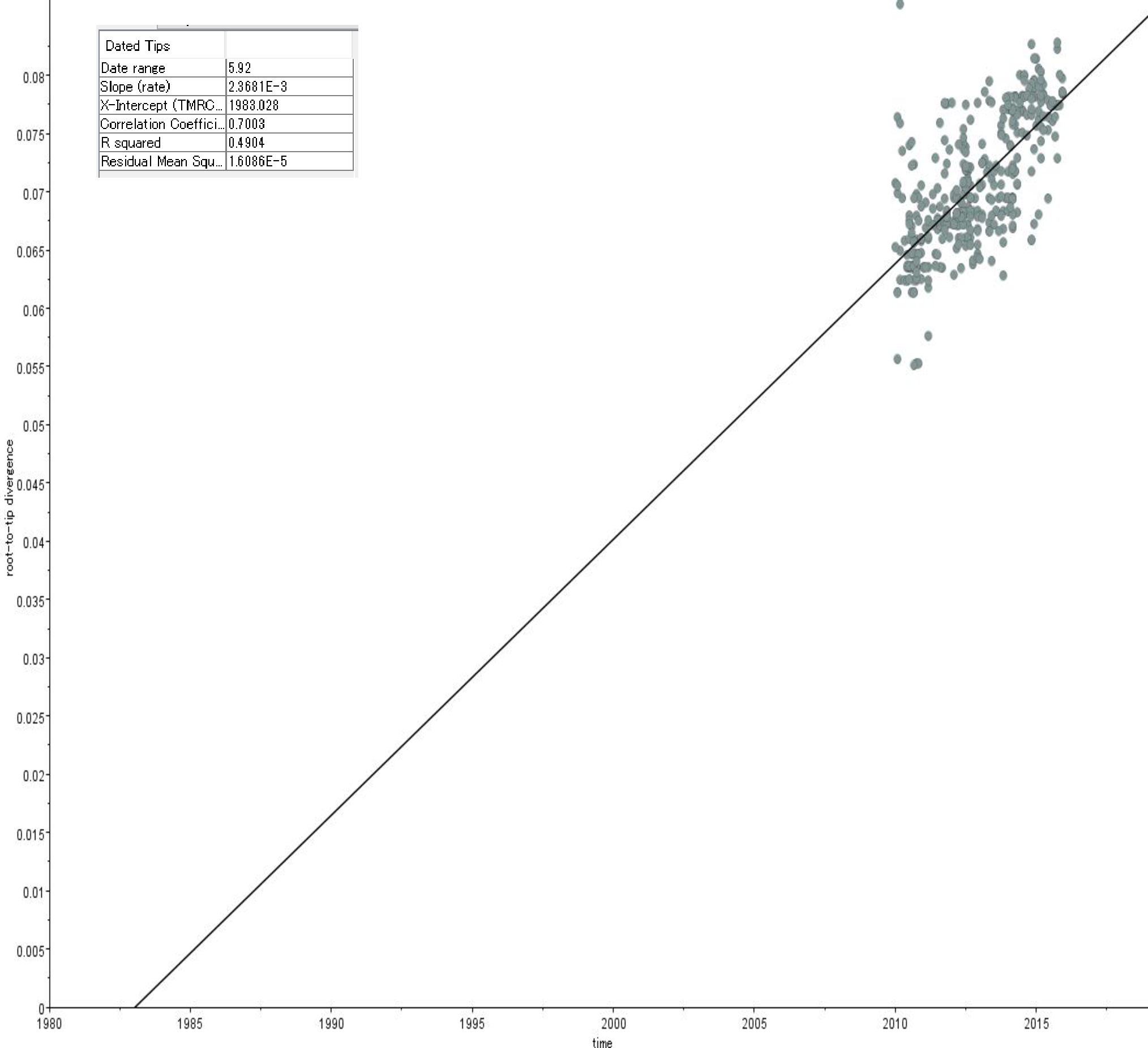

Supplement: S3 Fig — The coefficient correlation of 0.70 and R2 of 0.49 show a positive correction between genetic divergence and sampling time. The data set of influenza B-HA gene is therefore fit for gene phylogenetic molecular clock analysis in BEAST. (TIF) [file pone.0210550.s010.tif]

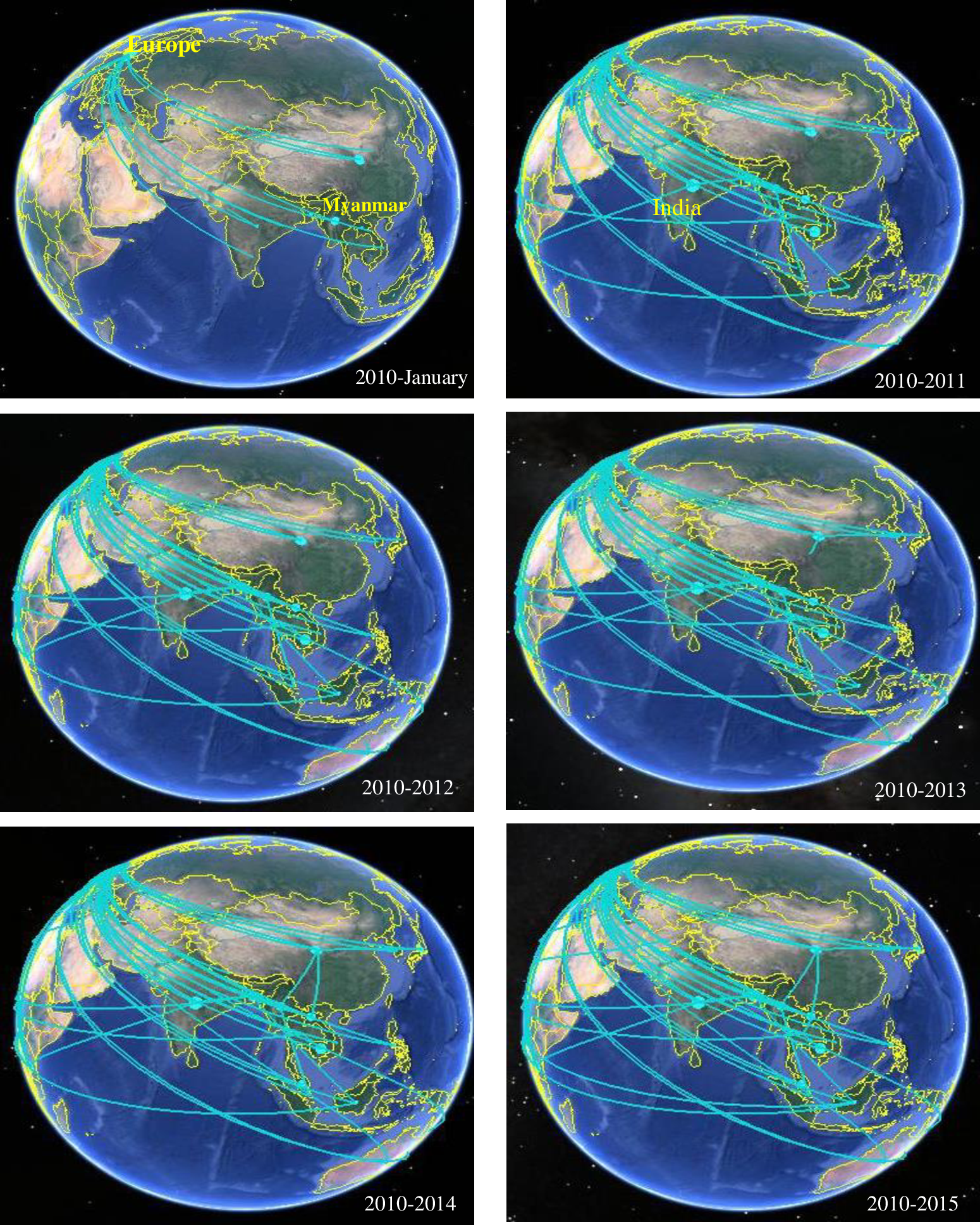

Supplement: S4 Fig — The snapshots show the dispersal pattern of A(H1N1)pdm09 virus of HA from 2010 to 2015. Connections between different countries represent branches in the MCC tree along which the relevant location transition occurs. Location circle diameters are proportional to square root of the number of MCC branches maintaining a particular location state at each time point. The blue gradients show the relative age of transitions for HA. This map is produced by satellite pictures made available in Google Earth. (TIF) [file pone.0210550.s011.tif]

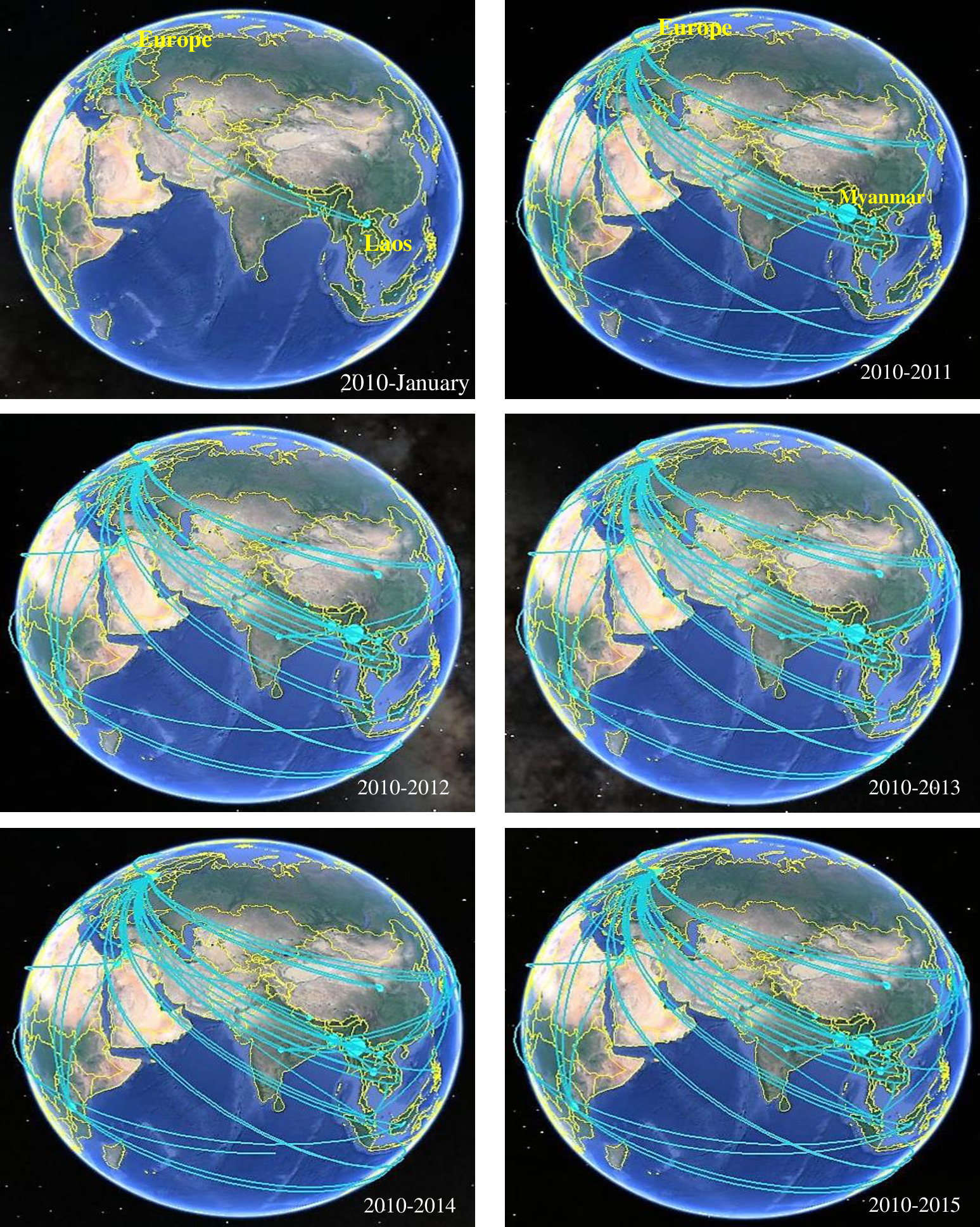

Supplement: S5 Fig — The snapshots show the dispersal pattern of A(H3N2) virus of HA from 2010 to 2015. Connections between different countries represent branches in the MCC tree along which the relevant location transition occurs. Location circle diameters are proportional to square root of the number of MCC branches maintaining a particular location state at each time point. The blue color gradients show the relative age of transitions for HA. This map is produced by satellite pictures made available in Google Earth. (TIF) [file pone.0210550.s012.tif]

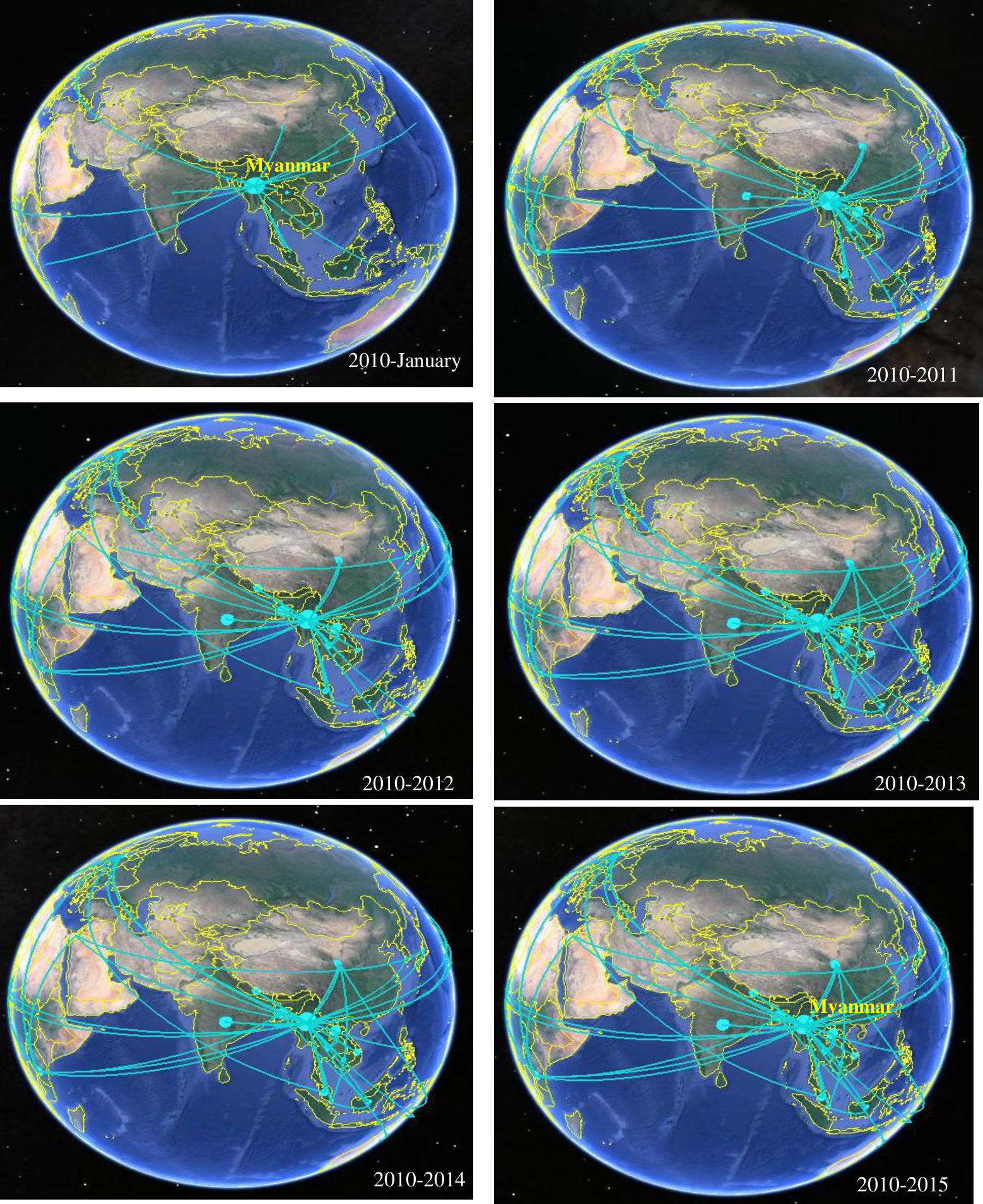

Supplement: S6 Fig — The snapshots show the dispersal pattern of influenza B virus of HA from 2010 to 2015. Connections between different countries represent branches in the MCC tree along which the relevant location transition occurs. Location circle diameters are proportional to square root of the number of MCC branches maintaining a particular location state at each time point. The blue color gradients show the relative age of transitions for HA. This map is produced by satellite pictures made available in Google Earth. (TIF) [file pone.0210550.s013.tif]
